# Novel Marine Fungus-Derived Mycophenolic Acids That Inhibit Acute Myeloid Leukemia Cell Proliferation

**DOI:** 10.3390/md24030108

**Published:** 2026-03-13

**Authors:** Guangli Deng, Wu Ruan, Qun Li, Qingyun Peng, Yunan Liu, Lingbin Lin, Yuan Li, Qianqian Shen, Yangrong Zhao, Junfeng Wang, Yi Chen, Ming-Wei Wang

**Affiliations:** 1 Research Center for Deepsea Bioresources, Sanya 572025, China; 2 State Key Laboratory of Chemical Biology, Shanghai Institute of Materia Medica, Chinese Academy of Sciences, Shanghai 201203, China; 3 South China Sea Institute of Oceanology, Chinese Academy of Sciences, Guangzhou 510301, China

**Keywords:** mycophenolic acid derivative, marine-derived *Penicillium*, acute myeloid leukemia, theoretical calculation, modified Mosher’s method

## Abstract

Nine new mycophenolic acid derivatives, penicacids O–W (**1**–**9**), two first-time reported natural products (**10**, **11**), and five known compounds (**12**–**16**), were isolated from a marine-derived fungus *Penicillium senticosum* RCDB005 found in a South China Sea sediment sample. Their structures were determined using NMR, HRESIMS, and optical rotatory dispersion (ORD) spectra, electronic circular dichroism (ECD) calculations, X-ray crystallography, and modified Mosher’s methods. Eight of these compounds were evaluated for anti-proliferative effects against nine human cancer cell lines and the IC_50_ values ranged from nM to μM levels. Compounds **5**, **7**–**9** showed potent inhibition activity against MOLM-13 acute myeloid leukemia cells with IC_50_ values between 0.13 and 1.13 μM.

## 1. Introduction

With great advantages of unique ecological environments, powerful gene clusters, and high yields of secondary metabolites, marine-derived fungi represent a gigantic and untapped reservoir for the exploration of novel bioactive marine natural products (MNPs). They offer immense potential in cancer treatment due to their diverse mechanisms of action, such as cytotoxic, antiproliferative, and immunomodulatory properties [1]. As a representative example, fucoxanthin (C_42_H_58_O_6_), found in brown algae such as *Undaria pinnatifida*, reduces leukemia and breast cancer cell growth [2]. Similarly, highly oxygenated polyketides, aspergilsmins A–G were isolated from *Aspergillus giganteus* NTU967 obtained from *Ulva lactuca*. Among them, aspergilsmin C and patulin exhibited significant inhibitory effects on prostate cancer PC-3 cells and human hepatocellular carcinoma SK-Hep-1 cells, with IC_50_ values ranging from 2.7 to 7.3 μM [3]. Importantly, marine-derived compounds have shown a broad spectrum of anticancer activities, including some that are resistant to conventional chemotherapy. These compounds offer a unique and largely untapped source of chemical diversity that can be exploited in the search for new and effective cancer treatments. This has led to a growing body of research focused on the discovery and development of marine-derived anticancer drugs, with many compounds undergoing preclinical and clinical evaluation [4].

One promising family of MNPs is mycophenolic acids (MPAs), mainly found in several *Penicillium* species [5,6]. MPA (**13**) is a potent inhibitor of human inosine 50-monophosphate dehydrogenase (IMPDH), a key enzyme in de novo biosynthesis of guanine nucleotides [7]. Since the discovery of MPA as a secondary fungal metabolite in 1893 [8], this compound and its derivatives have received widespread attention due to their broad bioactivities, such as immunosuppressive, antibacterial, antifungal, antiviral, and antitumor properties. Because of the potent bioactivity, many derivatives of MPA were investigated for drug development, and one derivative, mycophenolate mofetil (MMF), has been approved as an immunosuppressant and widely used in kidney, heart, and liver transplantation patients [9,10,11].

The aim of this study was to explore the secondary metabolites produced by *Penicillium senticosum* RCDB005 from a South China Sea sediment sample under laboratory conditions (solid-substrate fermentation cultures) in order to identify new bioactive substances.

## 2. Results and Discussion

**Figure 1:**
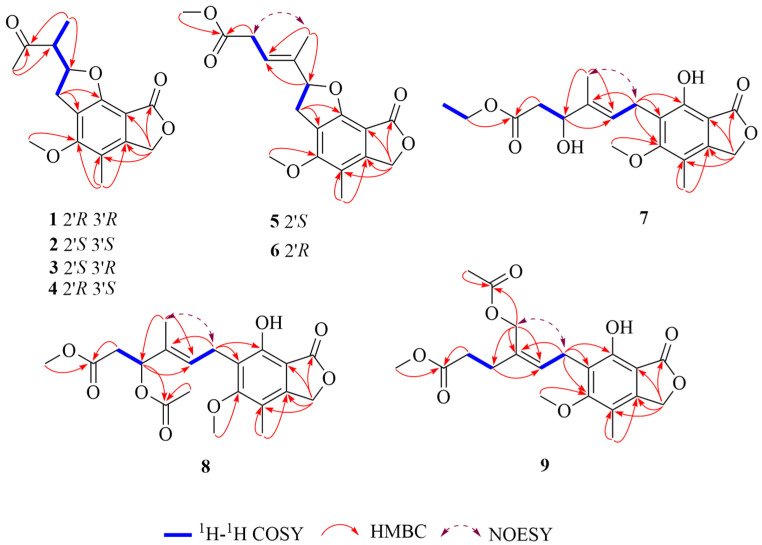
Key ^1^H-^1^H COSY, HMBC and NOESY correlations of compounds **1**–**9**.

**Figure 2:**
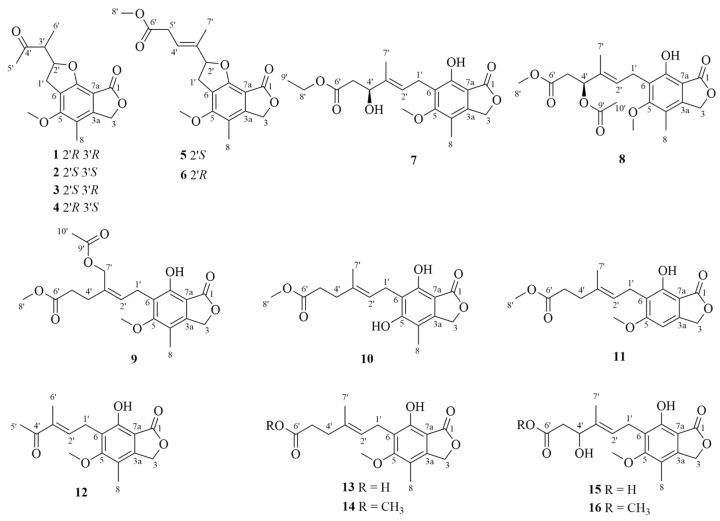
Structures of compounds **1**–**16**.

Compound **1** was isolated as a white powder. The molecular formula was deduced as C_16_H_18_O_5_ based on the (+)-HRESIMS ion at *m*/*z* 291.1224 [M+H]^+^ (calcd for C_16_H_19_O_5_, 291.1227), indicating eight degrees of unsaturation. The ^1^H, ^13^C NMR (Table 1), DEPT and HSQC spectra revealed the presence of two carbonyl carbons (*δ*_C_ 212.3, C-4′; 171.4, C-1), six aromatic quaternary carbons (*δ*_C_ 161.7, C-5; 158.7, C-7; 148.8, C-3a; 117.0, C-6; 116.3, C-4; 102.9, C-7a), one oxygen-bearing methine (*δ*_C/H_ 88.0/5.13, C-2′), one methine (*δ*_C/H_ 52.4/3.08, C-3′), one methylene group (*δ*_C/H_ 32.3/3.58, 3.21, C-1′), an oxygenated aliphatic methylene group (*δ*_C/H_ 70.7/5.22, C-3), one aromatic methyl group (*δ*_C/H_ 11.0/2.06, C-8), one aromatic methoxyl group (*δ*_C/H_ 59.7/4.00, 5-OCH_3_), and two aliphatic methyl groups (*δ*_C/H_ 29.8/2.27 C-5′; 12.1/1.16, C-6′). These data showed a close similarity to those reported for 2-(4-methoxy-5-methyl-8-oxo-2,3,6,8-tetrahydrobenzo [1,2-b:5,6-c′] difuran-2-yl) propanoic acid isolated from *Penicillium* sp. SCSIO sof101 [12], and then **1** was inferred to be an analog. Detailed analysis of 1D NMR (Table 1), HSQC, HMBC and ^1^H-^1^H COSY spectra (Figure 1) revealed that the major difference was the shift in the 4′-carbonyl carbon to the low field, suggesting that a methyl substitution may occur at the 4′-hydroxyl group, which was confirmed by the HMBC correlations from H-5′ to C-4′ and C-3′, from H-6′ to C-4′ and C-2′, from H-1′ to C-6 and C-7, from 5-OCH_3_ to C-5, from H-8 to C-3a, C-4, and C-5, from H-3 to C-1, C-3a, C-4 and C-7a. In addition, COSY correlations of H_2_-1′/H-2′/H-3′/H-6′ were observed, displaying the linkage between the carbons directly connected to the protons. Thus, the planar structure of **1** was established as shown (Figure 2) and named penicacid O.

**Figure 3:**
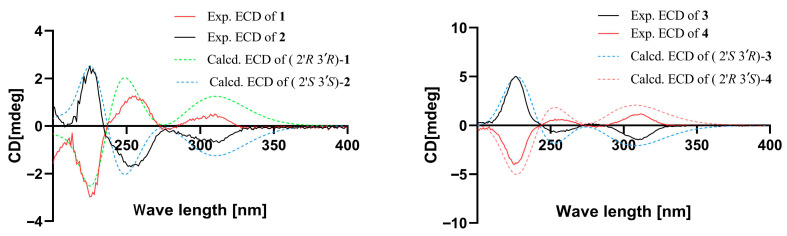
Experimental and calculated ECD curves of compounds **1**–**4**.

**Figure 4:**
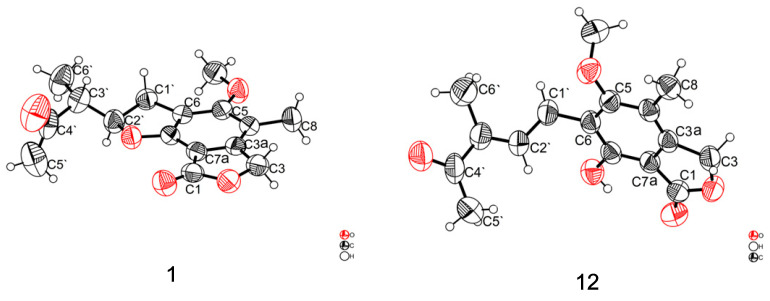
The diamond view of compounds **1** and **12**.

The theoretical ECD curve of **1** was then calculated and compared with the experimental ECD curve. The experimental ECD curve of **1** showed a higher similarity to the calculated ECD curve of (2′*R*, 3′*R*)-**1** (Figure 3). Finally, the absolute configuration of **1** was confirmed as 2′*R*, 3′*R* by X-ray crystallographic analysis (Figure 4), and the compound was named penicacid O (Figure 2).

Compound **2** was isolated as a white powder with the same molecular formula as that of **1** according to (+)-HRESIMS data, implying eight degrees of unsaturation. Their ^1^H, ^13^C NMR (Table 1) and DEPT data were quite similar, suggesting **2** may be an enantiomer of **1**. According to the 1D and 2D NMR data (Table 1 and Figure 1), the planar structure of **2** was established, and the compound was designated as penicacid P.

The theoretical ECD curve of **2** was then calculated and compared with the experimental ECD curve. The experimental ECD curve of **2** showed a higher similarity to the calculated ECD curve of (2′*S*, 3′*S*)-**2,** and the experimental ECD curve of **2** was completely opposite to the experimental ECD curve for **1** (Figure 3), suggesting that **2** was an enantiomer of **1**, which was further supported by the NOESY correlation between H-1′ and H-6′ in both **1** and **2** (Figure 1). Accordingly, the absolute configuration of **2** was identified as 2′*S*, 3′*S* (Figure 2).

Compound **3** was isolated as a white powder with the same molecular formula as that of **1** according to (+)-HRESIMS data, implying eight degrees of unsaturation. The ^1^H, ^13^C NMR (Table 1) and DEPT data of **3** closely resembled those of **1**. The major difference was that the chemical shift in methyl group (*δ*_C/H_ 12.7/1.28, recorded in CD_3_OD) attached to C-6′ in **3** was downfield shifted with respect to that in **1** (*δ*_C/H_ 12.1/1.16, recorded in CD_3_OD), suggesting **3** was a diastereomer at C-2′ or C-3′ of **4**. According to the 1D and 2D NMR data (Table 1 and Figure 1), the planar structure of **3** was established, and the compound was designated as penicacid Q.

The theoretical ECD curve of **3** was then calculated and compared with the experimental ECD curve. The experimental ECD curve of **3** showed a higher similarity to the calculated ECD curve of (2′*S*, 3′*R*)-**3** (Figure 3). Accordingly, the absolute configuration of **3** was identified as 2′*S*, 3′*R* (Figure 2).

Compound **4** was isolated as a white powder with the same molecular formula as that of **1** according to (+)-HRESIMS data, implying eight degrees of unsaturation. Their ^1^H, ^13^C NMR (Table 1) and DEPT data were quite similar, suggesting **4** may be an enantiomer of **3**. According to the 1D and 2D NMR data (Table 1 and Figure 1), the planar structure of **4** was established, and the compound was designated as penicacid R.

The theoretical ECD curve of **4** was then calculated and compared with the experimental ECD curve. The experimental ECD curve of **4** showed a higher similarity to the calculated ECD curve of (2′*R*, 3′*S*)-**4,** and the experimental ECD curve of **4** was completely opposite to the experimental ECD curve for **3** (Figure 3), suggesting **4** was an enantiomer of **3**. Accordingly, the absolute configuration of **4** was identified as 2′*R*, 3′*S* (Figure 2).

Compound **5** was obtained as yellow oil. The molecular formula was deduced as C_18_H_20_O_6_ based on the (+)-HRESIMS ion at *m*/*z* 333.1350 [M+H]^+^ (calcd for C_18_H_21_O_6_, 333.1333), indicating nine degrees of unsaturation. The ^1^H, ^13^C NMR (Table 2), DEPT and HSQC spectra revealed the presence of two ester carbonyls (*δ*_C_ 169.2, C-1; 172.0, C-6′), six aromatic quaternary carbons (*δ*_C_ 159.9, C-5; 158.1, C-7; 147.2, C-3a; 116.2, C-6; 114.9, C-4; 102.7, C-7a), one oxygenated sp^3^ methine (*δ*_C/H_ 90.1/5.40, C-2′), two methylene [including one oxygenated methylene (*δ*_C/H_ 69.2/5.13, C-3)] and four methyl groups [including two oxygenated methine groups (*δ*_C/H_ 59.3/3.95, 5-OCH_3_; 52.1/3.70, C-8′)]. These data showed a close similarity to those reported for compound **2** from *Penicillium* sp. HN 66 [13], and then **5** was inferred to be an analog. Detailed analysis of 1D NMR (Table 2), HSQC, HMBC and ^1^H-^1^H COSY spectra (Figure 1) revealed that the major difference was the shift of the C-4′ carbon to the low field and shift of the C-2′ carbon to the high field, suggesting the possibility that after opening the ∆^2′^ double bond on compound **16**, it reacted not only with the 4′-hydroxyl group to remove one molecule of H_2_O and form the ∆^3′^ double bond, but also with the 7-hydroxyl group to form a five-membered ring, as confirmed by the HMBC correlations from H-7′ to C-4′, C-3′ and C-2′, from H-1′ to C-6 and C-7, from H-8′ to C-6′, from H-5′ to C-6′, from 5-OCH_3_ to C-5, from H-8 to C-3a, C-4, and C-5, and from H-3 to C-1, C-3a, C-4 and C-7a. In addition, COSY correlations of H_2_-1′/H-2′ and H-4′/H_2_-5′ were observed, revealing the linkage between the carbons directly connected to the protons. Thus, the planar structure of **5** was established as shown (Figure 2) and named penicacid S. According to NOESY data correlation between H_2_-5′ and H_3_-7′, the double bond is in *E*-geometry (Figure 1).

Compound **6** was isolated as yellow oil with the same molecular formula as that of **5** according to (+)-HRESIMS data, implying nine degrees of unsaturation. Their ^1^H, ^13^C NMR (Table 2) and DEPT data were quite similar. According to the 1D and 2D NMR data (Table 2 and Figure 1), the planar structure of **6** was established, and the compound was designated as penicacid T. According to NOESY data (Figure 1), the double bond in **6** has an *E* configuration, which is confirmed by the correlation of H_2_-5′/H_3_-7′.

**Figure 5:**
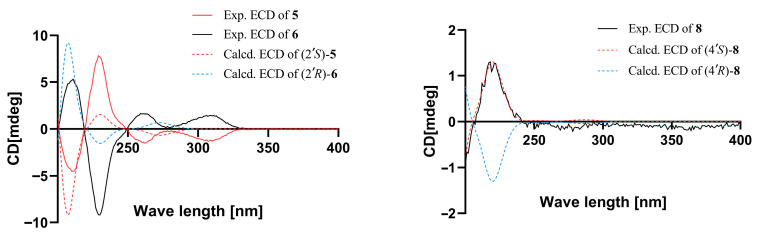
Experimental and calculated ECD curves of compounds **5, 6** and **8**.

The theoretical ECD curves of **5** and **6** were then calculated and compared with the experimental ECD curves. Compound **5** showed a higher similarity to the calculated ECD curve for 2′*S*. Accordingly, the absolute configuration of **5** was identified as 2′*S* (Figure 5). And the experimental ECD curve of **6** was completely opposite to the experimental ECD curve for **5** (Figure 5). Accordingly, the absolute configuration of **6** was identified as 2′*R* (Figure 5).

Compound **7** was obtained as yellow oil. The molecular formula was deduced as C_19_H_24_O_7_ based on the (+)-HRESIMS ion at *m*/*z* 387.1414 [M+Na]^+^ (calcd for C_19_H_24_O_7_Na, 387.1414), indicating eight degrees of unsaturation. Careful comparison of the 1D NMR data of **7** and penicacid N revealed a high structural similarity except for the appearance of an oxyethyl group (*δ*_C/H_ 60.9/4.13, C-8′; 14.3/1.24, C-9′) in **7** instead of the methoxy group at C-6′ in penicacid N [13]. ^1^H-^1^H COSY correlations of H_2_-8′/H-9′ were observed, revealing the linkage between the carbons directly connected to the protons. The location of the oxyethyl group at C-6′ in **7** was confirmed by the HMBC correlation from H-8′ to C-6′ (*δ*_C_ 172.8). Additionally, the key HMBC correlations from H-5′ to C-6′, from H-4′ to C-2′, from H-7′ to C-2′ and C-4′, from H-1′ to C-3′, C-5, C-6 and C-7, from 5-OCH_3_ to C-5, from H-8 to C-3a and C-4, and from H-3 to C-1, C-3a, C-4 and C-7a further supported the planar structure of **7** (Figure 2), which was elucidated as 4′*S*-hydroxy-6′-ethoxy mycophenolic acid and named penicacid U. According to NOESY data correlation between H_2_-1′ and H_3_-7′, the double bond is in *E*-geometry (Figure 1).

**Figure 6:**
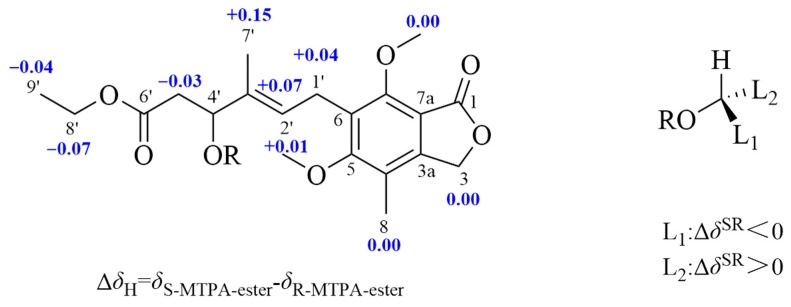
Δ*δ*_H(*S*–*R*)_ (in ppm) values for the MTPA esters of compound **7**.

The configuration at C-4′ was determined by a modified Mosher’s method [14,15,16]. Firstly, the phenol group of compound **7** was methylated by TMSCHN_2_ [7]. Then, the (*S*)- and (*R*)-MTPA esters of **7**, **7a** and **7b** were obtained by acylation of **7** with (*R*)- and (*S*)-MTPA-Cl, respectively. According to the rule of the modified Mosher’s method, a 4′*S* configuration in **7** was inferred from the Δ*δ*_H_ values (Δ*δ*_H_ = *δ_S_*_-MTPA-ester_ − *δ_R_*_-MTPA-ester_) of the hydrogen signals adjacent to C-4′ (Figure 6). Thus, the absolute configuration of **7** was established as 4′*S* (Figure 2).

Compound **8** was obtained as yellow oil. The molecular formula was deduced as C_20_H_24_O_8_ based on the (+)-HRESIMS ion at *m*/*z* 415.1373[M+Na]^+^ (calcd for C_20_H_24_O_8_Na, 415.1363), indicating nine degrees of unsaturation. After comparing its NMR data with those of penicacid N [13], compound **8** was determined to have an identical planar structure to penicacid N. However, the optical rotatory dispersion (ORD) value of compound **8** was −10°, which was opposite to the ORD value of penicacid N ${\left[\alpha \right]}_{D}^{25}$ = +8 (*c* 0.1, MeOH). This discrepancy suggests that the configuration of the C-4′ position in compound **8** is opposite to that of penicacid N. The ECD spectrum of **8** (Figure 5) shows a negative Cotton effect at 205 nm, as opposed to penicacid N. The absolute configuration of chiral center 4′*S* was determined by comparing the experimental and calculated ECD spectra (Figure 5). According to NOESY data correlation between H_2_-1′ and H_3_-7′, the double bond is in *E*-geometry (Figure 1). Consequently, the structure of **8** was elucidated as 4′*S*-methyl acetate-6′-methoxy mycophenolic acid and named penicacid V.

Compound **9** was obtained as yellow oil. The molecular formula was deduced as C_20_H_24_O_8_ based on the (+)-HRESIMS ion at *m*/*z* 415.1373[M+Na]^+^ (calcd for C_20_H_24_O_8_Na, 415.1363), indicating nine degrees of unsaturation. This suggests that compound **9** should have an identical chemical formula as compound **8**. The most notable difference observed in the NMR data (1D NMR in Table 3; HSQC, HMBC, and ^1^H-^1^H COSY in Figure 1) was an upfield shift for C-4′ and a downfield shift for C-7′. This shift pattern implies that the methyl acetate group in compound **8** may have been repositioned as confirmed by the HMBC correlations from H-7′ to C-9′, C-4′, C-3′ and C-2′. Additionally, the key HMBC correlations from H-10′ to C-9′, from H-8′ to C-6′, from H-5′ to C-6′, from H-4′ to C-2′, from H-1′ to C-3′, C-5, C-6 and C-7, from 5-OCH_3_ to C-5, from H-8 to C-3a and C-4, and from H-3 to C-1, C-3a, C-4 and C-7a further supported the planar structure of **9**. In addition, the COSY correlations of H_2_-1′/H-2′ and H_2_-4′/H_2_-5′ were observed, revealing the linkage between the carbons directly connected to the protons. Thus, the planar structure of **9** was established as shown (Figure 2), elucidated as 6′-methoxy-7′-methyl acetate mycophenolic acid and named penicacid W. According to NOESY data correlation between H_2_-1′ and H_2_-7′, the double bond is in *Z*-geometry (Figure 1).

Compound **10** was obtained as yellow oil and determined to be C_17_H_20_O_6_ based on the (-)-HRESIMS. The planar structure of **10** was the same as a synthesized compound methyl-6-(4,6-dihydroxy-7-methyl-3-oxo-1,3-dihydroisobenzofuran-5-yl)-4-methylhex-4-enoate (**10**) by 1D and 2D NMR spectra (Table S1 and Figure S1) [17]. The double bond of **10** was identified as in *E*-geometry by NOESY data correlation between H_2_-1′ and H_3_-7′ (Figure S2). Notably, compound **10** was isolated from a natural source for the first time.

Compound **11** was isolated as yellow oil, and the molecular formula was determined as C_17_H_20_O_6_ by (-)-HRESIMS. The 1D and 2D NMR data (Table S1 and Figure S1) showed the same planar structure as a synthesized compound methyl (*E*)-6-(4-hydroxy-6-methoxy-3-oxo-1,3-dihydroisobenzofuran-5-yl)-4-methylhex-4-enoate [18]. Compound **11** was often synthesized but has never been isolated from natural sources. According to NOESY data correlation between H_2_-1′ and H_3_-7′, the double bond is in *E*-geometry (Figure S3).

Five known compounds were compared of their spectrometric data with those in the literature and identified as penicacid L (**12**) [13], mycophenolic acid (**13**) [19], 4′-hydroxy-MPA (**14**) [20], methyl mycophenolic acid (**15**) [21], and penicacid M (**16**) [13]. Among them, the double bond of **12** is in *E*-geometry by X-ray crystallographic analysis (Figure 3).

All the isolated compounds were evaluated for their anti-proliferative effects against human acute myeloid leukemia cells (OCI-AML3) using the CCK-8 method. Among them, compounds **5**, **7**–**9** and **13**–**16** showed potent inhibitory activity, while the remaining eight compounds had IC_50_ values above 40 μM (Table S2). Compounds **5**, **7**–**9** and **13**–**16** were subsequently assessed against a panel of human tumor cell lines derived from leukemia, lymphoma, colon cancer, and pancreatic cancer. As shown in Table 4, all of them displayed a broad spectrum of anti-proliferative effects with IC_50_ values ranging from 60 nM to more than 40 μM, but the inhibition was weaker than that of commonly used chemotherapeutic agent doxorubicin. MOLM-13 (human acute myeloid leukemia), MOLT-3 (human T cell leukemia), and OCI-AML3 (human acute myeloid leukemia) cells were particularly sensitive to these compounds, with **5**, **7** and **8** exhibiting stronger inhibition than MPA (**13**) in MOLM-13 cells. In contrast, the human pancreatic cell line BXPC3 showed the lowest sensitivity. Compound **9** had the weakest anti-proliferative effect against tumor cells, with IC_50_ values above 40 μM in four out of eight cell lines.

## 3. Materials and Methods

### 3.1. General Experiment Procedures

Optical rotations were taken on a Rudolph Research Analytical Autopol III polarimeter (Rudolph Research Analytical, Hackettstown, NJ, USA). The UV spectra were recorded on an Evolution 350 UV–Vis spectrometer (ThermoFisher Scientific, Madison, WI, USA). ECD data were measured on a Chirascan V100 spectrometer (Applied Photophysics, Leatherhead, Surrey, UK). The NMR spectra were recorded on Bruker 600 MHz NMR spectrometers (Bruker AG, Zürich, Switzerland), while HRESIMS data were obtained using Agilent 6520 and 6545 Q-TOF LC-MS spectrometers (Agilent Technologies, Singapore). Crystal data were obtained on a Bruker D8 Single-Crystal X-Ray Diffractometer (Bruker AXS, Karlsruhe, Germany). The separation and purification of the isolated compounds were carried out using the Agilent 1260 and 1290 HPLC (Agilent Technologies, Waldbronn, Germany) equipped with a 250 mm × 10 mm i.d., 5 μm, Titank C18 column (FLM, Guangzhou, China). The other chromatographic columns used included a 250 mm × 4.6 mm i.d., 5 μm, Chiral NQ(2)-RH column (FLM) and 250 mm × 10 mm i.d., Pursuit 5 PFP column (Agilent, Santa Clara, CA, USA). Medium pressure liquid chromatography (MPLC) separations were performed on a Buchi Sepacore^®^ X50 (BUCHI Labortechnik AG, Flawil, Switzerland) using a C18 column (SW-5222-120-SP, Santai Technologies, Changzhou, China) and glass columns filled with 300–400 mesh silica gel (Qingdao Marine Chemical Factory, Qingdao, China). The solvents and culture media utilized were sourced from Sinopharm Chemical Reagent Co., Ltd. (Shanghai, China), while the deuterated solvents were obtained from Cambridge Isotope Laboratories, Inc. (Shanghai, China). Deuterated solvents used for NMR were CDCl_3_ (*δ*_H_ 7.260/*δ*_C_ 77.160) and CD_3_OD (*δ*_H_ 3.310/*δ*_C_ 49.000). (Trimethylsilyl)diazomethane was procured from Shanghai McLean Biochemical Technology Co., Ltd. (Shanghai, China). (*R*)- and (*S*)-MTPA-Cl ((*S*)-(+)-*a*-methoxy-*a*-(trifluoromethyl)phenylacetyl chloride) were procured from Shanghai Aladdin Biochemical Technology Co., Ltd. (Shanghai, China).

### 3.2. Microbial Materials and Fermentation

The fungal strain RCDB005 was isolated from the sediments collected at a depth of 1400 m in the South China Sea and identified as *Penicillium senticosum* (accession No. PX069401) based on ITS region sequences. Strain RCDB005 was cultured on MB (malt extract powder 15 g, sea salt 15 g, H_2_O 1 L, 15 g agar, pH 7.4–7.8) agar plates for 3 days before inoculation into MB seed liquid (without agar), which was incubated at 28 °C on a rotary shaker at 180 rpm for 2 days. The seed liquid was then transferred to sterile rice medium (rice 200 g, 1.5% sea salt water 200 mL, per bottle), and large-scale fermentation was performed in 100 flasks at 25 °C for 31 days.

### 3.3. Extraction and Isolation

The rice culture of RCDB005 was broken by ultrasonication and transferred to a vat, soaked in 60 L of EtOAc overnight, the extraction was repeated three times, and the solvent was evaporated to obtain a crude (207.8 g). The crude was filtered to remove spores and insoluble matter and extracted with MeOH and PE three times to obtain the de-oiled methanolic fraction (122.3 g). The methanolic fraction was first extracted with DCM–PE (1:9, 3:7, 5:5, 7:3, 9:1, 1:0, *v*/*v*, per 3.0 L) and DCM–MeOH (1:9, 3:7, 5:5, 7:3, 1:0, *v*/*v*, per 6.0 L). Gradient elution of the de-oiled crude was subjected to silica gel column chromatography, and five fractions (Fr. 1–Fr. 5) were obtained. Fraction Fr. 1 (20.5 g) was subjected to MPLC with MeOH and H_2_O (2:8–1:0, *v*/*v*, per 2.0 L). Subfraction Fr. 1-1 (633.0 mg) was isolated and purified by preparative HPLC with a Titank C18 column (55% ACN in H_2_O, *v*/*v*, 2 mL/min) to give compound **12** (3.1 mg, *t*_R_ = 31 min) and mixture S7. Mixture S7 (30.0 mg) was isolated and purified by analytical HPLC with a chiral column (70% MeOH in H_2_O, *v*/*v*, 0.8 mL/min) to give compounds **1** (2.3 mg, *t*_R_ = 33 min), **2** (2.0 mg, *t*_R_ = 20 min), **3** (1.0 mg, *t*_R_ = 16 min), and **4** (2.6 mg, *t*_R_ = 27 min). Fraction Fr. 2 (3.0 g) was isolated and purified by preparative HPLC with a Pursuit 5 PFP column (35% ACN in H_2_O, *v*/*v*, 2 mL/min) to give compound **15** (11.5 mg, *t*_R_ = 12 min). Fraction Fr. 3 (40.6 g) was subjected to MPLC with MeOH and H_2_O (2:8–1:0, *v*/*v*, per 2.0 L) to obtain the compound **16** (908.6 mg) and nine subfractions. Subfraction Fr. 3–7 was isolated by preparative HPLC with a Titank C18 column (50% ACN in H_2_O, *v*/*v*, 2 mL/min) to give compound **7** (12.4 mg, *t*_R_ = 16 min), compound **8** (10.2 mg, *t*_R_ = 22 min), compound **13** (11.7 mg, *t*_R_ = 14 min), compound **14** (9.9 mg, *t*_R_ = 27 min), compound **11** (4.6 mg, *t*_R_ = 17 min), compound **10** (3.4 mg, *t*_R_ = 24 min) and mixture S15. Mixture S15 (15.5 mg) was isolated and purified by analytical HPLC with a chiral column (50% ACN in H_2_O, *v*/*v*, 0.8 mL/min) to give compounds **5** (1.0 mg, *t*_R_ = 20 min), **6** (1.0 mg, *t*_R_ = 22 min) and **3** (0.9 mg, *t*_R_ = 24 min).

Penicacid O (**1**). White powder; UV (MeOH) λ_max_ (log *ε*) 222 (4.54), 252 (4.08), 307 (3.82) nm; ECD (0.15 mg/mL, MeOH) λ_max_ (Δ*ε*) 213 (−0.17), 225 (−1.74), 256 (0.74), 279 (−0.09), 310 (0.29) nm; ^1^H and ^13^C NMR data, see Table 1; HRESIMS *m*/*z* 291.1224 [M + H]^+^ (calcd for C_16_H_19_O_5_, 291.1227).

Penicacid P (**2**). White powder; UV (MeOH) λ_max_ (log *ε*) 222 (4.13), 252 (3.66), 307 (3.41) nm; ECD (0.30 mg/mL, MeOH) λ_max_ (Δ*ε*) 208 (−0.01), 225 (0.74), 253 (−0.50), 276 (−0.04), 313 (−0.20) nm; ^1^H and ^13^C NMR data, see Table 1; HRESIMS *m*/*z* 291.1227 [M + H]^+^ (calcd for C_16_H_19_O_5_, 291.1227).

Penicacid Q (**3**). White powder; UV (MeOH) λ_max_ (log *ε*) 219 (4.15), 306 (3.38) nm; ECD (0.30 mg/mL, MeOH) λ_max_ (Δ*ε*) 226 (1.47), 254 (−0.21), 279 (0.06), 310 (−0.43) nm; ^1^H and ^13^C NMR data, see Table 1; HRESIMS *m*/*z* 291.1227 [M+H]^+^ (calcd for C_16_H_19_O_5_, 291.1227).

Penicacid R (**4**). White powder; UV (MeOH) λ_max_ (log *ε*) 221 (4.42), 306 (3.69) nm; ECD (0.30 mg/mL, MeOH) λ_max_ (Δ*ε*) 225 (−1.18), 253 (0.19), 281 (−0.06), 312 (0.34) nm; ^1^H and ^13^C NMR data, see Table 1; HRESIMS *m*/*z* 291.1224 [M+H]^+^ (calcd for C_16_H_19_O_5_, 291.1227).

Penicacid S (**5**). Yellow oil; UV (MeOH) λ_max_ (log *ε*) 223 (4.45), 256 (3.99), 308 (3.75) nm; ECD (0.20 mg/mL, MeOH) λ_max_ (Δ*ε*) 210 (−2.28), 229 (3.93), 262 (−0.76), 278 (−0.10), 307 (−0.65) nm; ^1^H and ^13^C NMR data, see Table 2; HRESIMS *m*/*z* 333.1350 [M + H]^+^ (calcd for C_18_H_21_O_6_, 333.1333).

Penicacid T (**6**). Yellow oil; UV (MeOH) λ_max_ (log *ε*) 222 (4.43), 254 (3.95), 308 (3.72) nm; ECD (0.20 mg/mL, MeOH) λ_max_ (Δ*ε*) 211 (2.68), 230 (−4.63), 259 (0.83), 277 (0.07), 306 (0.73) nm; ^1^H and ^13^C NMR data, see Table 2; HRESIMS *m*/*z* 333.1347 [M + H]^+^ (calcd for C_18_H_21_O_6_, 333.1333).

Penicacid U (**7**). Yellow oil; ${\left[\alpha \right]}_{D}^{25}$ = −2.3 (*c* 0.3, MeOH); UV (MeOH) λ_max_ (log *ε*) 215 (4.51), 250 (3.85), 304 (3.57) nm; ECD (0.10 mg/mL, MeOH) λ_max_ (Δ*ε*) 210 (0.58), 222 (1.21), 281 (−0.20) nm; ^1^H and ^13^C NMR data, see Table 3; HRESIMS *m*/*z* 387.1414 [M + Na]^+^ (calcd for C_19_H_24_O_7_Na, 387.1414).

Penicacid V (**8**). Yellow oil; ${\left[\alpha \right]}_{D}^{25}$ = −10.3 (*c* 0.3, MeOH); UV (MeOH) λ_max_ (log *ε*) 216 (4.57), 250 (3.89), 304 (3.62) nm; ECD (0.10 mg/mL, MeOH) λ_max_ (Δ*ε*) 218 (1.55), 278 (−0.25) nm; ^1^H and ^13^C NMR data, see Table 3; HRESIMS *m*/*z* 415.1373 [M + Na]^+^ (calcd for C_20_H_24_O_8_Na, 415.1363).

Penicacid W (**9**). Yellow oil; UV (MeOH) λ_max_ (log *ε*) 216 (4.55), 250 (3.91), 305 (3.60) nm; ECD (0.20 mg/mL, MeOH) λ_max_ (Δ*ε*) 211 (0.16), 229 (−0.23), 257 (0.01) nm; ^1^H and ^13^C NMR data, see Table 3; HRESIMS *m*/*z* 415.1377 [M+Na]^+^ (calcd for C_20_H_24_O_8_Na, 415.1363).

### 3.4. X-Ray Crystallographic Analysis

Colorless crystals of compound **1** were obtained in PE/DCM (1:1) solution by slow evaporation at 4 °C. Similarly, colorless crystals of compound **12** were obtained in MeOH/PE/H_2_O (5:5:1) solution by slow evaporation at 4 °C. Their crystal data were collected from a single crystal on a Bruker D8 VENTURE dual-wavelength Mo/Cu three-circle diffractometer with a microfocus sealed X-ray tube using mirror optics as the monochromator and a Bruker PHOTON III detector. The crystallographic data of **1** and **12** were measured at 302.00 K, respectively, with Cu*K_α_* radiation (λ = 1.54178 Å). The structure was solved by the direct method using SHELXT and refined by the full-matrix least-square approach against *F*^2^ by SHELXL-2019/1 [22,23]. All non-hydrogen atoms were refined with anisotropic displacement parameters. All C-bound hydrogen atoms were refined with isotropic displacement parameters. Crystallographic data of **1** and **12** (Tables S4 and S5) have been deposited in the Cambridge Crystallographic Data Centre (deposition No.: CCDC 2498064 for **1** and CCDC 2498063 for **12**). These data can be obtained, free of charge, on application to CCDC, 12 Union Road, Cambridge CB21EZ, UK [fax: +44(0)-1223-336033 or e-mail: deposit@ccdc.cam.ac.uk].

Crystal data for **1**. C_16_H_18_O_5_, *Mr* = 290.30, crystal size 0.228 × 0.142 × 0.015 mm^3^, monoclinic, *a* = 5.02400(10) Å, *b* = 13.6427(4) Å, *c* = 20.9641(6) Å, *α* = 90°, *β* = 96.049(2)°, *γ* = 90°, *V* = 1428.90(7) Å^3^, T = 302.00 K, space group *P*2_1_, *Z* = 4, μ(Cu Kα) = 0.831 mm^−1^, 27,611 reflections collected, 4888 independent reflections (*R*_int_ = 0.0635, *R*_sigma_ = 0.0494). The final *R*_1_ values were 0.0466 (*I* ≥ 2σ(*I*)). The final w*R*_2_ values were 0.1347 (*I* ≥ 2σ(*I*)). The final *R*_1_ values were 0.0798 (all data). The final w*R*_2_ values were 0.1347 (all data). The goodness of fit on *F*^2^ was 1.005. The Flack parameter was −0.1(2) (Table S3).

Crystal data for **12**. C_16_H_18_O_5_, *Mr* = 290.30, crystal size 0.306 × 0.02 × 0.004 mm^3^, monoclinic, *a* = 4.5267(3) Å, *b* = 11.7847(7) Å, *c* = 14.4718(9) Å, *α* = 71.277(4)°, *β* = 86.160(4)°, *γ* = 85.886(4)°, *V* = 728.48(8) Å^3^, T = 302.00 K, space group $P\overset{-}{1}$, *Z* = 2, μ(Cu Kα) = 0.815 mm^−1^, 31,113 reflections collected, 2584 independent reflections (*R*_int_ = 0.0517, *R*_sigma_ = 0.0229). The final *R*_1_ values were 0.0557 (*I* ≥ 2σ(*I*)). The final w*R*_2_ values were 0.1615 (*I* ≥ 2σ(*I*)). The final *R*_1_ values were 0.0709 (all data). The final w*R*_2_ values were 0.1748 (all data). The goodness of fit on *F*^2^ was 1.093 (Table S4).

### 3.5. ECD Calculation

Conformational searches were carried out by means of the Maestro 12.8 software using the Molecular Merck force field. All density functional theory and time-dependent calculations were performed with the Gaussian 09 program. Conformers within a 12 kcal/mol energy window were generated and optimized by DFT calculations at the B3LYP/6-31+G (d,p) level [24].

The relative configurations of compounds **1**–**6** and **8** also underwent a series of random conformational searches using Maestro 12.8 software with MMFF. Subsequently, the low-energy conformers were re-optimized using the TD-DFT method at the B3LYP/6-31G (d,p) level in MeOH, employing the IEFPCM model in the Gaussian 09 program. Theoretical calculations for ECD were performed in MeOH at the B3LYP/6-31G (d,p) level [25]. The calculated ECD curves were generated using the SpecDis 3.0 and GraphPad Prism 8.0 from dipole length rotational strengths by applying Gaussian band shapes with a half-bandwidth ranging from 0.2 to 0.4 eV, based on the contributions of each conformer calculated via the Boltzmann distribution following UV correction.

### 3.6. Methylate the Phenol Group of 7 (7-R)

To a solution of **7** (10.0 mg) in CH_2_Cl_2_ (3.0 mL), excess TMSCHN_2_ (2 mL, 2.0 M) was added, and the mixture was stirred at room temperature (RT) for 48 h. The reaction mixture was concentrated under vacuum and purified by analytical HPLC with a Pursuit 5 PFP column (40% ACN in H_2_O, *v*/*v*, 2 mL/min) to furnish 3.2 mg of **7R**: HRESIMS *m*/*z* 401.1583 [M+Na]^+^ (calcd for C_20_H_26_O_7_Na, 401.1571).

### 3.7. Preparation of the (S)- and (R)-MTPA Esters of 7R

Compound **7R** (3.2 mg), 4-(dimethylamino)pyridine (1.0 mg) and (*R*)-MTPA-Cl were added to 400 µL stirred pyridine solution. The mixture was reacted at RT for 48 h and then dried to give (*S*)-MTPA ester (**7Ra**). (*R*)-MTPA ester of **7R** (**7Rb**) was obtained by the same experimental procedure.

(*S*)-MTPA ester of **7R** (**7Ra**). ^1^H NMR (600 MHz, CDCl_3_) *δ*_H_ 5.15 (2H, s, H_2_-3), 2.18 (3H, s, H_3_-8), 3.42 (2H, dd, *J* = 11.2, 7.1 Hz, H_2_-1′), 5.68 (1H, t, *J* = 6.9 Hz, H-2′), 2.82–2.54 (2H, m, H_2_-5′), 1.84 (3H, s, H_3_-7′), 4.02 (2H, qd, *J* = 7.2, 6.1 Hz, H_2_-8′), 1.16 (3H, t, *J* = 7.1 Hz, H_3_-9′), 4.02 (3H, s, H_3_-5-OCH_3_), 3.74 (3H, s, H_3_-7-OCH_3_); HRESIMS *m*/*z* 617.1979 [M + Na]^+^ (calcd for C_30_H_33_F_3_O_9_Na, 617.1969).

(*R*)-MTPA ester of **7R** (**7Rb**). ^1^H NMR (600 MHz, CDCl_3_) *δ*_H_ 5.15 (2H, s, H_2_-3), 2.18 (3H, s, H_3_-8), 3.38 (2H, dd, *J* = 11.2, 7.1 Hz, H_2_-1′), 5.61 (1H, t, *J* = 6.8 Hz, H-2′), 2.85–2.54 (2H, m, H_2_-5′), 1.69 (3H, s, H_3_-7′), 4.09 (2H, qd, *J* = 7.1, 1.8 Hz, H_2_-8′), 1.20 (3H, t, *J* = 7.1 Hz, H_3_-9′), 4.01 (3H, s, H_3_-5-OCH_3_), 3.74 (3H, s, H_3_-7-OCH_3_); HRESIMS *m*/*z* 617.1986 [M + Na]^+^ (calcd for C_30_H_33_F_3_O_9_Na, 617.1969).

### 3.8. Biological Assay

The bioactivity of the 16 compounds and doxorubicin (MedChemExpress, Monmouth Junction, NJ, USA) against human tumor cell proliferation was assessed by the CCK-8 method, a colorimetric assay for measuring cell viability, proliferation, and cytotoxicity [26]. MOLT-3, CCRF-CEM, HT-29 and BXPC-3 were obtained from ATCC; Karpas-422 and WSU-DL-CL2 were purchased from DSMZ; MOLM-13 was obtained from JRCB; OCI-AML3 was purchased from COBIOER (Nanjing, China); and HL-60 was obtained from the National Collection of Authenticated Cell Cultures (Shanghai, China). All cells were cultured according to the recommendations. Cells were seeded in each well of 96-well plates and cultured with different concentrations of test compounds for 72 h. Then, 10 μL of CCK-8 was added per well. The absorbance of each well was measured after 2 h of incubation at 37 °C by SpectraMAX190 (Molecular Devices, San Jose, CA, USA) at 450 nm. The IC_50_ value was calculated by SoftMax Pro (Molecular Devices).

## 4. Conclusions

In summary, *Penicillium senticosum* RCDB005 was cultured in a solid medium and extracted with ethyl acetate (EtOAc). The resulting crude extracts were fractionated using a silica gel column and subsequently subjected to high-performance liquid chromatography (HPLC) to afford sixteen compounds including nine new MPA derivatives (**1**–**9**), two new natural products (**10**, **11**), and five known compounds (**12**–**16**) (Figure 2). Their structures were determined using NMR, HRESIMS and optical rotatory dispersion (ORD) spectra, electronic circular dichroism (ECD) calculations, X-ray crystallography and modified Mosher’s methods. Eight of these compounds were evaluated for anti-proliferative effects against nine human cancer cell lines, and the IC_50_ values ranged from nM to μM levels. Compounds **5**, **7**–**9** showed potent inhibitory activity against human acute myeloid leukemia cells (MOLM-13), with IC_50_ values between 0.13 and 1.13 μM. This study enriched our knowledge of mycophenolic acid derivatives isolated from marine microorganisms. However, given the insufficient sample amount and the weak potency of these compounds, mechanistic studies were not performed. Our future work will focus on identifying more potent analogs to enable subsequent in-depth studies.

## Figures and Tables

**Table 1:** ^1^H NMR (600 MHz) and ^13^C NMR (150 MHz) data for compounds **1**–**4** in CD_3_OD.

	1		2		3		4	
Position Ion	*δ*_C_, Type	*δ*_H_ (*J* in Hz)	*δ*_C_, Type	*δ*_H_ (*J* in Hz)	*δ*_C_, Type	*δ*_H_ (*J* in Hz)	*δ*_C_, Type	*δ*_H_ (*J* in Hz)
1	171.4, C		171.4, C		171.5, C		171.5, C	
3	70.7, CH_2_	5.22, s	70.7, CH_2_	5.22, s	70.7, CH_2_	5.22, s	70.7, CH_2_	5.22, s
3a	148.8, C		148.8, C		148.8, C		148.8, C	
4	116.3, C		116.3, C		116.2, C		116.2, C	
5	161.7, C		161.7, C		161.7, C		161.7, C	
5-OCH_3_	59.7, CH_3_	4.00, s	59.7, CH_3_	4.00, s	59.7, CH_3_	3.98, s	59.7, CH_3_	3.98, s
6	117.0, C		117.0, C		117.2, C		117.2, C	
7	158.7, C		158.7, C		158.9, C		158.9, C	
7a	102.9, C		102.9, C		102.8, C		102.8, C	
8	11.0, CH_3_	2.06, s	11.0, CH_3_	2.07, s	11.0, CH_3_	2.06, s	11.0, CH_3_	2.06, s
1′	32.3, CH_2_	3.58, dd (15.4, 9.3); 3.21, dd (15.5, 9.3)	32.3, CH_2_	3.58, dd (15.5, 9.2); 3.20, dd (15.5, 9.2)	32.9, CH_2_	3.59, dd (15.5, 9.4); 3.19, dd (15.5, 9.4)	32.9, CH_2_	3.59, dd (15.6, 9.4); 3.19, dd (15.6, 9.4)
2′	88.0, CH	5.13, q (7.9)	88.0, CH	5.13, q (7.9)	87.7, CH	5.16, q (7.2)	87.7, CH	5.16, q (7.2)
3′	52.4, CH	3.08, p (7.2)	52.4, CH	3.08, p (7.2)	52.7, CH	3.08, p (7.0)	52.7, CH	3.07, p (7.0)
4′	212.3, C		212.3, C		212.2, C		212.2, C	
5′	29.8, CH_3_	2.27, s	29.8, CH_3_	2.27, s	29.6, CH_3_	2.23, s	29.6, CH_3_	2.23, s
6′	12.1, CH_3_	1.16, d (7.0)	12.1, CH_3_	1.16, d (7.0)	12.7, CH_3_	1.28, d (7.1)	12.7, CH_3_	1.28, d (7.1)

**Table 2:** ^1^H NMR (600 MHz) and ^13^C NMR (150 MHz) data for compounds **5** and **6** in CDCl_3_.

	5		6	
Position	*δ*_C_, Type	*δ*_H_ (*J* in Hz)	*δ*_C_, Type	*δ*_H_ (*J* in Hz)
1	169.2, C		169.2, C	
2				
3	69.2, CH_2_	5.13, s	69.2, CH_2_	5.13, s
3a	147.2, C		147.2, C	
4	114.9, C		114.9, C	
5	159.9, C		159.9, C	
5-OCH_3_	59.3, CH_3_	3.95, s	59.3, CH_3_	3.95, s
6	116.2, C		116.2, C	
7	158.1, C		158.1, C	
7a	102.7, C		102.7, C	
8	11.2, CH_3_	2.06, s	11.2, CH_3_	2.06, s
1′	33.1, CH_2_	3.52, dd (15.5, 8.1); 3.20, dd (15.5, 8.1)	33.1, CH_2_	3.52, dd (15.5, 8.0); 3.20, dd (15.5, 8.0)
2′	90.1, CH	5.40, t (8.1)	90.1, CH	5.40, t (8.1)
3′	136.5, C		136.5, C	
4′	120.8, CH	5.79, t (7.1)	120.8, CH	5.79, t (7.1)
5′	33.3, CH_2_	3.12, d (7.1)	33.3, CH_2_	3.12, d (7.1)
6′	172.0, C		172.0, C	
7′	11.4, CH_3_	1.68, s	11.4, CH_3_	1.68, s
8′	52.1, CH_3_	3.7, s	52.1, CH_3_	3.7, s

**Table 3:** ^1^H NMR (600 MHz) and ^13^C NMR (150 MHz) data for compounds **8** in CD_3_OD, **7** and **9** in CDCl_3_.

	7		8		9	
Position	*δ*_C_, Type	*δ*_H_ (*J* in Hz)	*δ*_C_, Type	*δ*_H_ (*J* in Hz)	*δ*_C_, Type	*δ*_H_ (*J* in Hz)
1	173.0, C		173.7, C		172.9, C	
3	70.2, CH_2_	5.19, s	70.8, CH_2_	5.25, s	70.2, CH_2_	5.20, s
3a	144.3, C		147.0, C		144.6, C	
4	116.9, C		117.9, C		117.0, C	
5	163.9, C		164.9, C		163.8, C	
5-OCH_3_	61.2, CH_3_	3.76, s	61.6, CH_3_	3.76, s	61.3, CH_3_	3.77, s
6	121.7, C		122.7, C		121.1, C	
7	153.8, C		154.7, C		153.7, C	
7a	106.5, C		107.8, C		106.6, C	
8	11.7, CH_3_	2.14, s	11.4, CH_3_	1.98, s	11.7, CH_3_	2.15, s
1′	22.4, CH_2_	3.42, dd (7.2, 3.1)	23.2, CH_2_	3.42, d (7.1)	22.6, CH_2_	3.49, d (7.1)
2′	124.2, CH	5.55, t (7.1)	128.2, CH	5.56, t (6.5)	128.8, CH	5.51, t (7.4)
3′	136.1, C		133.6, C		133.0, C	
4′	73.3, CH	4.42, dd (9.3, 3.6)	76.7, CH	5.47, dd (8.7, 5.4)	30.5, CH_2_	2.46–2.38, overlapped
5′	40.3, CH_2_	2.59–2.46, m	39.2, CH_2_	2.72–2.59, m	33.0, CH_2_	2.46–2.38, overlapped
6′	172.8, C		172.3, C		173.6, C	
7′	12.3, CH_3_	1.82, s	12.3, CH_3_	1.82, s	62.0, CH_2_	4.82, s
8′	60.9, CH_2_	4.13, qd (7.2, 1.4)	52.2, CH_3_	3.59, s	51.7, CH_3_	3.61, s
9′	14.3, CH_3_	1.24, t (7.2)	171.7, C		171.2, C	
10′			20.9, CH_3_	2.16, s	21.2, CH_3_	2.10, s

**Table 4:** IC_50_ values of the eight compounds against human tumor cell lines.

Cpd.	IC_50_ (Means, μM)								
	Hematologic Tumor Cell							Solid Tumor Cell	
	MOLM-13	MOLT-3	OCI-AML3	KARPAS-422	WSU-DL-CL2	CCRF-CEM	HL-60	HT-29	BXPC-3
**5**	0.15 ± 0.02	2.43 ± 0.19	2.11 ± 0.52	10.87 ± 0.65	18.74 ± 3.79	14.11 ± 1.33	21.90 ± 2.41	22.87 ± 0.25	>40
**7**	0.13 ± 0.01	2.95 ± 0.12	1.31 ± 0.59	14.13 ± 0.84	17.48 ± 4.29	18.1 ± 2.65	22.43 ± 4.22	21.98 ± 6.46	>40
**8**	0.14 ± 0.01	1.35 ± 0.30	1.73 ± 0.45	7.04 ± 0.76	8.53 ± 0.59	10.44 ± 0.95	12.76 ± 1.39	15.58 ± 2.40	32.57 ± 9.15
**9**	1.13 ± 0.03	13.22 ± 0.69	13.25 ± 2.83	33.18 ± 7.35	>40	>40	>40	>40	>40
**13**	0.48 ± 0.04	0.48 ± 0.20	3.17 ± 0.24	2.68 ± 0.22	1.07 ± 0.26	1.17 ± 0.12	2.97 ± 0.89	3.01 ± 0.49	13.98 ± 4.55
**14**	0.24 ± 0.04	0.43 ± 0.03	3.3 ± 0.80	3.67 ± 0.94	≤0.57	2.19 ± 1.12	2.46 ± 1.93	9.01 ± 2.51	>40
**15**	2.89 ± 0.19	0.84 ± 0.16	17.93 ± 1.16	8.11 ± 0.39	6.42 ± 1.96	5.98 ± 0.15	31.36 ± 8.16	28.84 ± 7.93	>40
**16**	0.06 ± 0.02	1.39 ± 0.04	1.22 ± 0.39	6.20 ± 0.74	8.12 ± 1.63	11.14 ± 1.45	12.28 ± 1.92	15.50 ± 2.96	>40
DOX	0.01 ± 0.003	ND	0.05 ± 0.01	0.12 ± 0.09	0.05 ± 0.03	0.03 ± 0.004	0.04 ± 0.003	0.17 ± 0.05	0.27 ± 0.02

Cpd., compound; DOX: doxorubicin; ND: not determined.

## Data Availability

The data presented in this study are available upon request from the corresponding author.

## Supplementary Materials

The following supporting information can be downloaded at https://www.mdpi.com/article/10.3390/md24030108/s1, 16S rRNA/ITS sequences of strains; Table S1: ^1^H NMR and ^13^C NMR data for compounds 10 and 11; Table S2: IC50 values of the compounds 1–4, 6, and 10–12 against OCI-AML3 tumor cells; Tables S3 and S4: X-ray crystallographic data for **1** and **12;** Figure S1: Key 1H-1H COSY, HMBC and NOESY correlations of compounds **10** and **11**; Figure S2–S105: UV, NMR and HRESIMS spectra of **1**–**11**.
