# Supplementary material for: Novel Marine Fungus-Derived Mycophenolic Acids That Inhibit Acute Myeloid Leukemia Cell Proliferation
Source: Mar Drugs. 2026 Mar 13;24(3):108. doi: 10.3390/md24030108 (PMC13028257; doi:10.3390/md24030108)
Supplement: Supplementary file 1 [file marinedrugs-24-00108-s001.zip › marinedrugs-4191228-supplementary.pdf]

Supporting information

Novel marine fungus derived mycophenolic acids that inhibit acute myeloid leukemia cell proliferation

Guangli Deng[1,¶], Wu Ruan[1,¶], Qun Li[1], Qingyun Peng[1], Yunan Liu[1], Lingbin Lin[1], Yuan Li[1], Qianqian Shen[2], Yangrong Zhao[2], Junfeng Wang[3], Yi Chen[2,*], Ming-Wei Wang[1,*]

1 Research Center for Deepsea Bioresources, Sanya, Hainan 572025, China
2 State Key Laboratory of Chemical Biology, Shanghai Institute of Materia Medica, Chinese Academy of Sciences, Shanghai 201203, China
3 South China Sea Institute of Oceanology, Chinese Academy of Sciences, Guangzhou 510301, China
¶ These two authors contributed equally to this work
* Correspondences to: Yi Chen (ychen@simm.ac.cn) or Ming-Wei Wang (mwwang@simm.ac.cn)

Contents

The 16S rRNA/ITs sequences of the strains *Penicillium senticosum* S7

Table S1. ^{1}H NMR and ^{13}C NMR data for compounds **10** and **11**. S8

Table S2. IC_{50} values of the compounds **1-4**, **6**, and **10-12** against OCI-AML3 tumor cells. S8

Table S3. X-ray crystallographic data for **1**. S9

Table S4. X-ray crystallographic data for **12**. S10

Figure S1. Key ^{1}H-^{1}H COSY, HMBC and NOESY correlations of compounds **10** and **11**. S11

Figure S2. ^{1}H NMR spectrum of **1** (CD_3OD, 600 MHz). S12

Figure S3. ^{13}C NMR spectrum of **1** (CD_3OD, 151 MHz). S12

Figure S4. DEPT spectrum of **1** (CD_3OD). S13

Figure S5. COSY spectrum of **1** (CD_3OD). S13

Figure S6. HSQC spectrum of **1** (CD_3OD). S14

Figure S7. HMBC spectrum of **1** (CD_3OD). S14

Figure S8. NOESY spectrum of **1** (CD_3OD). S15

Figure S9. HRESIMS spectrum of **1**. S16

Figure S10. UV spectrum of **1**. S17

Figure S11. ^{1}H NMR spectrum of **2** (CD_3OD, 600 MHz). S18

Figure S12. ^{13}C NMR spectrum of **2** (CD_3OD, 151 MHz). S18

Figure S13. DEPT spectrum of **2** (CD_3OD). S19

Figure S14. COSY spectrum of **2** (CD_3OD). S19

Figure S15. HSQC spectrum of **2** (CD_3OD). S20

Figure S16. HMBC spectrum of **2** (CD_3OD). S20

Figure S17. NOESY spectrum of **2** (CD_3OD). S21

Figure S18. HRESIMS spectrum of **2**. S22

Figure S19. UV spectrum of **2**. S23

Figure S20. ^{1}H NMR spectrum of **3** (CD_3OD, 600 MHz). S24

Figure S21. ^{13}C NMR spectrum of **3** (CD_3OD, 151 MHz). S24

Figure S22. DEPT spectrum of **3** (CD_3OD). S25

Figure S23. COSY spectrum of **3** (CD_3OD). S25

Figure S24. HSQC spectrum of **3** (CD_3OD). S26

Figure S25. HMBC spectrum of **3** (CD_3OD). S26

Figure S26. NOESY spectrum of **3** (CD_3OD). S27

Figure S27. HRESIMS spectrum of **3**. S28

Figure S28. UV spectrum of **3**. S29

Figure S29. ^{1}H NMR spectrum of **4** (CD_3OD, 600 MHz). S30

Figure S30. ^{13}C NMR spectrum of **4** (CD_3OD, 151 MHz). S30

Figure S31. DEPT spectrum of **4** (CD_3OD). S31

Figure S32. COSY spectrum of **4** (CD_3OD). S31

Figure S33. HSQC spectrum of **4** (CD_3OD). S32

Figure S34. HMBC spectrum of **4** (CD_3OD). S32

Figure S35. NOESY spectrum of **4** (CD_3OD). S33

Figure S36. HRESIMS spectrum of **4**. S34

Figure S37. UV spectrum of **4**. S35

Figure S38. ^{1}H NMR spectrum of **5** ($CDCl_3$, 600 MHz). S36

Figure S39. ^{13}C NMR spectrum of **5** ($CDCl_3$, 151 MHz). S36

Figure S40. DEPT spectrum of **5** ($CDCl_3$). S37

Figure S41. COSY spectrum of **5** ($CDCl_3$). S37

Figure S42. HSQC spectrum of **5** ($CDCl_3$). S38

Figure S43. HMBC spectrum of **5** ($CDCl_3$). S38

Figure S44. NOESY spectrum of **5** ($CDCl_3$). S39

Figure S45. HRESIMS spectrum of **5**. S40

Figure S46. UV spectrum of **5**. S41

Figure S47. ^{1}H NMR spectrum of **6** ($CDCl_3$, 600 MHz). S42

Figure S48. ^{13}C NMR spectrum of **6** ($CDCl_3$, 151 MHz). S42

Figure S49. DEPT spectrum of **6** ($CDCl_3$). S43

Figure S50. COSY spectrum of **6** ($CDCl_3$). S43

Figure S51. HSQC spectrum of **6** ($CDCl_3$). S44

Figure S52. HMBC spectrum of **6** ($CDCl_3$). S44

Figure S53. NOESY spectrum of **6** ($CDCl_3$). S45

Figure S54. HRESIMS spectrum of **6**. S46

Figure S55. UV spectrum of **6**. S47

Figure S56. ^{1}H NMR spectrum of **7** ($CDCl_3$, 600 MHz). S48

Figure S57. ^{13}C NMR spectrum of **7** ($CDCl_3$, 151 MHz). S48

Figure S58. DEPT spectrum of **7** ($CDCl_3$). S49

Figure S59. COSY spectrum of **7** ($CDCl_3$). S49

Figure S60. HSQC spectrum of **7** ($CDCl_3$). S50

Figure S61. HMBC spectrum of **7** ($CDCl_3$). S50

Figure S62. NOESY spectrum of **7** ($CDCl_3$). S51

Figure S63. HRESIMS spectrum of **7**. S52

Figure S64. UV spectrum of **7**. S53

Figure S65. HRESIMS spectrum of **7R**. S54

Figure S66. HRESIMS spectrum of **7Ra**. S55

Figure S67. HRESIMS spectrum of **7Rb**. S56

Figure S68. ^{1}H NMR spectrum of **7Ra** ($CDCl_3$, 600 MHz). S57

Figure S69. ^{1}H NMR spectrum of **7Rb** ($CDCl_3$, 600 MHz). S57

Figure S70. ^{1}H NMR spectrum of **8** (CD_3OD, 600 MHz). S58

Figure S71. ^{13}C NMR spectrum of **8** (CD_3OD, 151 MHz). S58

Figure S72. DEPT spectrum of **8** (CD_3OD). S59

Figure S73. COSY spectrum of **8** (CD_3OD). S59

Figure S74. HSQC spectrum of **8** (CD_3OD). S60

Figure S75. HMBC spectrum of **8** (CD_3OD). S60

Figure S76. NOESY spectrum of **8** (CD_3OD). S61

Figure S77. HRESIMS spectrum of **8**. S62

Figure S78. UV spectrum of **8**. S63

Figure S79. ^1H NMR spectrum of **9** ($CDCl_3$, 600 MHz). S64

Figure S80. ^{13}C NMR spectrum of **9** ($CDCl_3$, 151 MHz). S64

Figure S81. DEPT spectrum of **9** ($CDCl_3$). S65

Figure S82. COSY spectrum of **9** ($CDCl_3$). S65

Figure S83. HSQC spectrum of **9** ($CDCl_3$). S66

Figure S84. HMBC spectrum of **9** ($CDCl_3$). S66

Figure S85. NOESY spectrum of **9** ($CDCl_3$). S67

Figure S86. HRESIMS spectrum of **9**. S68

Figure S87. UV spectrum of **9**. S69

Figure S88. ^1H NMR spectrum of **10** ($CDCl_3$, 600 MHz). S70

Figure S89. ^{13}C NMR spectrum of **10** ($CDCl_3$, 151 MHz). S70

Figure S90. DEPT spectrum of **10** ($CDCl_3$). S71

Figure S91. COSY spectrum of **10** ($CDCl_3$). S71

Figure S92. HSQC spectrum of **10** ($CDCl_3$). S72

Figure S93. HMBC spectrum of **10** ($CDCl_3$). S72

Figure S94. NOESY spectrum of **10** ($CDCl_3$). S73

Figure S95. HRESIMS spectrum of **10**. S74

Figure S96. UV spectrum of **10**. S75

Figure S97. ^1H NMR spectrum of **11** ($CDCl_3$, 600 MHz). S76

Figure S98. ^{13}C NMR spectrum of **11** ($CDCl_3$, 151 MHz). S76

Figure S99. DEPT spectrum of **11** ($CDCl_3$). S77

Figure S100. COSY spectrum of **11** ($CDCl_3$). S77

Figure S101. HSQC spectrum of **11** ($CDCl_3$). S78

Figure S102. HMBC spectrum of **11** ($CDCl_3$).. S78

Figure S103. NOESY spectrum of **11** ($CDCl_3$)... S79

Figure S104. HRESIMS spectrum of **11**. S80

Figure S105. UV spectrum of **11**. S81

The physiochemical data of compounds 10 and **11**. S82

The 16S rRNA/ITs sequences of the strains *Penicillium senticosum* RCDB005.

CTTCCGTAGGTGAACCTGCGGAAGGATCATTACCGAGTGAGGGCCCTCTGGGTCCAAC

CTCCCACCCGTGTCTATCGTACCTTGTTGCTTCGGCGGGCCCGCCGCAAGGCCGCCGG

GGGGCTTCCGTCCCCGGGCCCGCGCCCGCCGAAGACACCTGTGAACGCTGTATGAAG

ATTGCAGTCTGAGCGAAAAGCTAAATTTGTTAAAACTTTCAACAACGGATCTCTTGGT

TCCGGCATCGATGAAGAACGCAGCGAAATGCGATAAGTAATGTGAATTGCAGAATTC

AGTGAATCATCGAGTCTTTGAACGCACATTGCGCCCCCTGGTATTCCGGGGGGCATGC

CTGTCCGAGCGTCATTGCTGCCCTCAAGCACGGCTTGTGTGTTGGGCCCTCGTCCCCC

GGGACGGGCCCGAAAGGCAGCGGCGGCACCGCGTCCGGTCCTCGAGCGTATGGGGC

TTCGTCACCCGCTCTGTAGGCCTGGCCGGCGCCTGCCGACACCATCAATCTTTTTTCCA

GGTTGACCTCGGATCAGGTAGGGATACCCGCTGAACTTAAGCATATCAATAAGCGGAG

GAA

Table S1. ^{1}H NMR and ^{13}C NMR data for compounds **10** and **11**.

	**10**		**11**	
**Position**	**δ_C, type**	**δ_H (J in Hz)**	**δ_C, type**	**δ_H (J in Hz)**
1	173.6, C		172.9, C	
3	70.0, CH_2	5.19, s	70.5, CH_2	5.23, s
3a	144.4, C		146.2, C	
4	113.0, C		104.4, CH	6.48, s
5	160.4, C		164.9, C	
5-OMe			56.3, CH_3	3.89, s
6	110.5, C		116.7, C	
7	153.2, C		154.6, C	
7a	103.2, C		96.2, C	
8	11.0 CH_3	2.07, s		
1′	21.9, CH_2	3.45, d (7.1)	21.7, CH_2	3.35, d (7.2)
2′	121.5, CH	5.24, t (9.5)	122.4, CH	5.21, t (3.9)
3′	138.6, C		134.2, C	
4′,	32.6, CH_2	2.45, dd (9.7, 7.8)	33.1, CH_2	2.39, dd (8.9, 6.4)
5′	34.7, CH_2	2.38, dd (14.8, 7.5)	34.9, CH_2	2.28, dd (9.2, 6.5)
6′	173.4, C		174.1, C	
7′	16.4, CH_3	1.85, s	16.1, CH_3	1.78, s
8′	51.8, CH_3	3.65, s	51.6, CH_3	3.61, s

Recorded in $CDCl_3$.

Table S2. IC_{50} values of the compounds **1-4**, **6**, and **10-12** against OCI-AML3 tumor cells.

**Cpd.**	**IC_{50} (mean±SD, μM)**
	**OCI-AML3**
**1**	134.54±7.77
**2**	≥132.58±25.83
**3**	98.61±28.23
**4**	122.37±10.27
**6**	73.43±10.52
**10**	40.85±5.40

**11**	38.42±5.34
**12**	106.52±4.36
DOX	0.05±0.01

DOX: doxorubicin

Table S3. X-ray crystallographic data for **1**.

Empirical formula	$C_{16}H_{18}O_5$
Formula weight	290.30
Temperature [K]	302.00
Crystal system	monoclinic
Space group (number)	$P2_1$ (4)
a [Å]	5.02400(10)
b [Å]	13.6427(4)
c [Å]	20.9641(6)
α [°]	90
β [°]	96.049(2)
γ [°]	90
Volume [Å^3]	1428.90(7)
Z	4
ρ_{calc} [gcm^{-3}]	1.349
μ [mm^{-1}]	0.831
F(000)	616
Crystal size [mm^3]	0.228×0.142×0.015
Crystal colour	metallic colourless
Crystal shape	plate
Radiation	CuK_α (λ=1.54178 Å)
2θ range [°]	7.74 to 136.90 (0.83 Å)
Index ranges	−5 ≤ h ≤ 6
	−16 ≤ k ≤ 15
	−23 ≤ l ≤ 25
Reflections collected	27611

Independent reflections	4888<br>R_{int} = 0.0635<br>R_{sigma} = 0.0494
Completeness to θ = 67.679°	98.3 %
Data / Restraints / Parameters	4888/1/388
Goodness-of-fit on F^2	1.005
Final *R* indexes [I≥2σ(I)]	R_1 = 0.0466<br>wR_2 = 0.1175
Final *R* indexes [all data]	R_1 = 0.0798<br>wR_2 = 0.1347
Largest peak/hole [eÅ^{-3}]	0.19/-0.15
Flack X parameter	-0.1(2)
Extinction coefficient	0.0061(9)

Table S4. X-ray crystallographic data for **12**.

Empirical formula	$C_{16}H_{18}O_5$
Formula weight	290.30
Temperature [K]	302.00
Crystal system	triclinic
Space group (number)	$P\bar{1}$ (2)
a [Å]	4.5267(3)
b [Å]	11.7847(7)
c [Å]	14.4718(9)
α [°]	71.277(4)
β [°]	86.160(4)
γ [°]	85.886(4)
Volume [Å^3]	728.48(8)
Z	2
ρ_{calc} [gcm^{-3}]	1.323
μ [mm^{-1}]	0.815

$F(000)$	308
Crystal size [mm^3]	0.306×0.02×0.004
Crystal color	colorless
Crystal shape	plate
Radiation	CuK_{α} (λ=1.54178 Å)
2θ range [°]	6.46 to 133.13 (0.84 Å)
Index ranges	$-5 \leq h \leq 5$<br>$-13 \leq k \leq 13$<br>$-17 \leq l \leq 17$
Reflections collected	31113
Independent reflections	2584<br>R_{int} = 0.0517<br>R_{sigma} = 0.0229
Completeness to θ = 66.567°	99.9 %
Data / Restraints / Parameters	2584/26/215
Goodness-of-fit on F^2	1.093
Final R indexes [$I \geq 2\sigma(I)$]	R_1 = 0.0557<br>wR_2 = 0.1615
Final R indexes [all data]	R_1 = 0.0709<br>wR_2 = 0.1748
Largest peak/hole [eÅ^{-3}]	0.49/-0.30

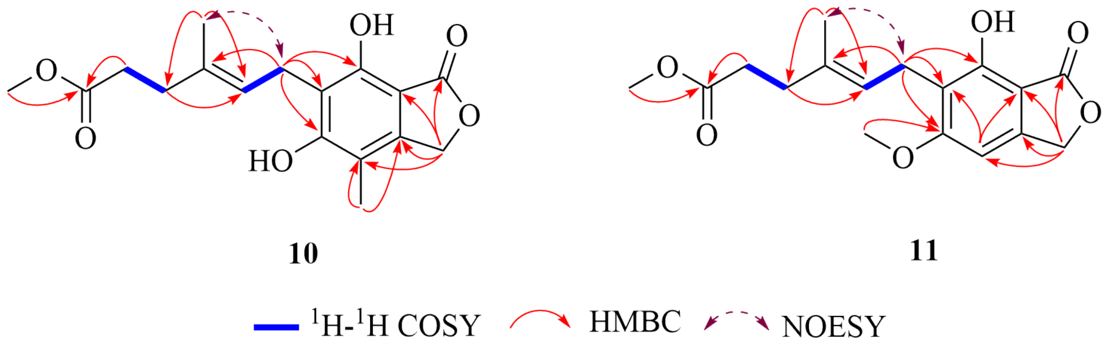

Figure S1. Key ^{1}H-^{1}H COSY, HMBC and NOESY correlations of compounds **10** and **11**.

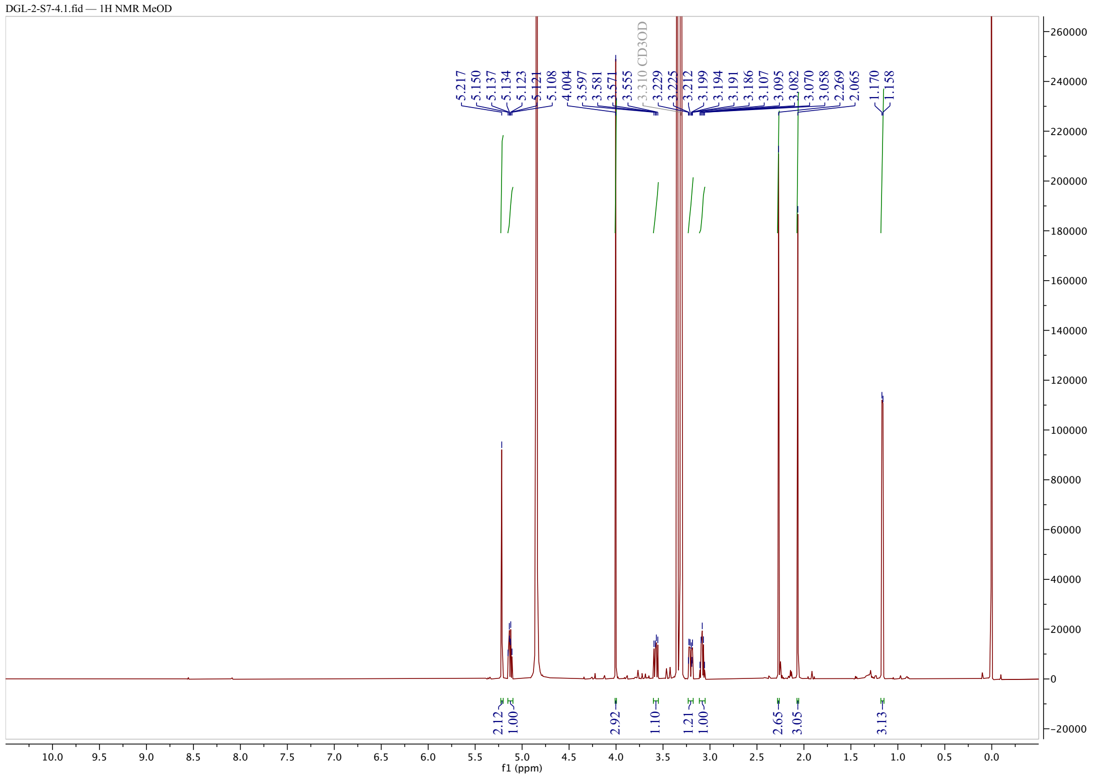

Figure S2. ^{1}H NMR spectrum of **1** (CD_3OD, 600 MHz).

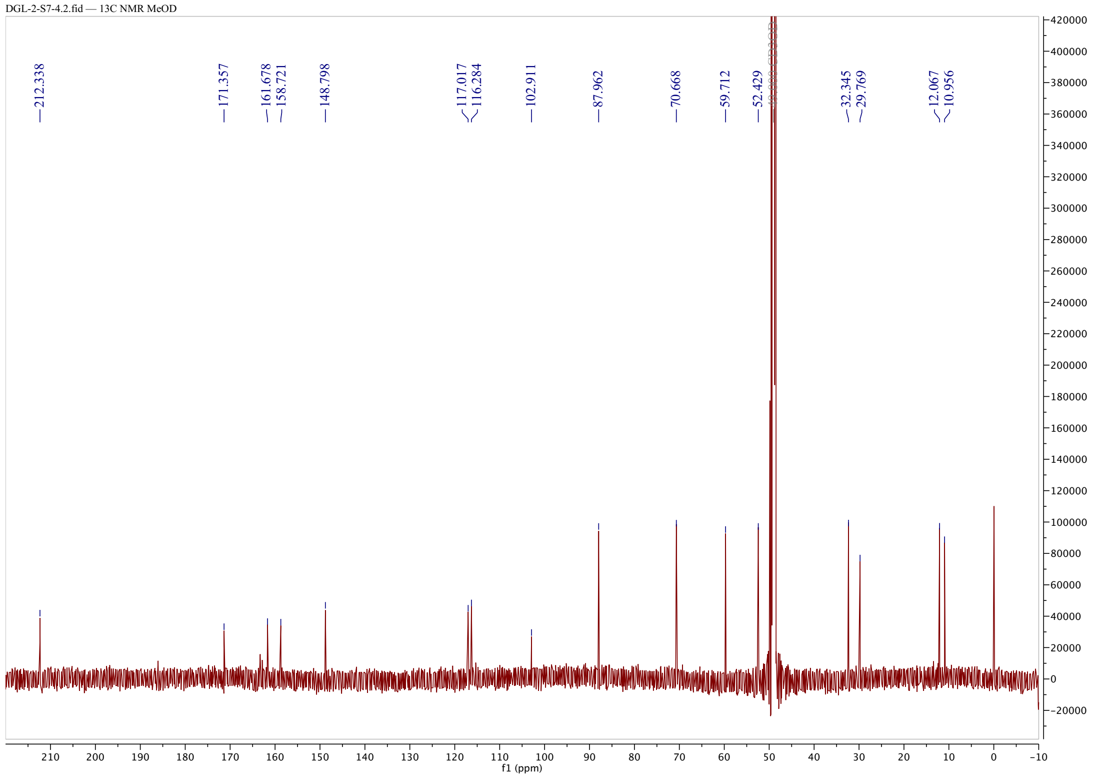

Figure S3. ^{13}C NMR spectrum of **1** (CD_3OD, 151 MHz).

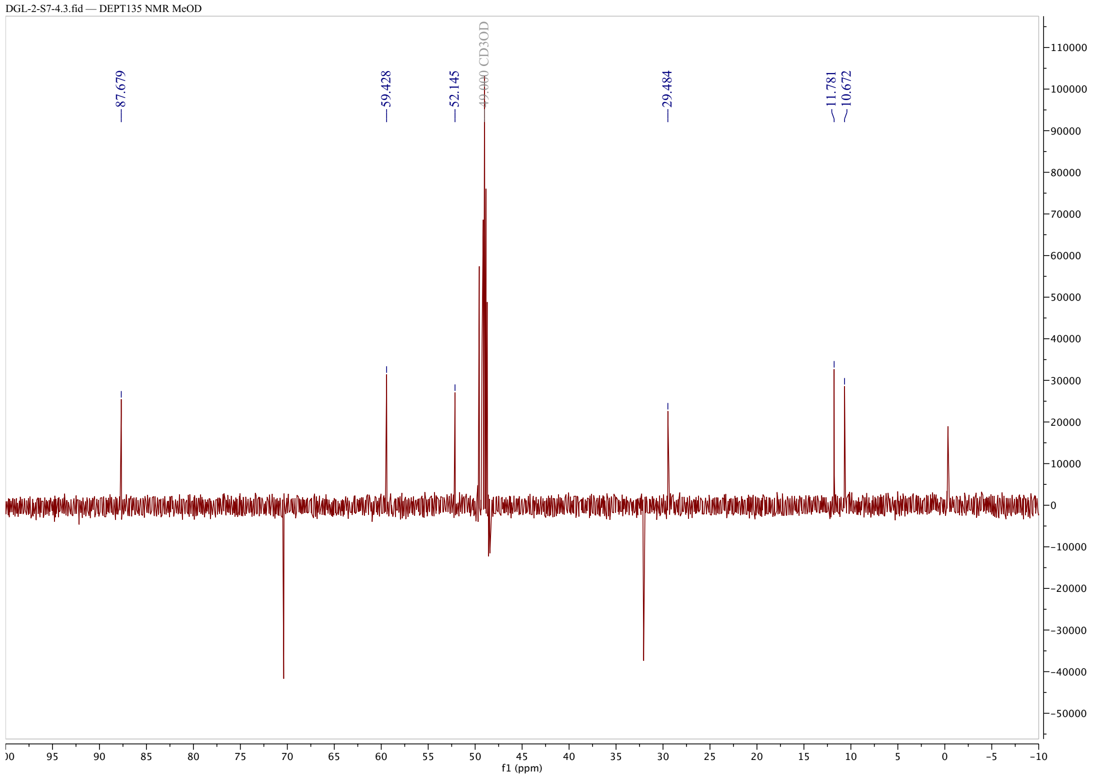

Figure S4. DEPT spectrum of **1** (CD_3OD).

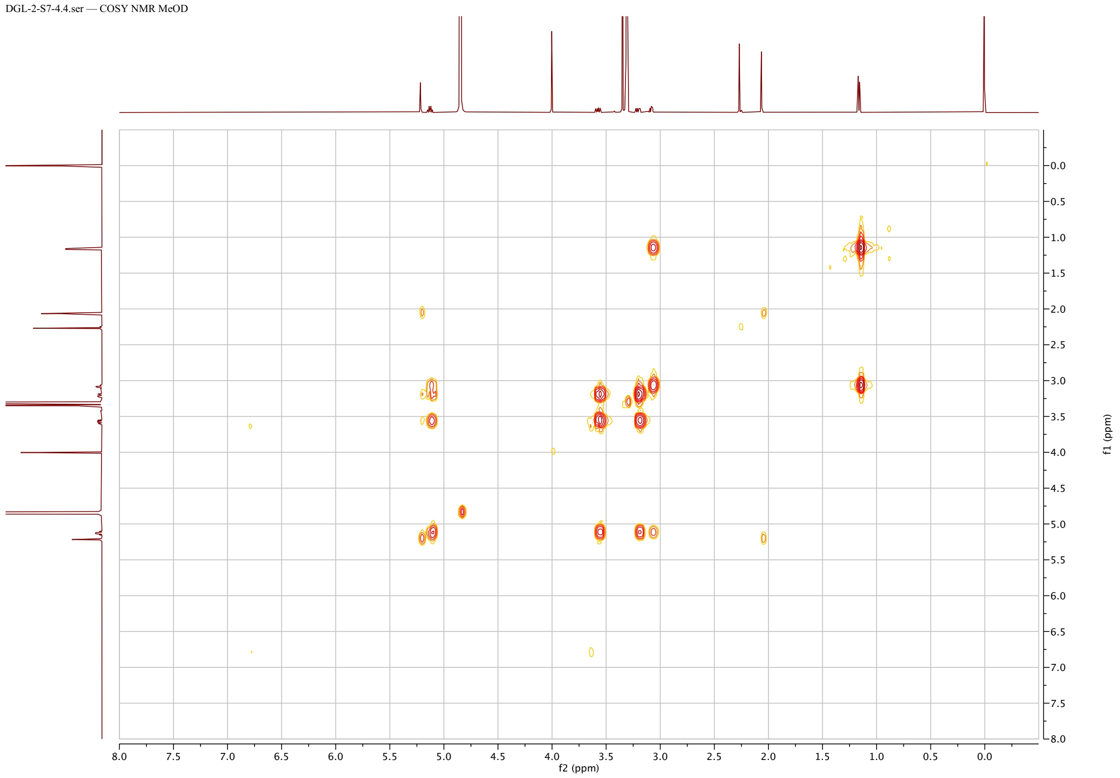

Figure S5. COSY spectrum of **1** (CD_3OD).

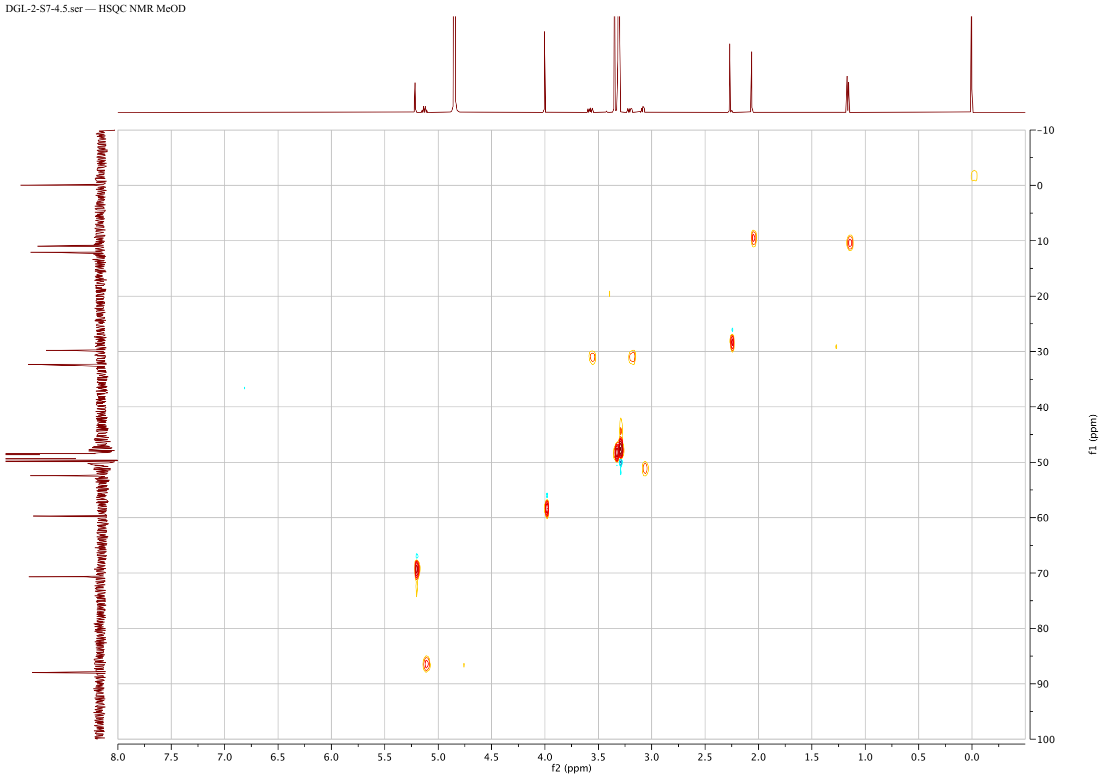

Figure S6. HSQC spectrum of **1** (CD3OD).

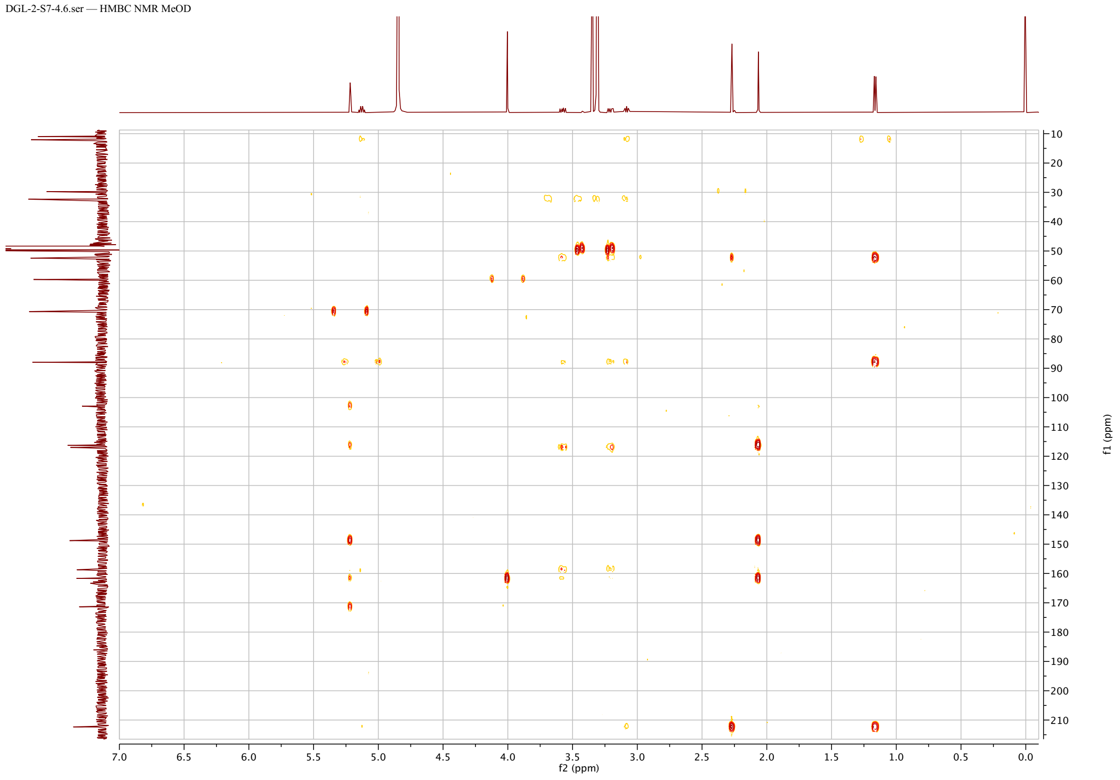

Figure S7. HMBC spectrum of **1** (CD3OD).

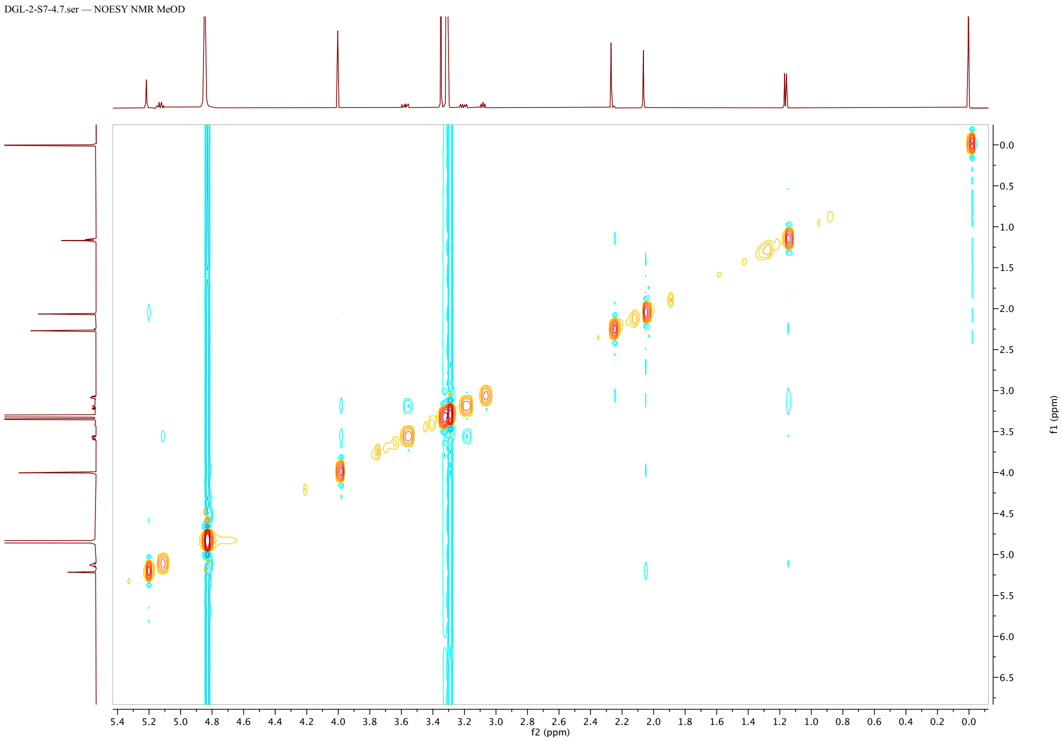

Figure S8. NOESY spectrum of **1** (CD_3OD).

Analysis Report

Sample Information

**Name**	DGL-S7-4-POS-001	**Data File Path**	D:\Projects\2023\Data\RCDB\DGL\20250910\DGL-S7-4-POS-001.d
**Inj. Vol. (ul)**	1	**Method Path (Acq)**	D:\Projects\2023\Methods\General positive organic analysis method-2 .m
**Position**	P1-a4	**Acq. Time (Local)**	9/10/2025 2:24:59 PM (UTC+08:00)
**MS Type**	QTOF	**Ion Polarity**	Positive
**Instrument**	G6545B	**Version (Acq SW)**	6200 series TOF/6500 series Q-TOF (11.0.221.1)
**Operator**	SYSTEM (SYSTEM)		

Sample Spectra

+ Scan (rt: 9.895 min)

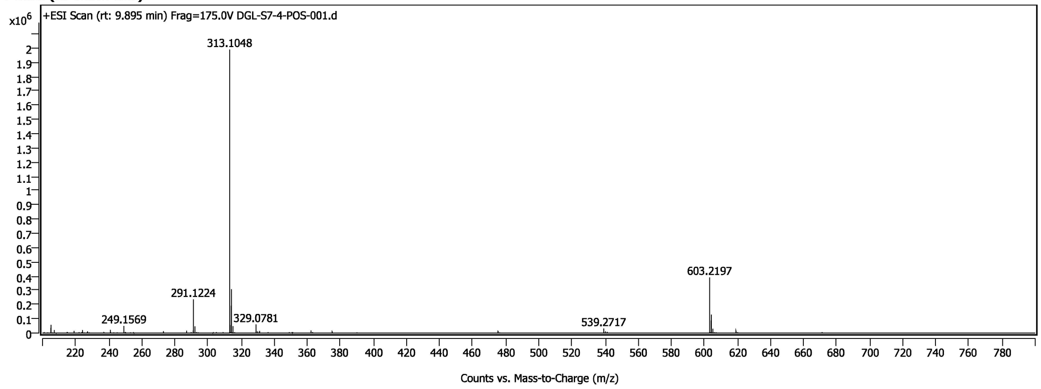

Spectrum Peaks

m/z	z	m/z (Calc)	Ion Species	Formula	Diff (ppm)	Diff (mDa)
291.1224	1	291.1227	(M+H)+	C16 H18 O5	-1.20	-0.3

Spectrum Identification Table

Formula	m/z	Mass	Species	Score	Diff (ppm)	Diff (mDa)
C16 H18 O5	291.1224	290.1151	(M+H)+	98.98	-1.25	-0.4
C15 H12 N7	291.1224	290.1152	(M+H)+	98.36	-0.70	-0.2
C14 H16 N3 O4	291.1224	290.1151	(M+H)+	94.25	3.64	1.1
C17 H14 N4 O	291.1224	290.1151	(M+H)+	90.57	-5.59	-1.6

MassHunter Qual 10.0
(End of Report)

MassHunter Qualitative Analysis   Page 1 of 1   Generated at 3:10 PM on 9/10/2025

Figure S9. HRESIMS spectrum of **1**.

RCDB

样品ID	日期和时间	积分时间 (s)	扫描速度	数据间隔	起始波长	结束波长	带宽	型号#
样品S7-4-15	2025/2/25 14:33:15	0.05	1200.00	1.00	200.00	400.00	0.5 nm	Evo350

峰：:

nm	Abs
222.238	1.812
251.948	0.622
306.668	0.339

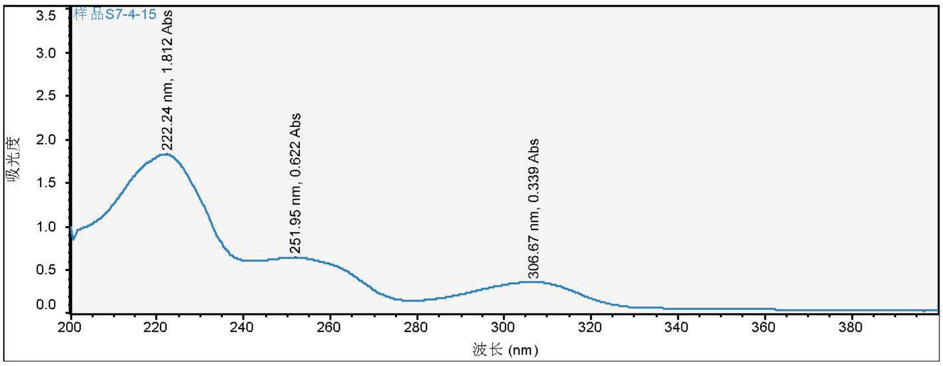

INSIGHT Software 第 1 页，共 1 页

Figure S10. UV spectrum of **1**.

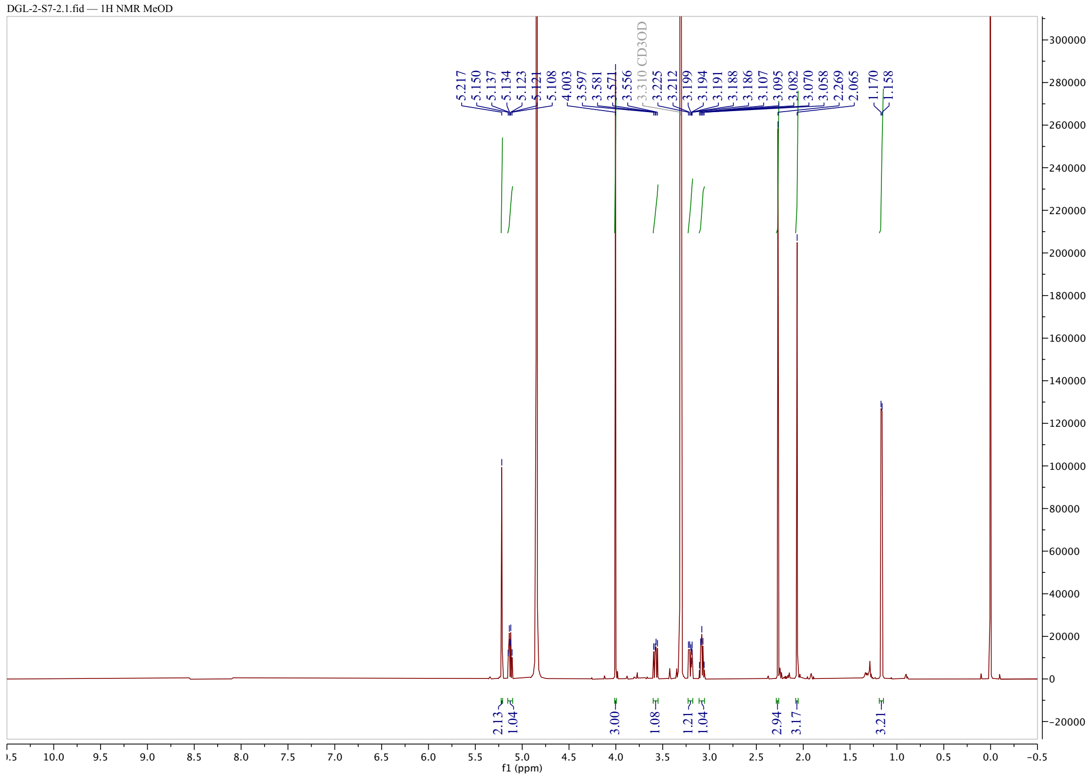

Figure S11. ^{1}H NMR spectrum of **2** (CD_3OD, 600 MHz).

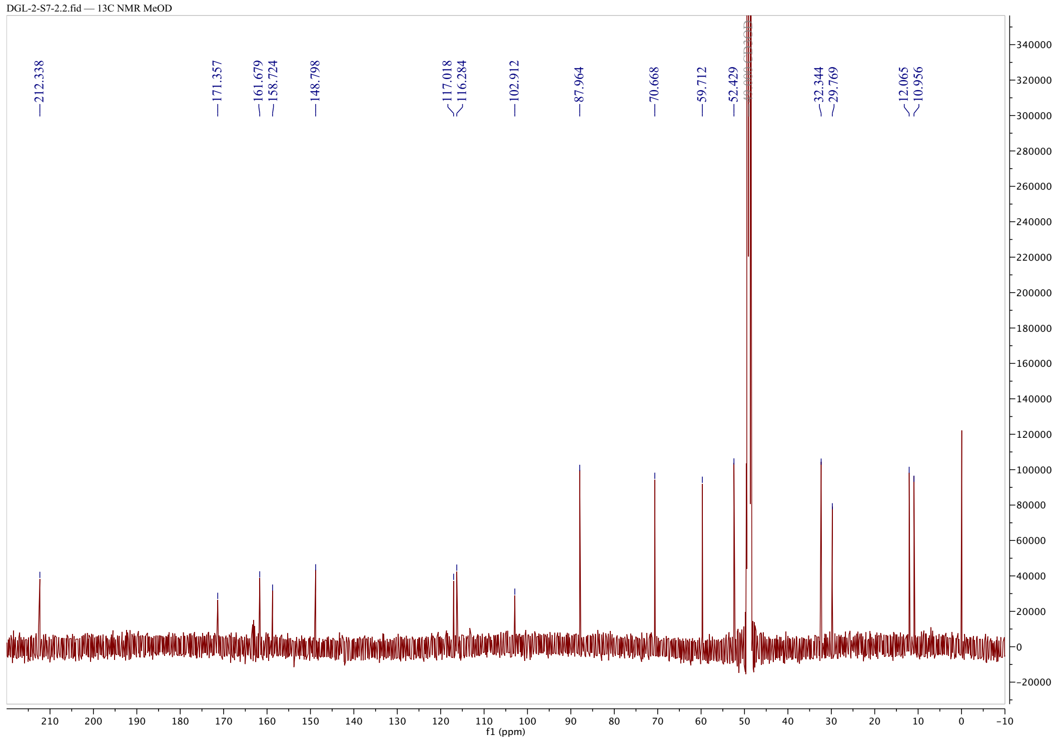

Figure S12. ^{13}C NMR spectrum of **2** (CD_3OD, 151 MHz).

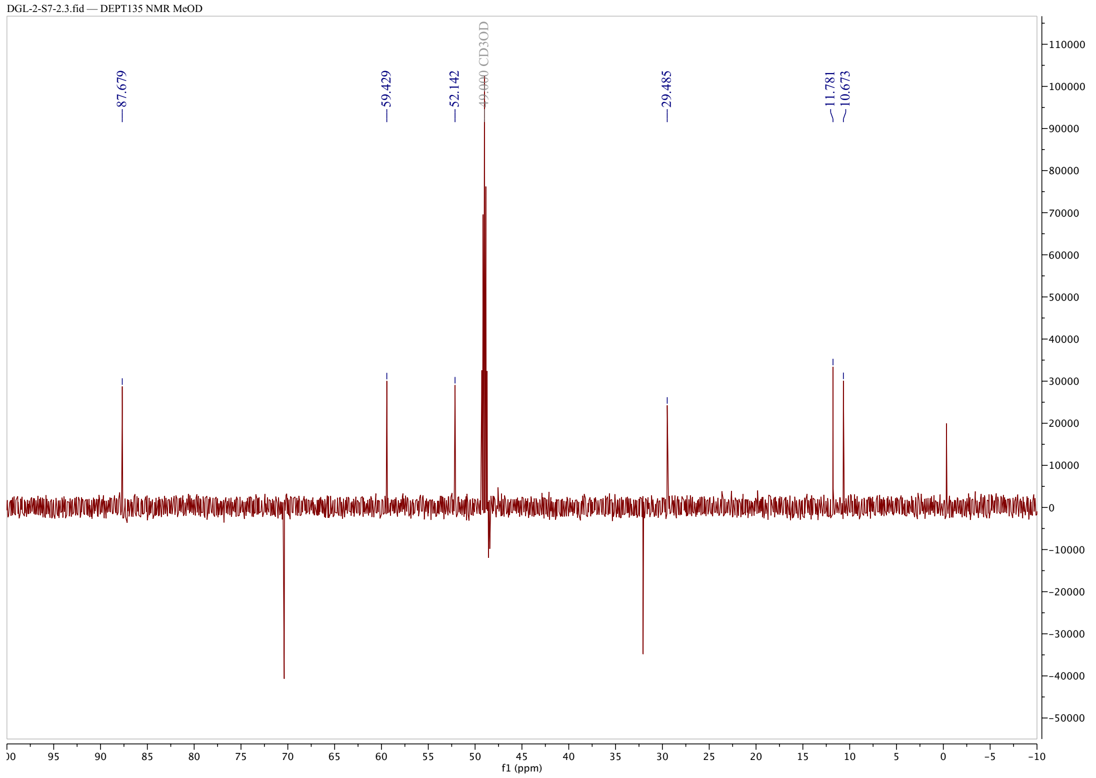

Figure S13. DEPT spectrum of **2** (CD_3OD).

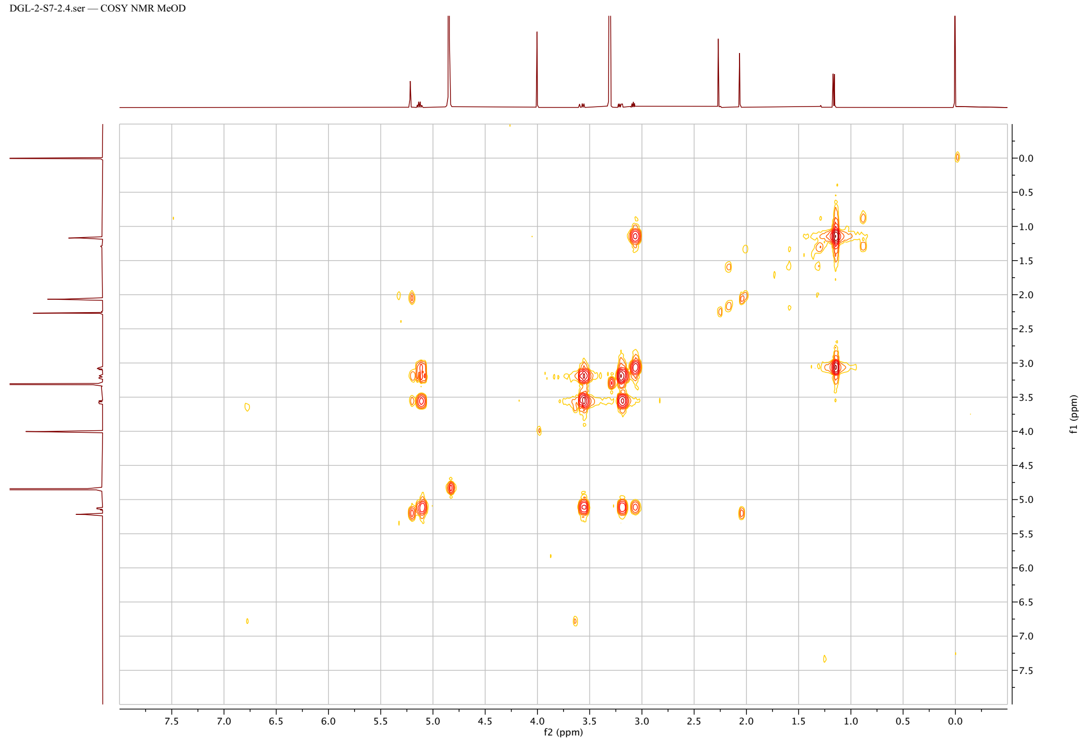

Figure S14. COSY spectrum of **2** (CD_3OD).

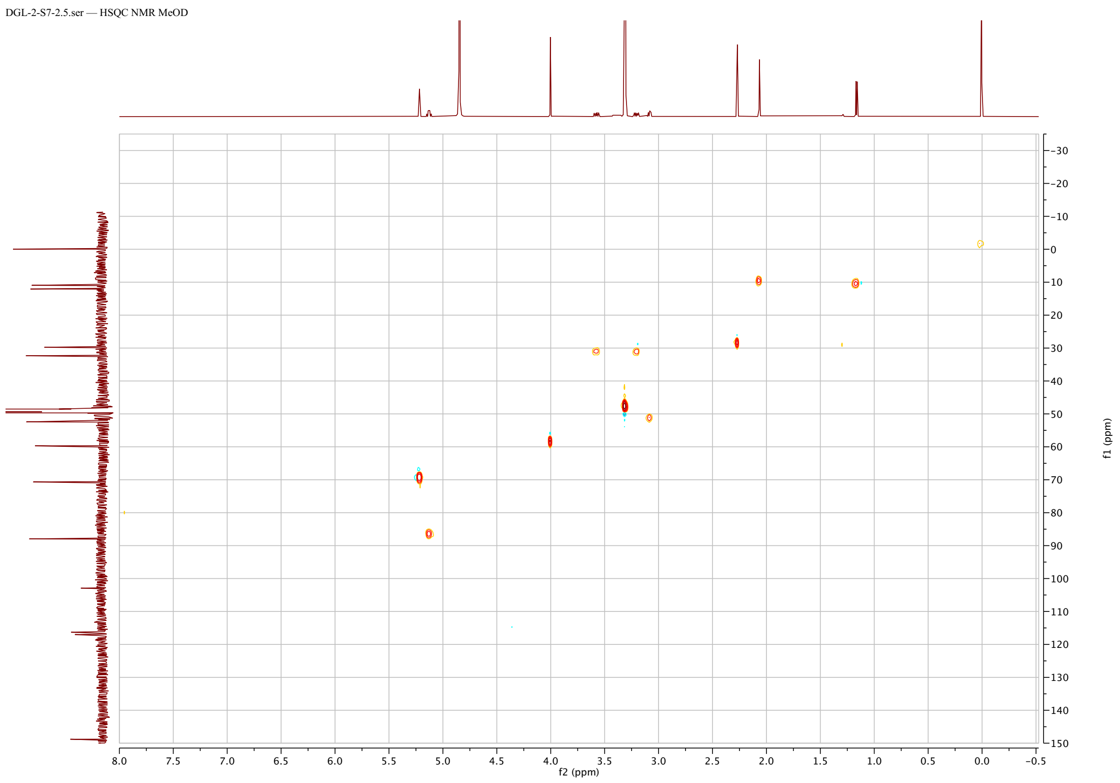

Figure S15. HSQC spectrum of **2** (CD_3OD).

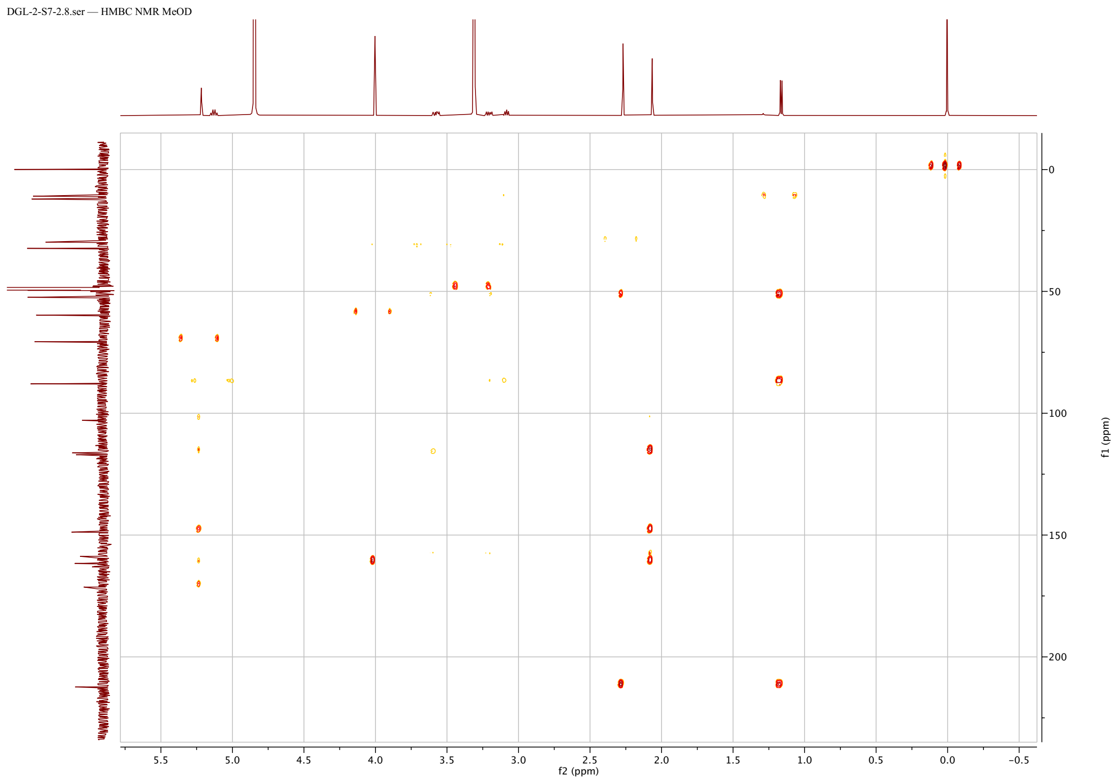

Figure S16. HMBC spectrum of **2** (CD_3OD).

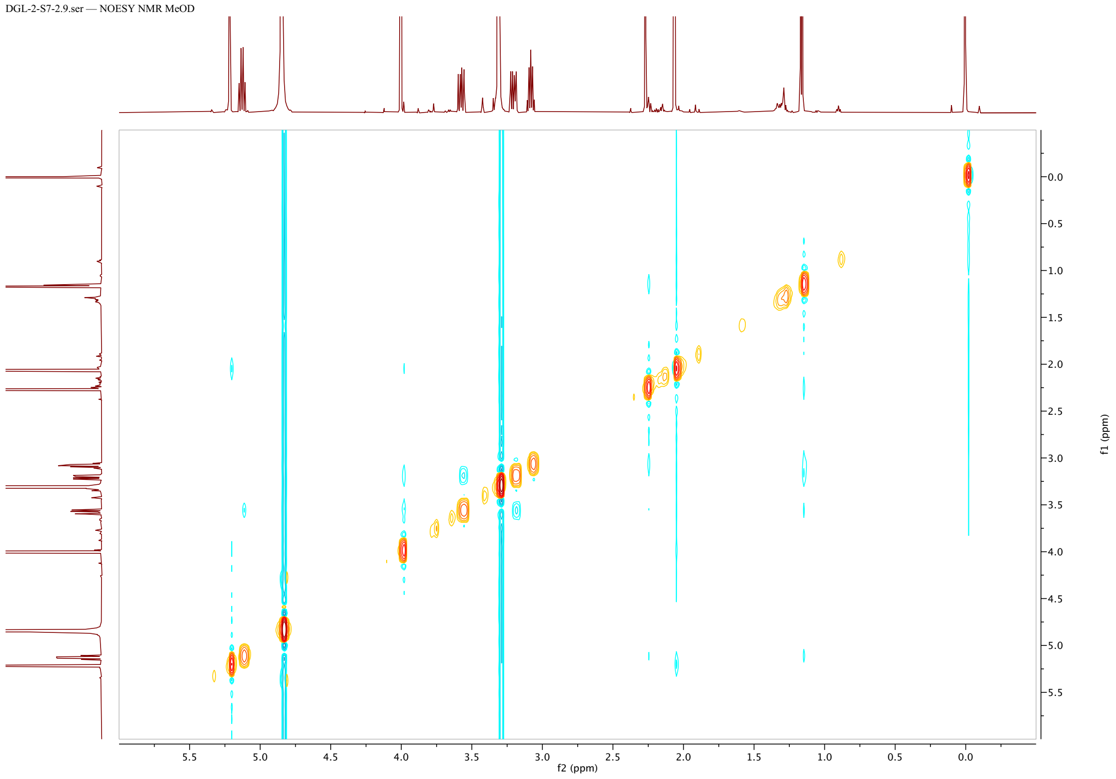

Figure S17. NOESY spectrum of **2** (CD_3OD).

Analysis Report

Sample Information

**Name**	DGL-S7-2-POS-001	**Data File Path**	D:\Projects\2023\Data\RCDB\DGL\20250910\DGL-S7-2-POS-001.d
**Inj. Vol. (ul)**	1	**Method Path (Acq)**	D:\Projects\2023\Methods\General positive organic analysis method-2 .m
**Position**	P1-a3	**Acq. Time (Local)**	9/10/2025 1:48:18 PM (UTC+08:00)
**MS Type**	QTOF	**Ion Polarity**	Positive
**Instrument**	G6545B	**Version (Acq SW)**	6200 series TOF/6500 series Q-TOF (11.0.221.1)
**Operator**	SYSTEM (SYSTEM)		

Sample Spectra

+ Scan (rt: 9.822 min)

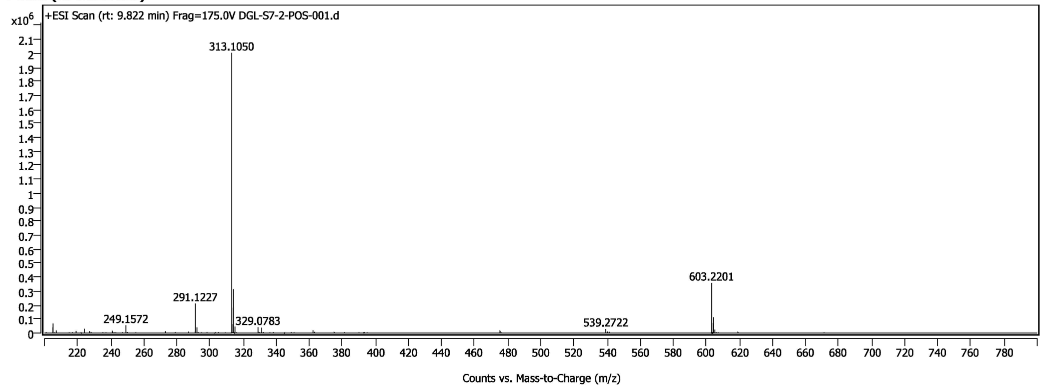

Spectrum Peaks

m/z	z	m/z (Calc)	Ion Species	Formula	Diff (ppm)	Diff (mDa)
291.1227	1	291.1227	(M+H)+	C16 H18 O5	-0.03	0.0

Spectrum Identification Table

Formula	m/z	Mass	Species	Score	Diff (ppm)	Diff (mDa)
C16 H18 O5	291.1227	290.1154	(M+H)+	99.61	0.08	0.0
C15 H12 N7	291.1227	290.1156	(M+H)+	97.75	0.63	0.2
C17 H14 N4 O	291.1227	290.1155	(M+H)+	93.42	-4.26	-1.2
C14 H16 N3 O4	291.1227	290.1155	(M+H)+	91.16	4.98	1.4

MassHunter Qual 10.0
(End of Report)

MassHunter Qualitative Analysis Page 1 of 1 Generated at 3:10 PM on 9/10/2025

Figure S18. HRESIMS spectrum of **2**.

RCDB

#	样品ID	用户名	日期和时间	型号#	起始波长	结束波长
2	S7-2 30ug/ml	DELL	2025/2/20 14:46:18	Evo350	200.00	400.00

峰：:

nm	Abs
222.233	1.392
251.677	0.474
306.670	0.266

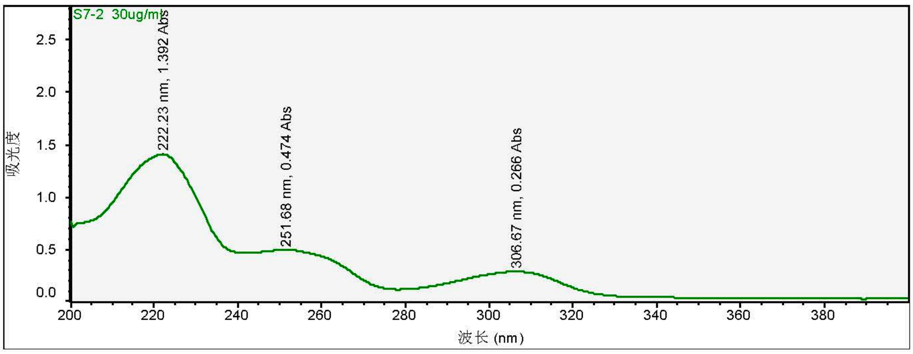

INSIGHT Software 第 1 页，共 1 页

Figure S19. UV spectrum of **2**.

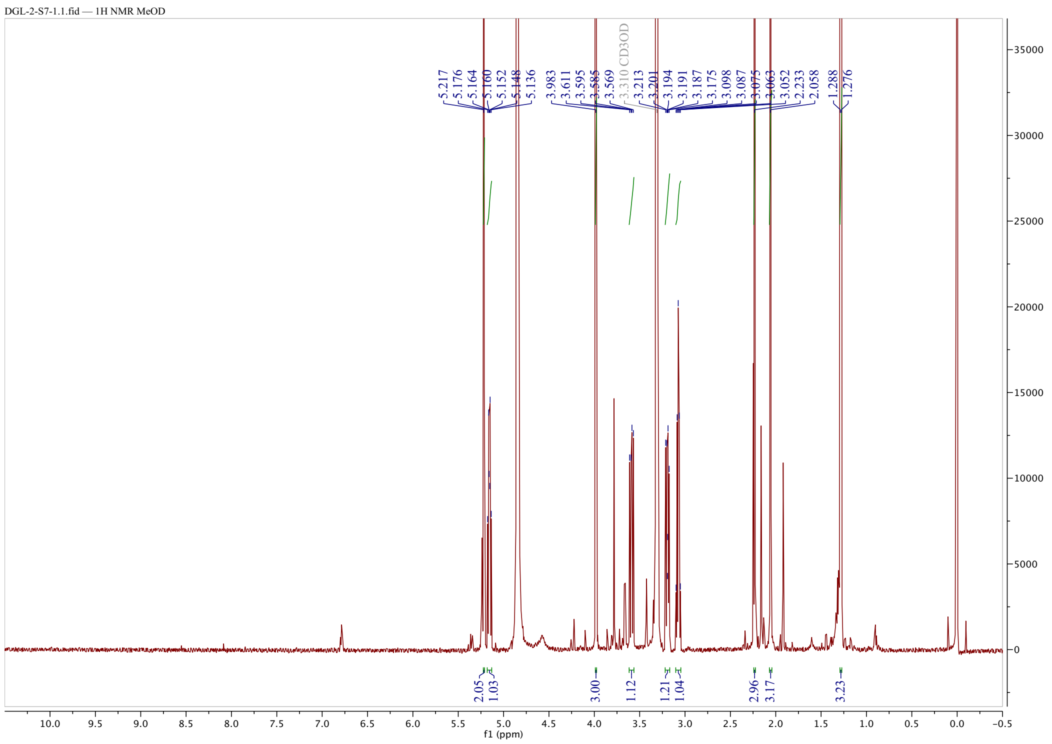

Figure S20. ^{1}H NMR spectrum of **3** (CD_3OD, 600 MHz).

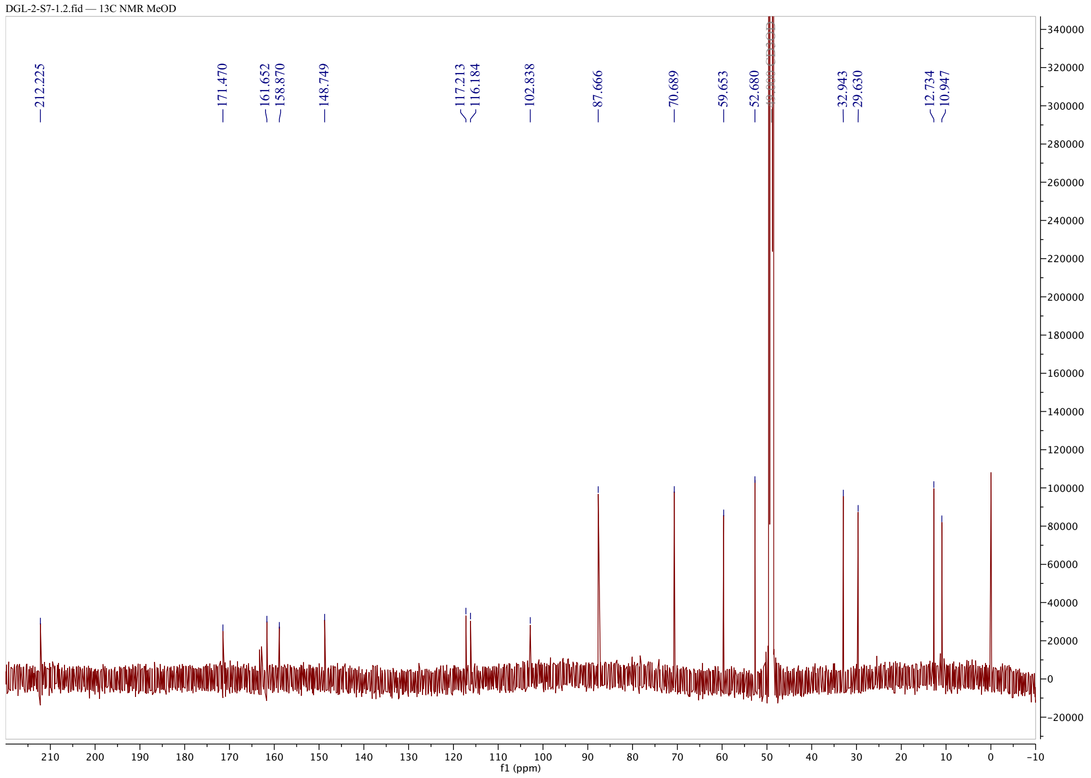

Figure S21. ^{13}C NMR spectrum of **3** (CD_3OD, 151 MHz).

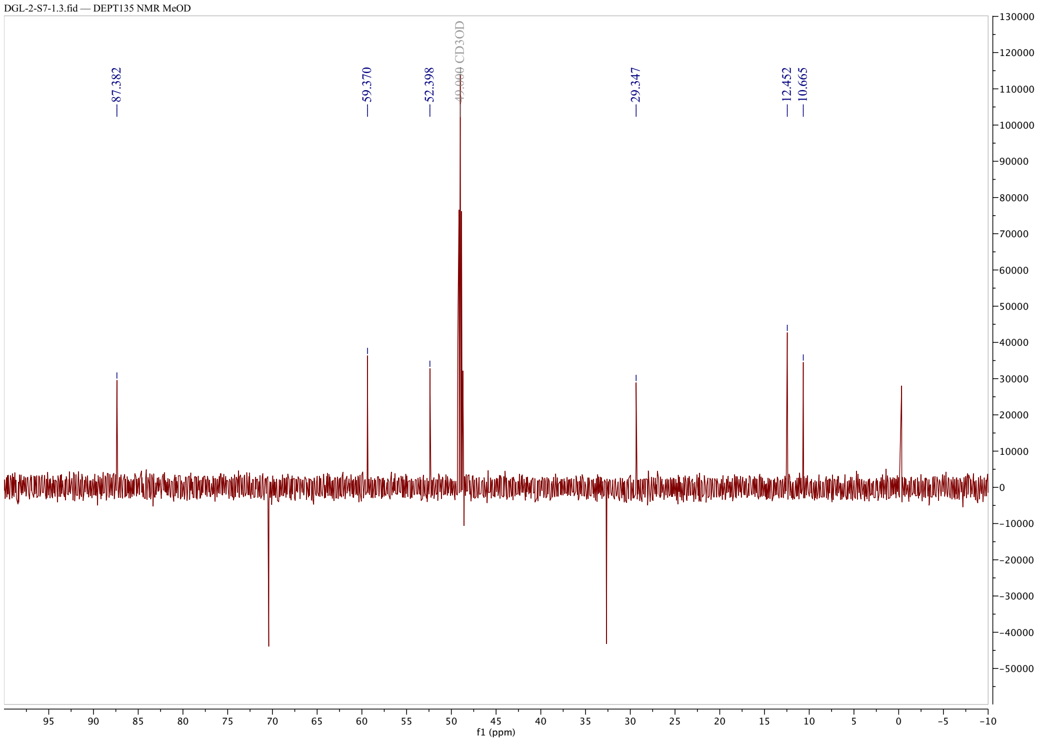

Figure S22. DEPT spectrum of **3** (CD_3OD).

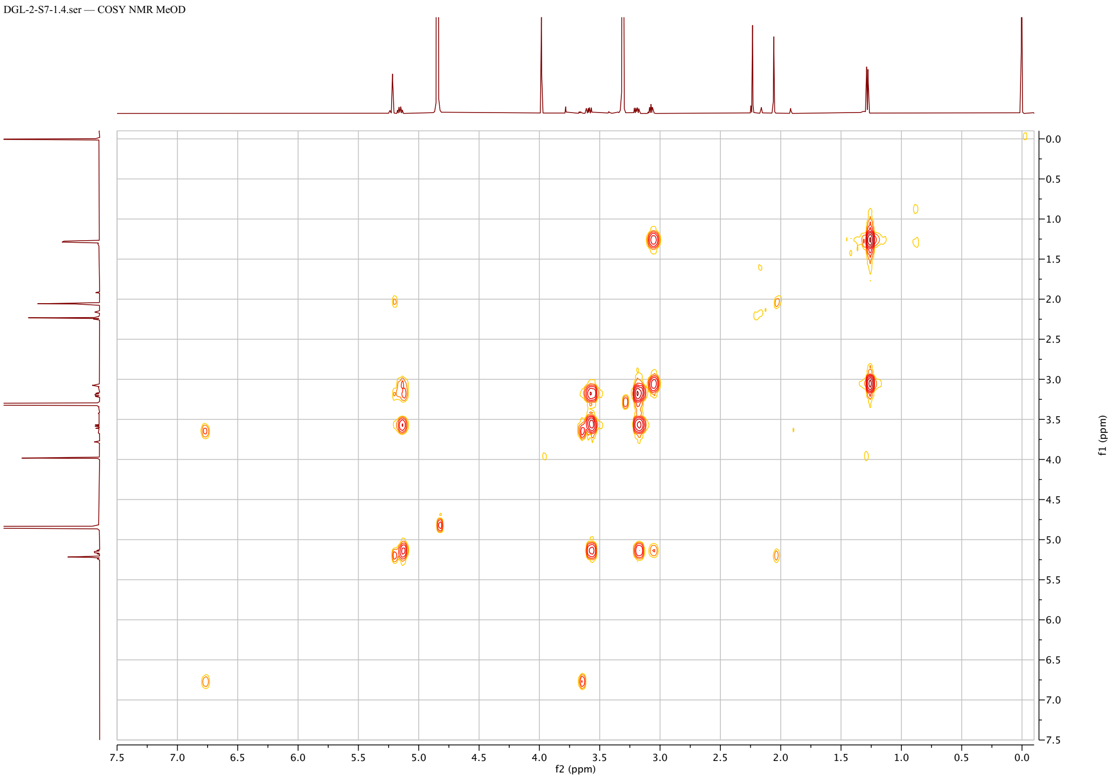

Figure S23. COSY spectrum of **3** (CD_3OD).

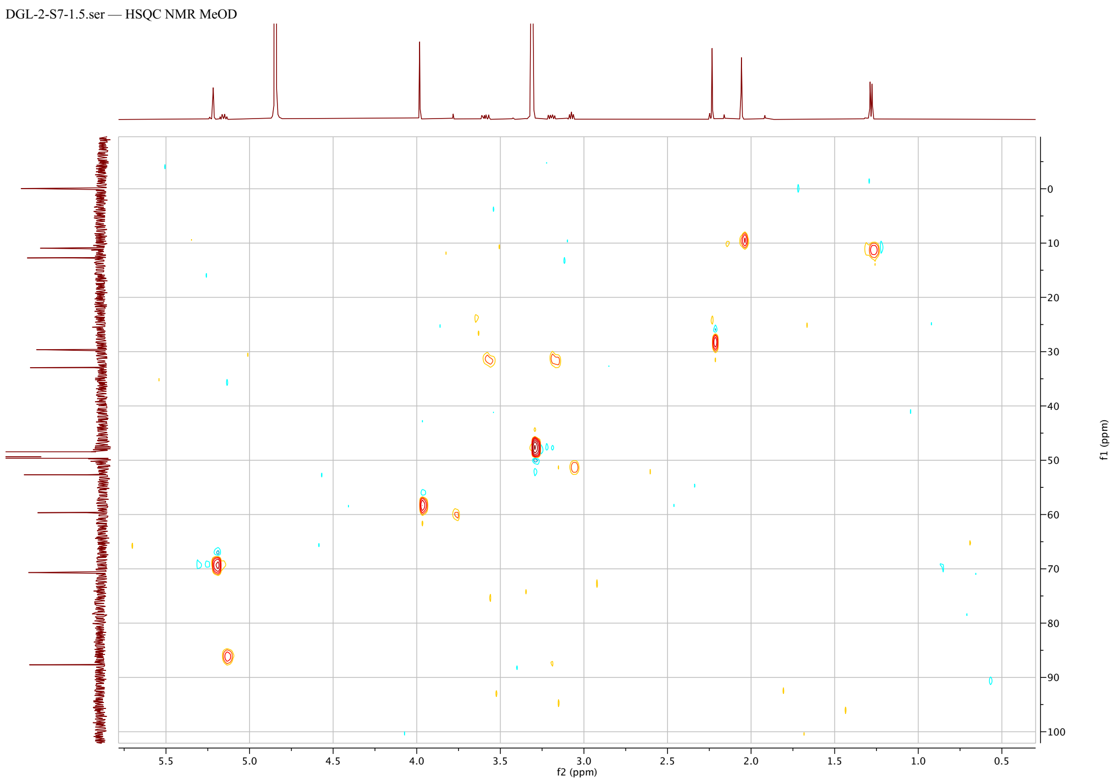

Figure S24. HSQC spectrum of **3** (CD_3OD).

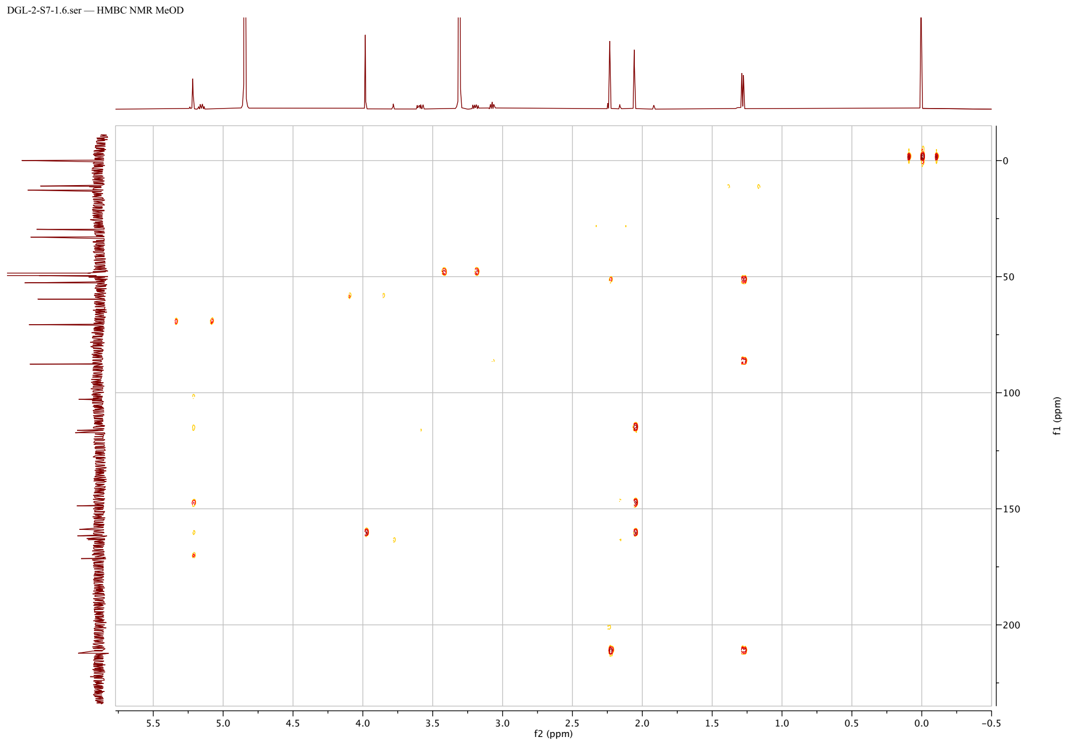

Figure S25. HMBC spectrum of **3** (CD_3OD).

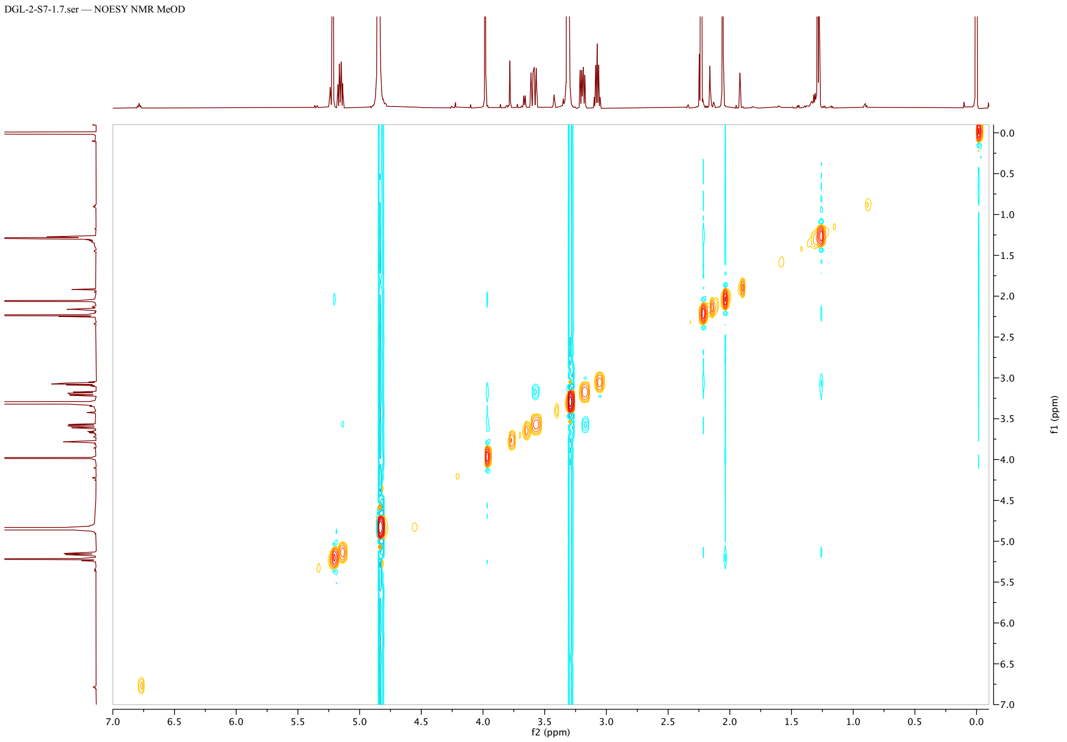

Figure S26. NOESY spectrum of **3** (CD_3OD).

Analysis Report

Agilent | Trusted Answers

Sample Information

**Name**	DGL-S7-1-POS-001	**Data File Path**	D:\Projects\2023\Data\RCDB\DGL\20250910\DGL-S7-1-POS-001.d
**Inj. Vol. (ul)**	1	**Method Path (Acq)**	D:\Projects\2023\Methods\General positive organic analysis method-2 .m
**Position**	P1-a2	**Acq. Time (Local)**	9/10/2025 12:25:01 PM (UTC+08:00)
**MS Type**	QTOF	**Ion Polarity**	Positive
**Instrument**	G6545B	**Version (Acq SW)**	6200 series TOF/6500 series Q-TOF (11.0.221.1)
**Operator**	SYSTEM (SYSTEM)		

Sample Spectra

+ Scan (rt: 9.835 min)

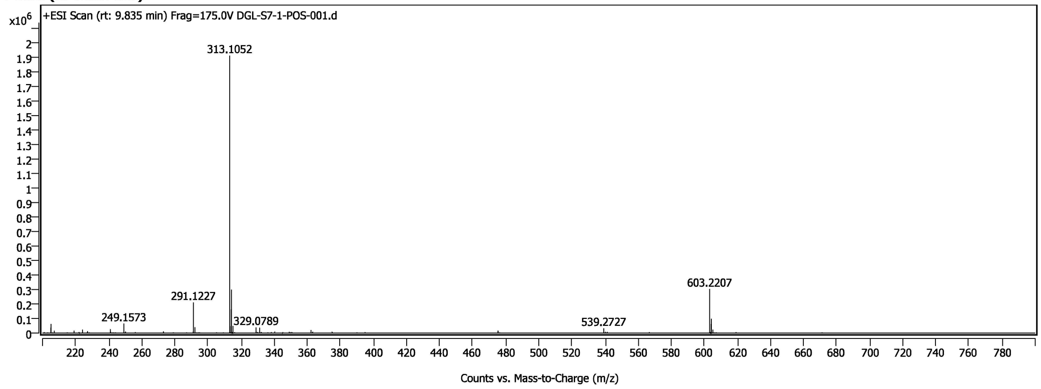

Spectrum Peaks

m/z	z	m/z (Calc)	Ion Species	Formula	Diff (ppm)	Diff (mDa)
291.1227	1	291.1227	(M+H)+	C16 H18 O5	0.04	0.0
292.1261	1	292.1261	(M+H)+	C16 H18 O5	-0.15	0.0

Spectrum Identification Table

Formula	m/z	Mass	Species	Score	Diff (ppm)	Diff (mDa)
C16 H18 O5	291.1227	290.1154	(M+H)+	99.66	-0.01	0.0
C15 H12 N7	291.1227	290.1156	(M+H)+	98.89	0.52	0.1
C17 H14 N4 O	291.1227	290.1155	(M+H)+	93.76	-4.36	-1.3
C14 H16 N3 O4	291.1227	290.1155	(M+H)+	92.09	4.87	1.4

MassHunter Qual 10.0
(End of Report)

MassHunter Qualitative Analysis | Page 1 of 1 | Generated at 1:46 PM on 9/10/2025

Figure S27. HRESIMS spectrum of **3**.

RCDB

#	样品ID	用户名	日期和时间	型号#	起始波长	结束波长
3	S7-1 30ug/ml	DELL	2025/2/20 15:18:21	Evo350	200.00	400.00

峰：:

nm	Abs
219.466	1.472
305.908	0.250

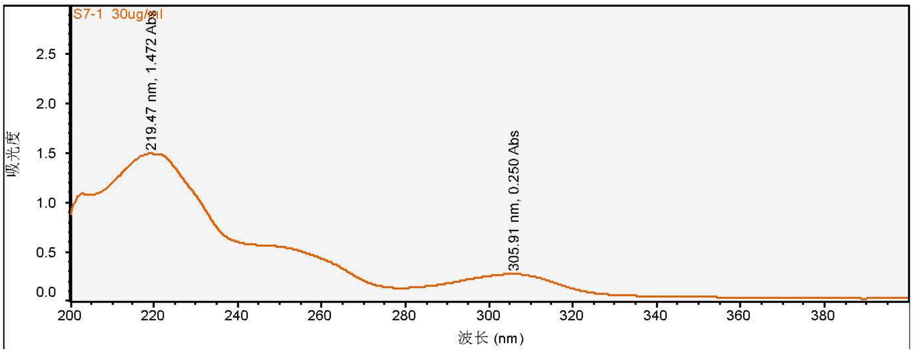

INSIGHT Software

第 1 页，共 1 页

Figure S28. UV spectrum of **3**.

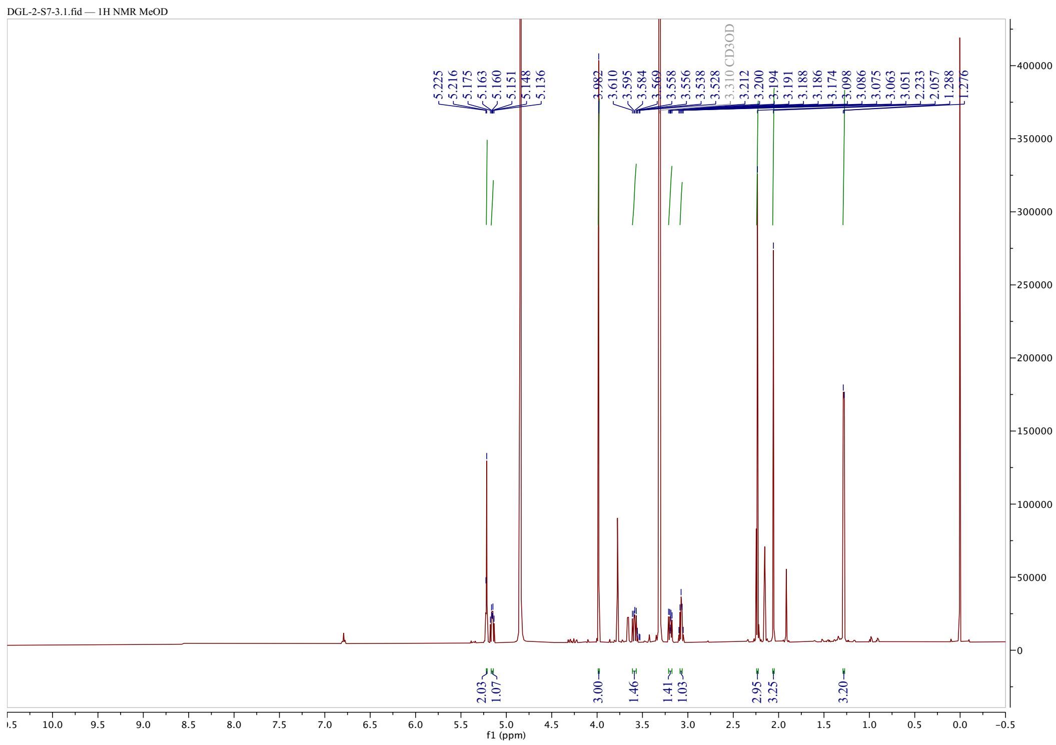

Figure S29. ^{1}H NMR spectrum of **4** (CD_3OD, 600 MHz).

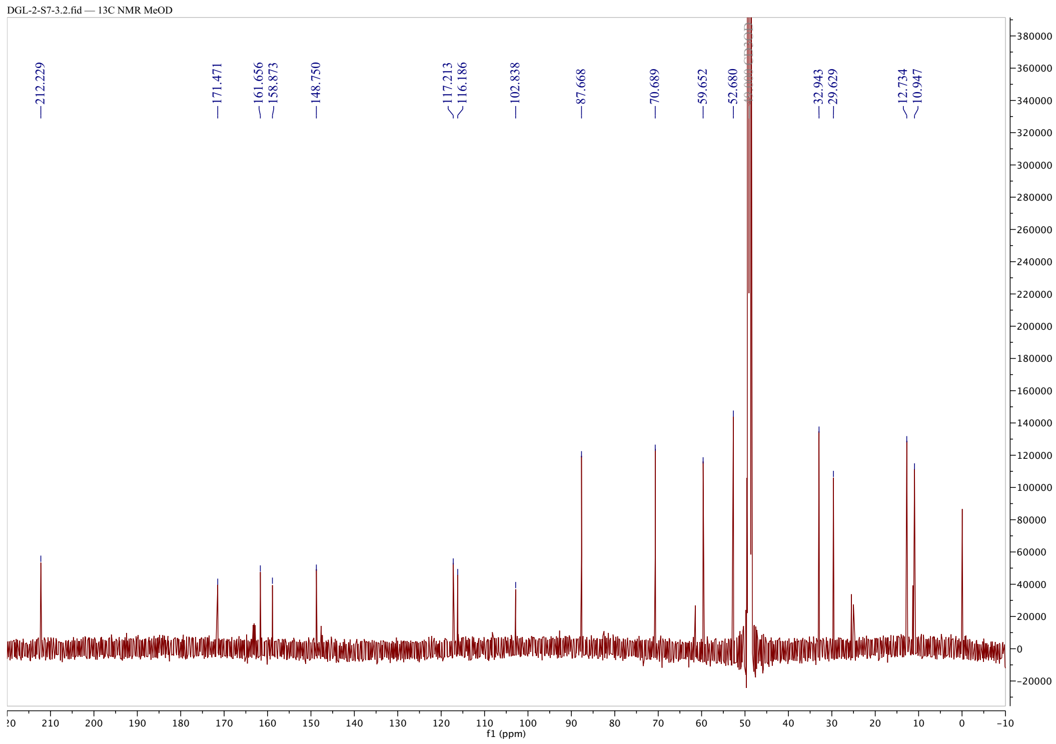

Figure S30. ^{13}C NMR spectrum of **4** (CD_3OD, 151 MHz).

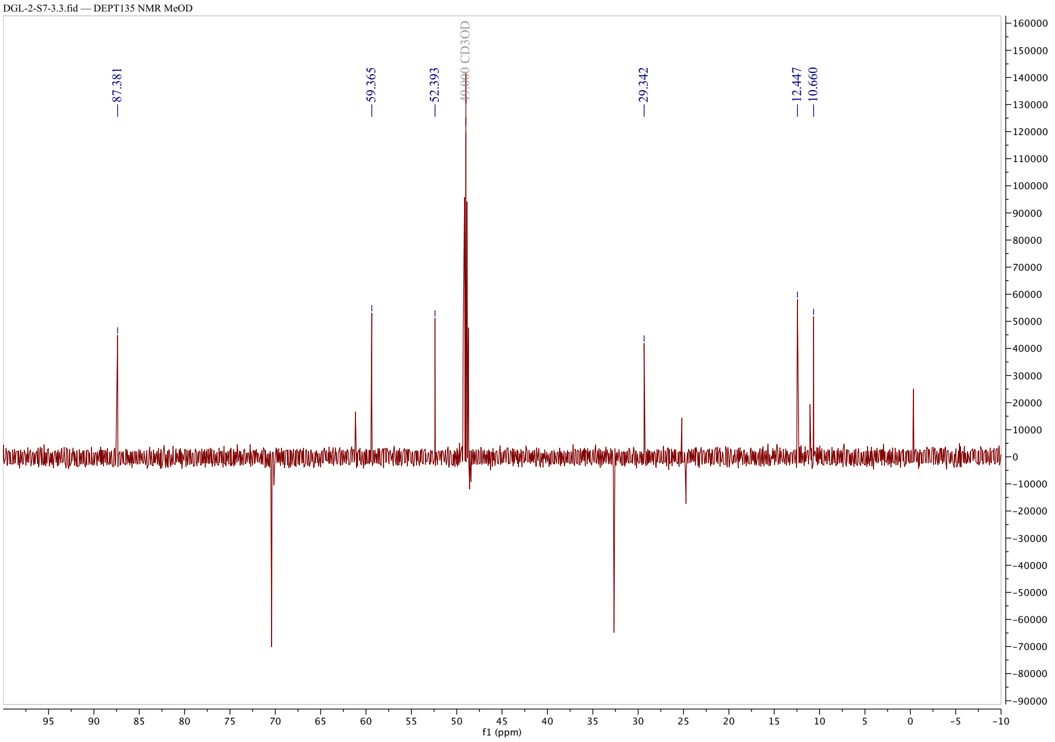

Figure S31. DEPT spectrum of **4** (CD_3OD).

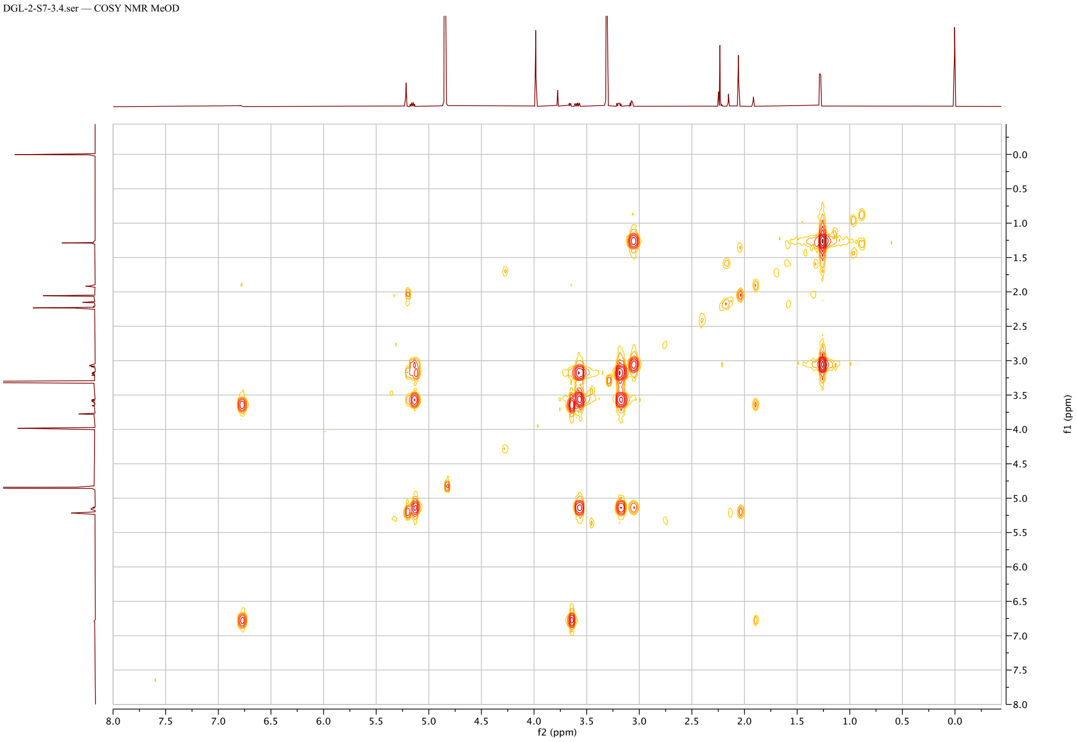

Figure S32. COSY spectrum of **4** (CD_3OD).

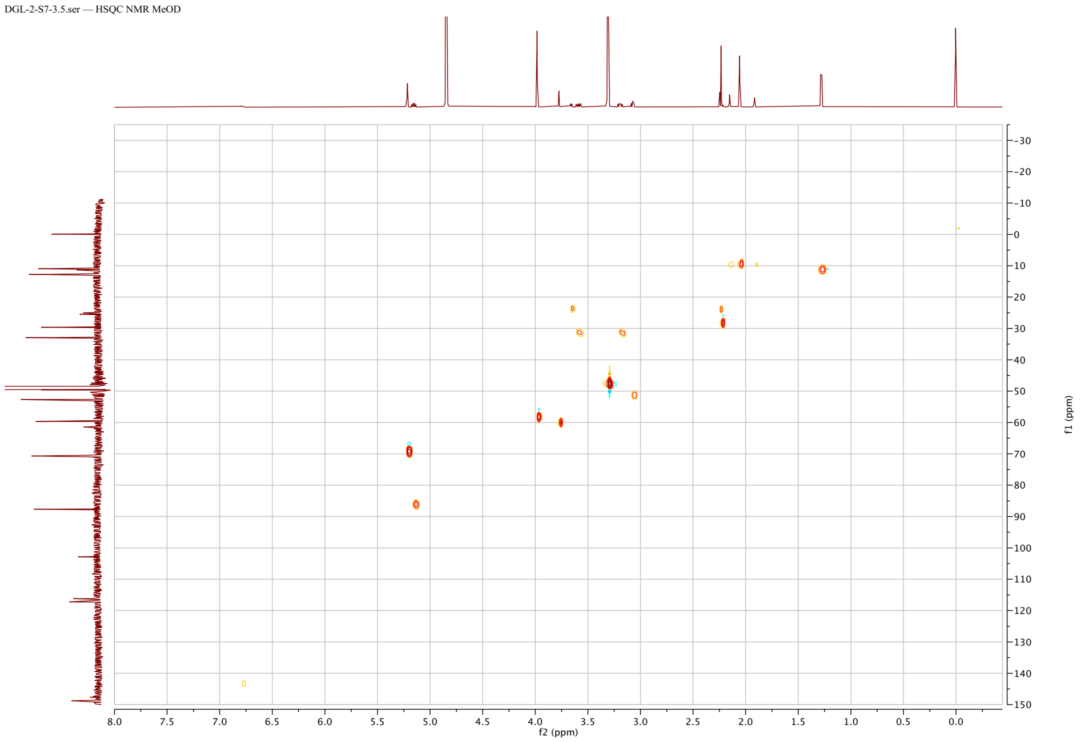

Figure S33. HSQC spectrum of **4** (CD_3OD).

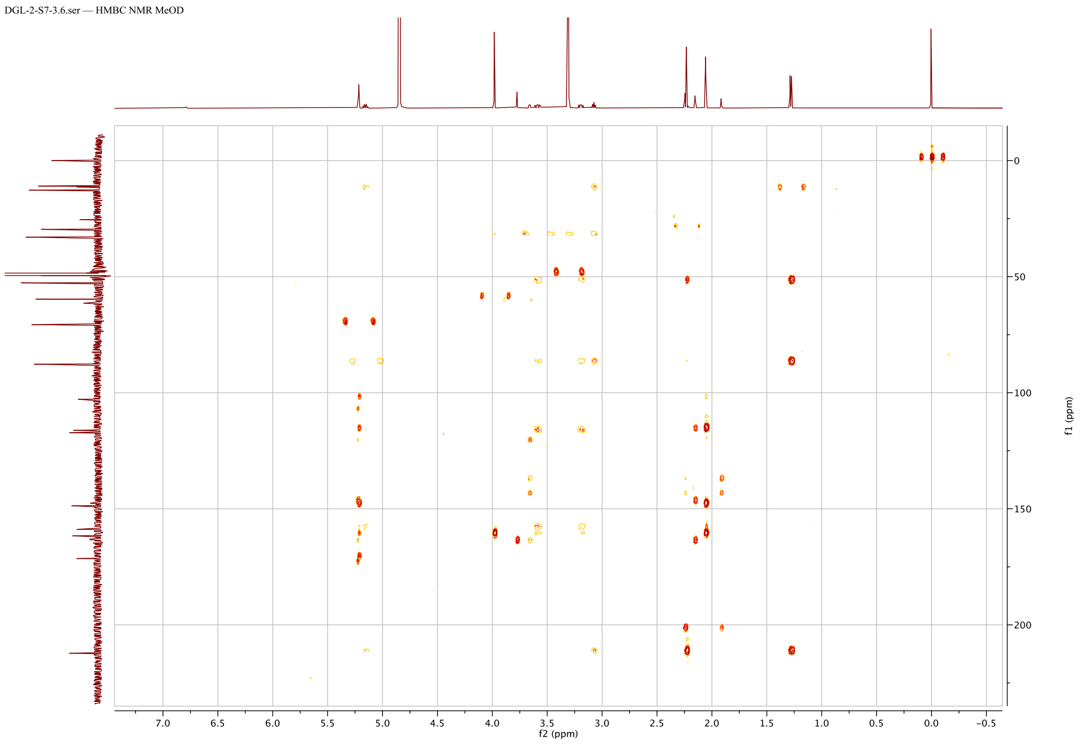

Figure S34. HMBC spectrum of **4** (CD_3OD).

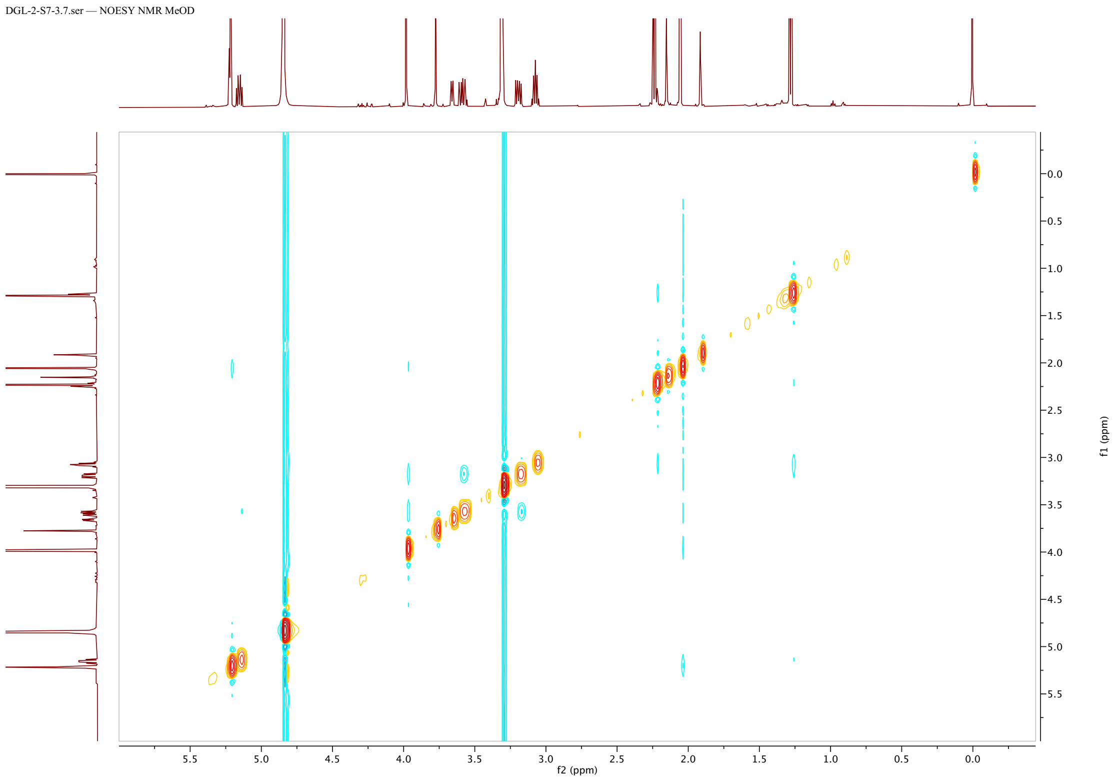

Figure S35. NOESY spectrum of **4** (CD_3OD).

Analysis Report

Sample Information

**Name**	DGL-S7-3-POS-001	**Data File Path**	D:\Projects\2023\Data\RCDB\DGL\DGL-S7-3-POS-001.d
**Inj. Vol. (ul)**	1	**Method Path (Acq)**	D:\Projects\2023\Methods\General positive organic analysis method-2 .m
**Position**	P1-a1	**Acq. Time (Local)**	9/10/2025 10:30:11 AM (UTC+08:00)
**MS Type**	QTOF	**Ion Polarity**	Positive
**Instrument**	G6545B	**Version (Acq SW)**	6200 series TOF/6500 series Q-TOF (11.0.221.1)
**Operator**	SYSTEM (SYSTEM)		

Sample Spectra

+ Scan (rt: 9.890 min)

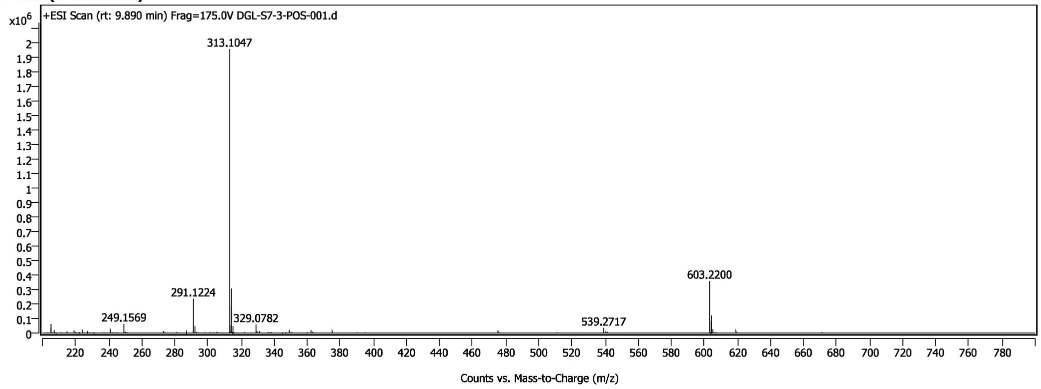

Spectrum Peaks

m/z	z	m/z (Calc)	Ion Species	Formula	Diff (ppm)	Diff (mDa)
291.1224	1	291.1227	(M+H)+	C16 H18 O5	-1.12	-0.3
292.1259	1	292.1261	(M+H)+	C16 H18 O5	-0.85	-0.2

Spectrum Identification Table

Formula	m/z	Mass	Species	Score	Diff (ppm)	Diff (mDa)
C16 H18 O5	291.1224	290.1151	(M+H)+	99.19	-1.10	-0.3
C15 H12 N7	291.1224	290.1153	(M+H)+	98.28	-0.54	-0.2
C14 H16 N3 O4	291.1224	290.1152	(M+H)+	93.94	3.80	1.1
C17 H14 N4 O	291.1224	290.1152	(M+H)+	90.92	-5.44	-1.6

MassHunter Qual 10.0
(End of Report)

MassHunter Qualitative Analysis | Page 1 of 1 | Generated at 12:21 PM on 9/10/2025

Figure S36. HRESIMS spectrum of **4**.

RCDB

样品ID	日期和时间	积分时间 (s)	扫描速度	数据间隔	起始波长	结束波长	带宽	型号#
样品S7-3-15	2025/2/25 13:21:49	0.05	1200.00	1.00	200.00	400.00	0.5 nm	Evo350

峰：：

nm	Abs
220.639	1.361
305.742	0.256

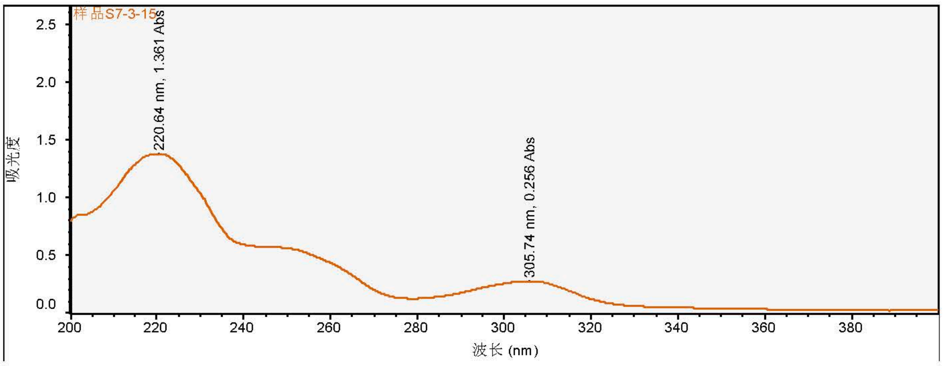

INSIGHT Software 第 1 页，共 1 页

Figure S37. UV spectrum of **4**.

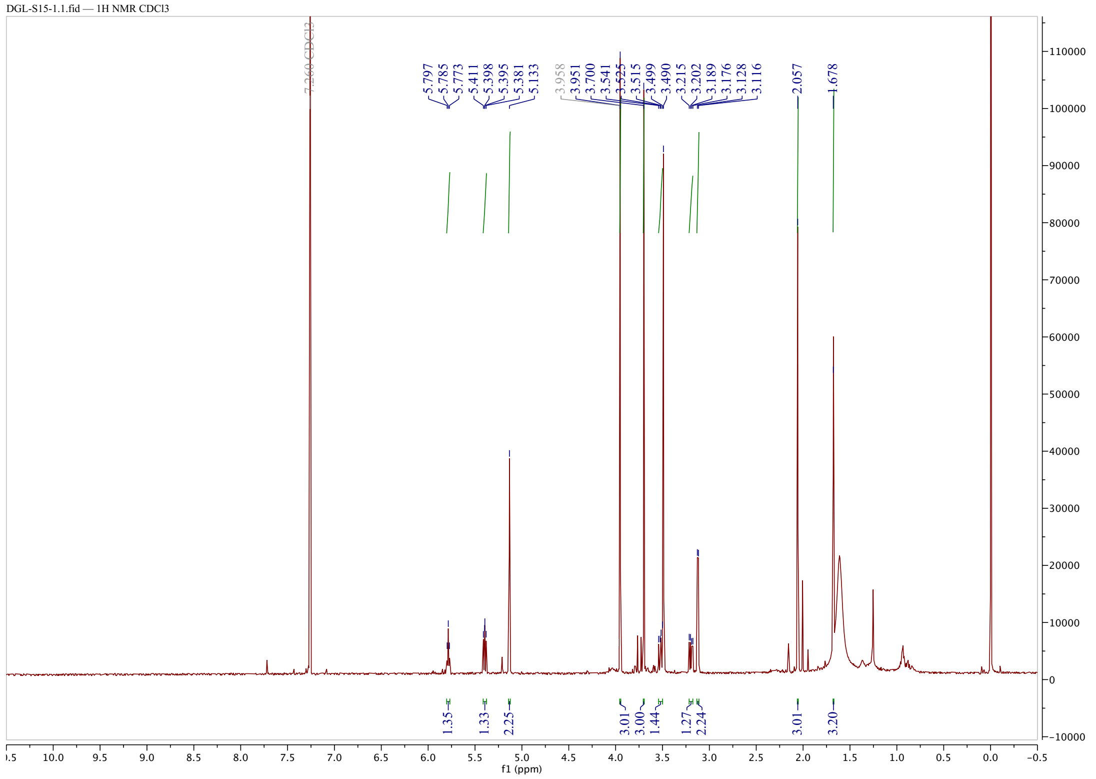

Figure S38. ^{1}H NMR spectrum of **5** ($CDCl_3$, 600 MHz).

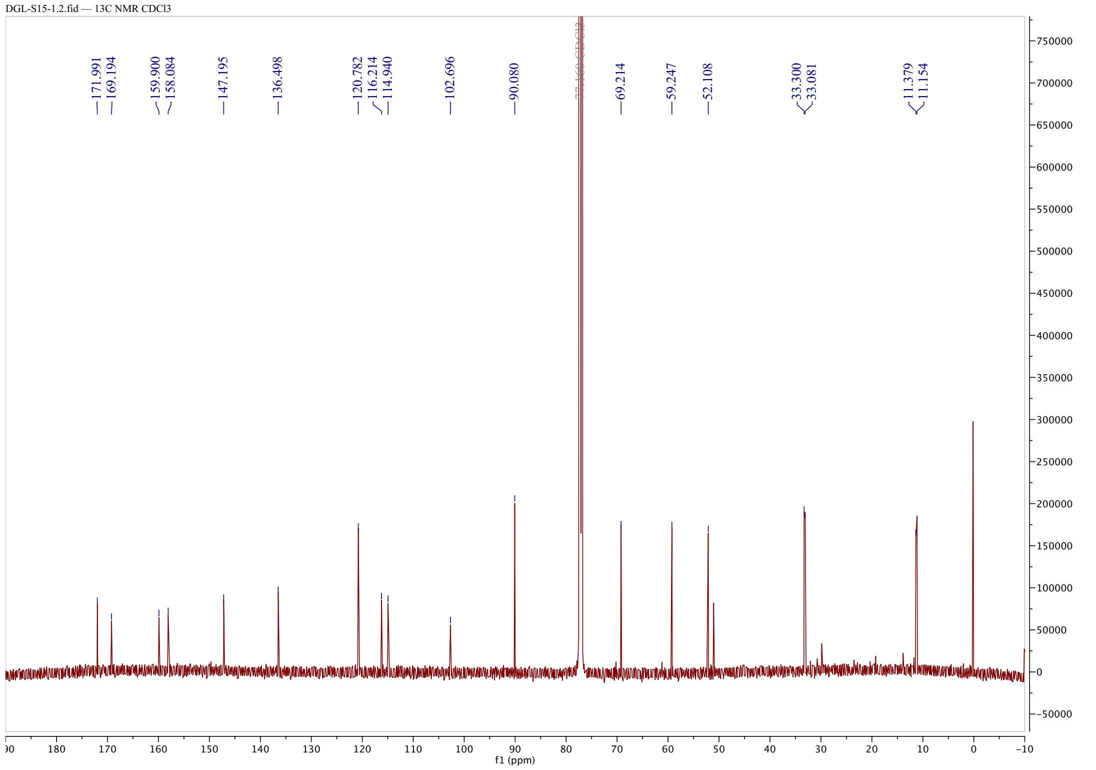

Figure S39. ^{13}C NMR spectrum of **5** ($CDCl_3$, 151 MHz).

DGL-S15-1.3.fid — DEPT135 NMR CDCl3

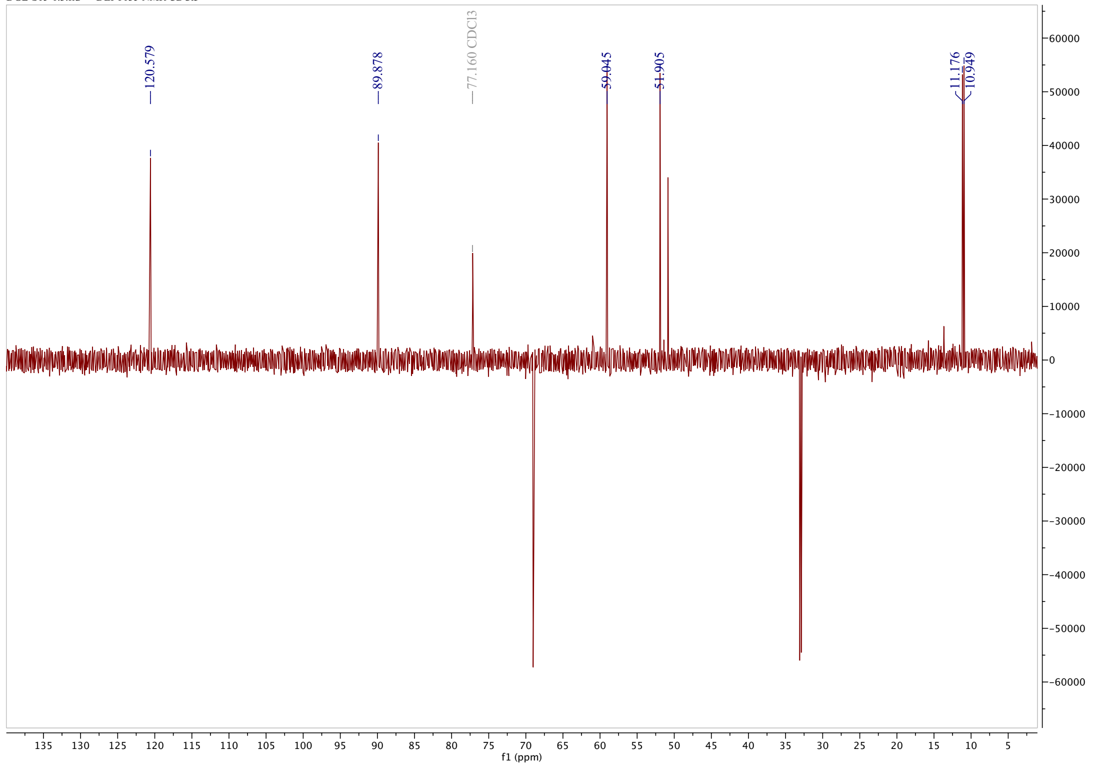

Figure S40. DEPT spectrum of **5** ($CDCl_3$).

DGL-S15-1.4.ser — COSY NMR CDCl3

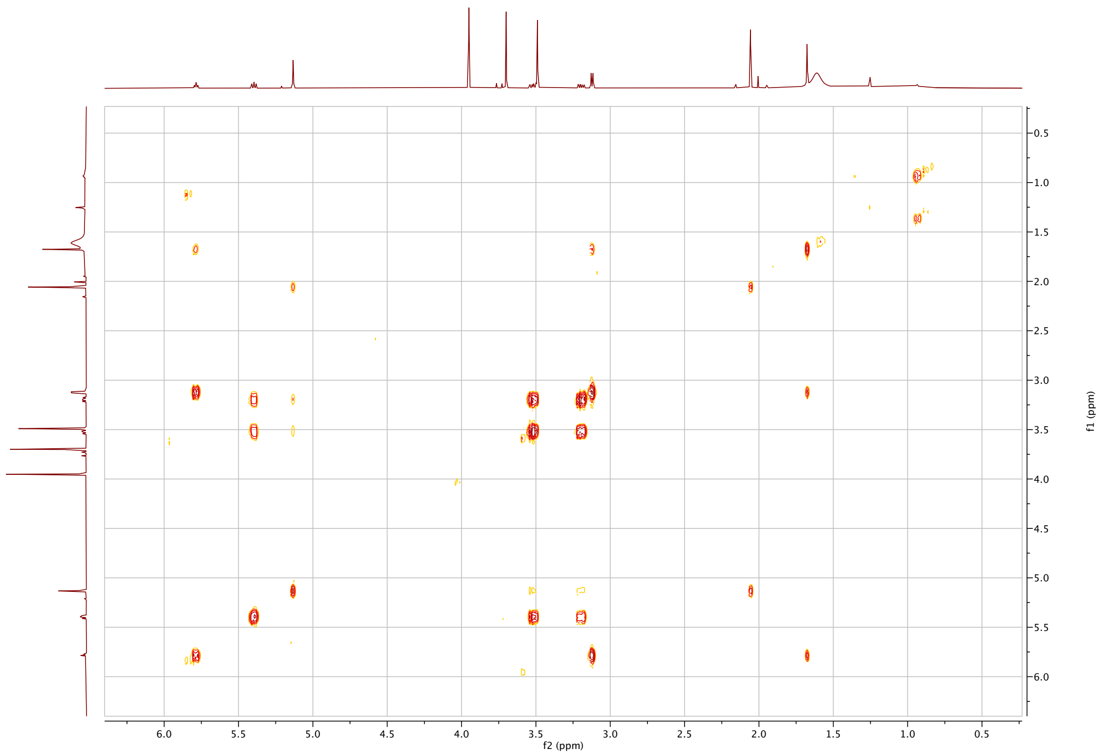

Figure S41. COSY spectrum of **5** ($CDCl_3$).

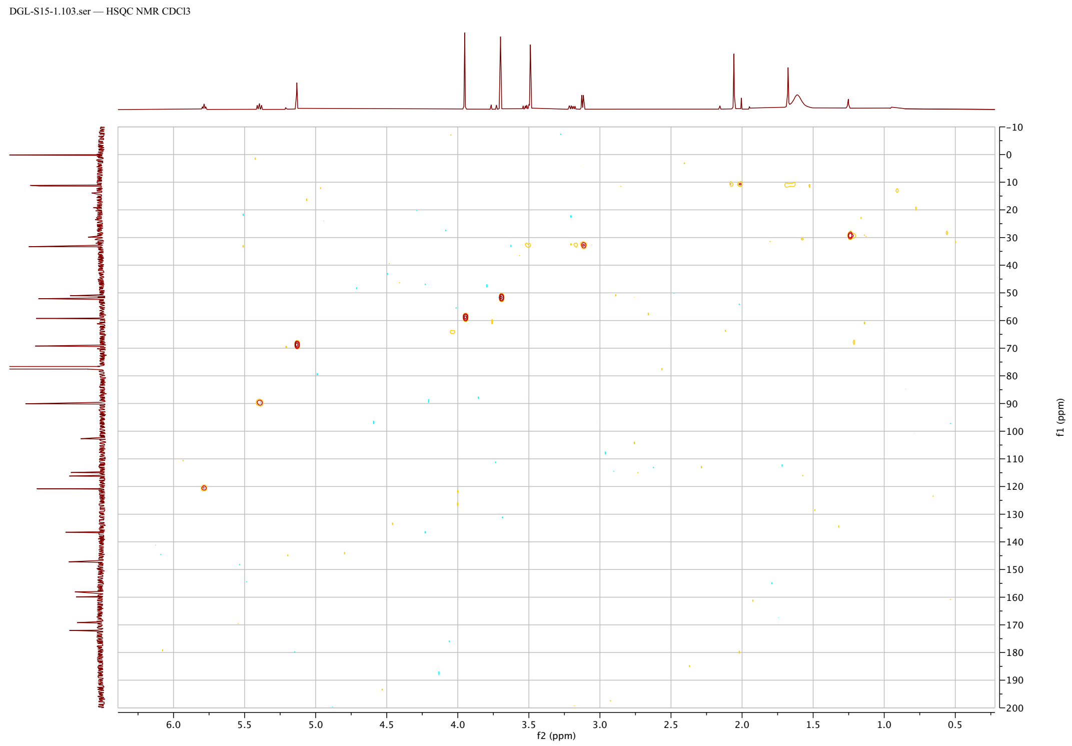

Figure S42. HSQC spectrum of **5** ($CDCl_3$).

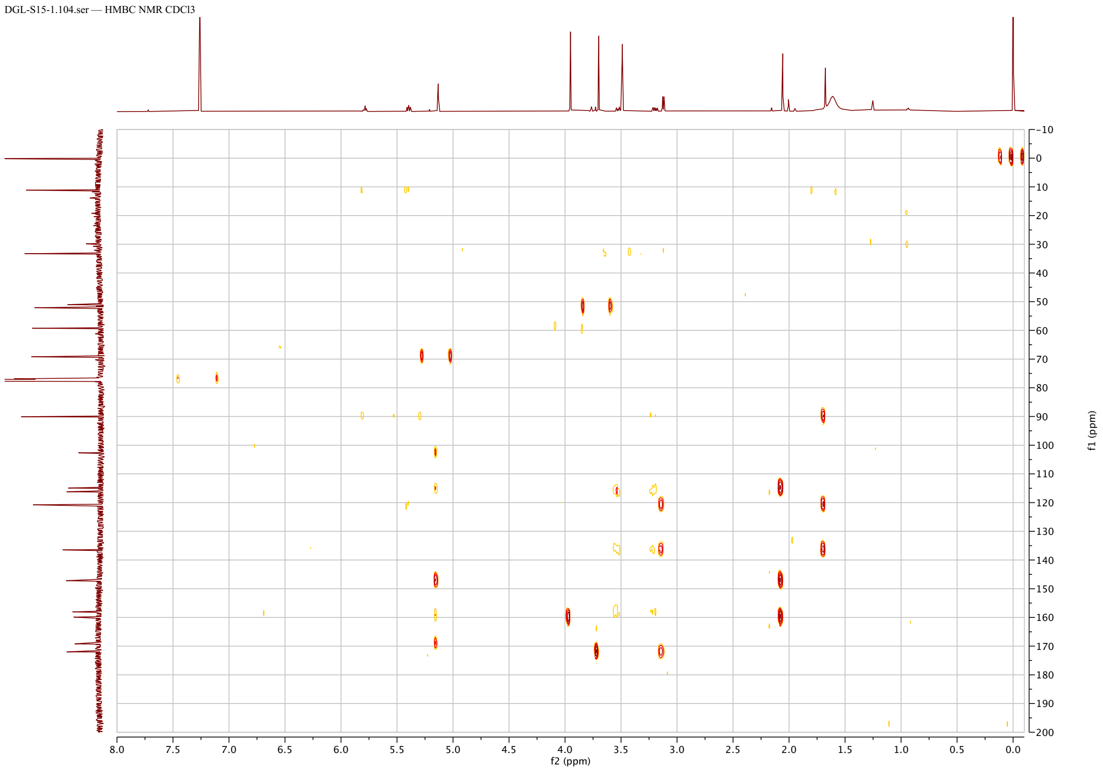

Figure S43. HMBC spectrum of **5** ($CDCl_3$).

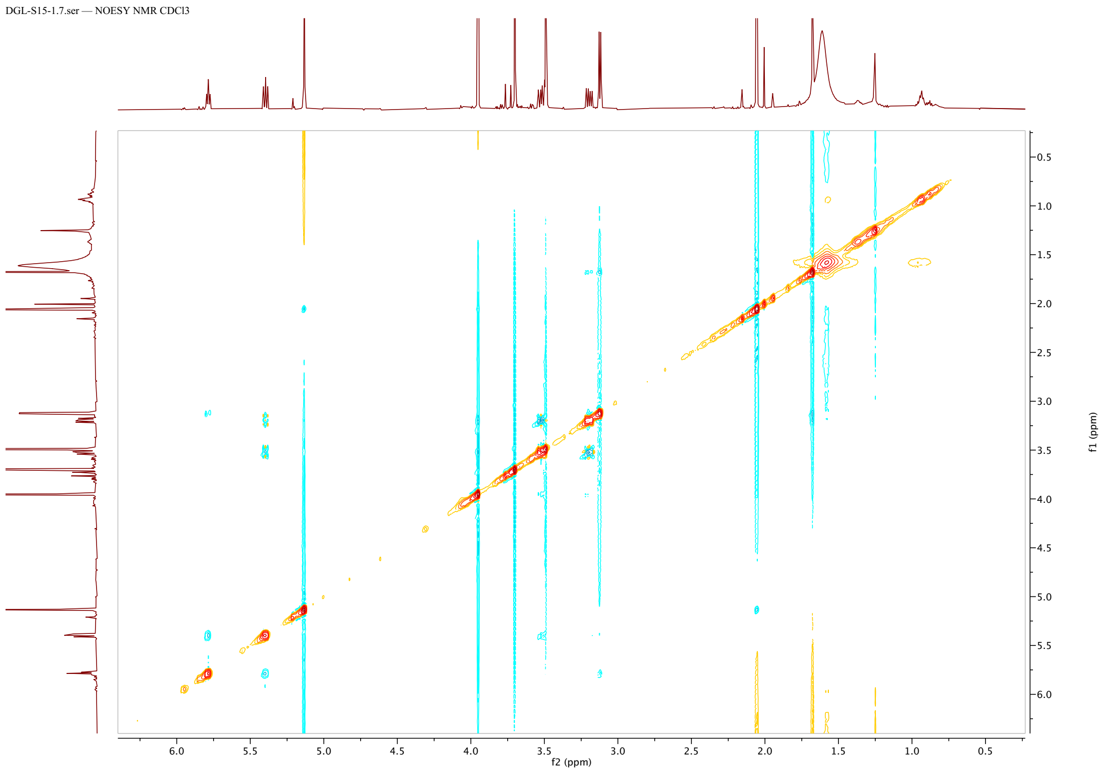

Figure S44. NOESY spectrum of **5** ($CDCl_3$).

Analysis Report

Sample Information

**Name**	DGL-S15-1-POS-02	**Data File Path**	D:\Projects\2023\Data\RCDB\DGL\20250416\DGL-S15-1-POS-02.d
**Inj. Vol. (ul)**	0.5	**Method Path (Acq)**	D:\Projects\2023\Methods\General Positive organic analysis method-3 .m
**Position**	P1-A1	**Acq. Time (Local)**	4/16/2025 3:13:45 PM (UTC+08:00)
**MS Type**	QTOF	**Ion Polarity**	Positive
**Instrument**	G6545B	**Version (Acq SW)**	6200 series TOF/6500 series Q-TOF (11.0.221.1)
**Operator**	SYSTEM (SYSTEM)		

Sample Spectra

+ Scan (rt: 9.466 min)

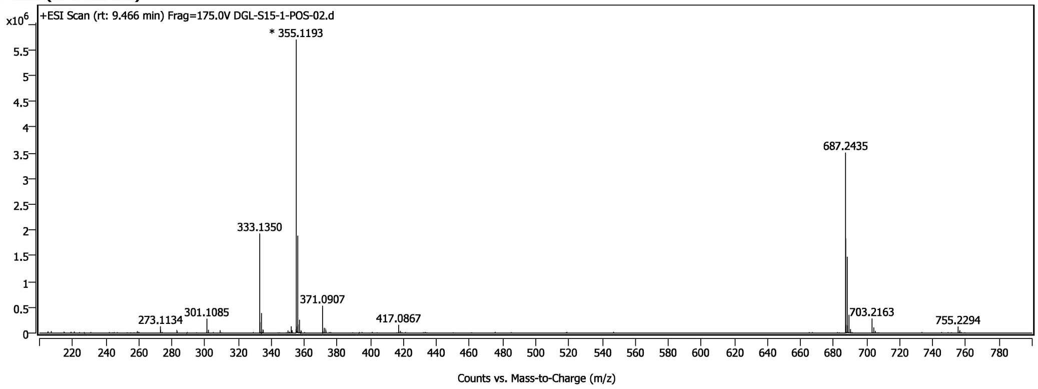

Spectrum Identification Table

Formula	m/z	Mass	Species	Score	Diff (ppm)	Diff (mDa)
C18 H20 O6	333.1350	332.1277	(M+H)+	91.73	5.04	1.7
C12 H16 N10 S	333.1350	332.1281	(M+H)+	91.02	0.23	0.1
C13 H22 N3 O5 S	333.1350	332.1279	(M+H)+	90.39	-0.30	-0.1

MassHunter Qual 10.0
(End of Report)

MassHunter Qualitative Analysis   Page 1 of 1   Generated at 3:36 PM on 4/16/2025

Figure S45. HRESIMS spectrum of **5**.

RCDB

样品ID	日期和时间	积分时间 (s)	扫描速度	数据间隔	起始波长	结束波长	带宽	型号#
S15-1-16	2025/4/15 14:25:41	0.05	1200.00	1.00	200.00	400.00	1.0 nm	Evo350

峰：:

nm	Abs
223.271	1.372
255.778	0.472
307.699	0.268

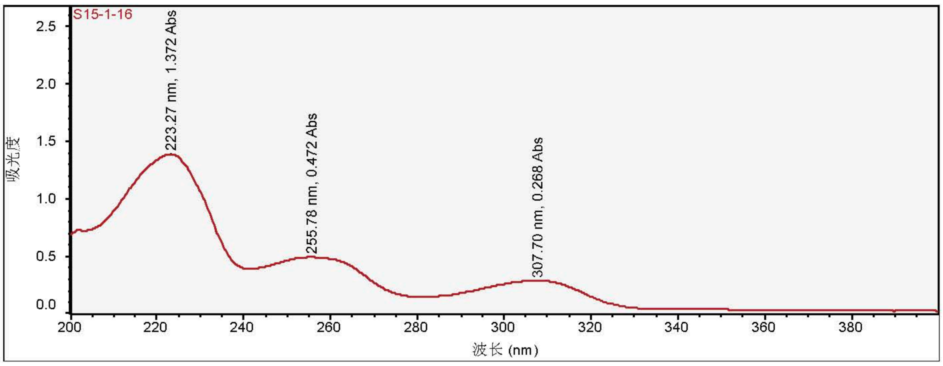

INSIGHT Software 第 1 页，共 1 页

Figure S46. UV spectrum of **5**.

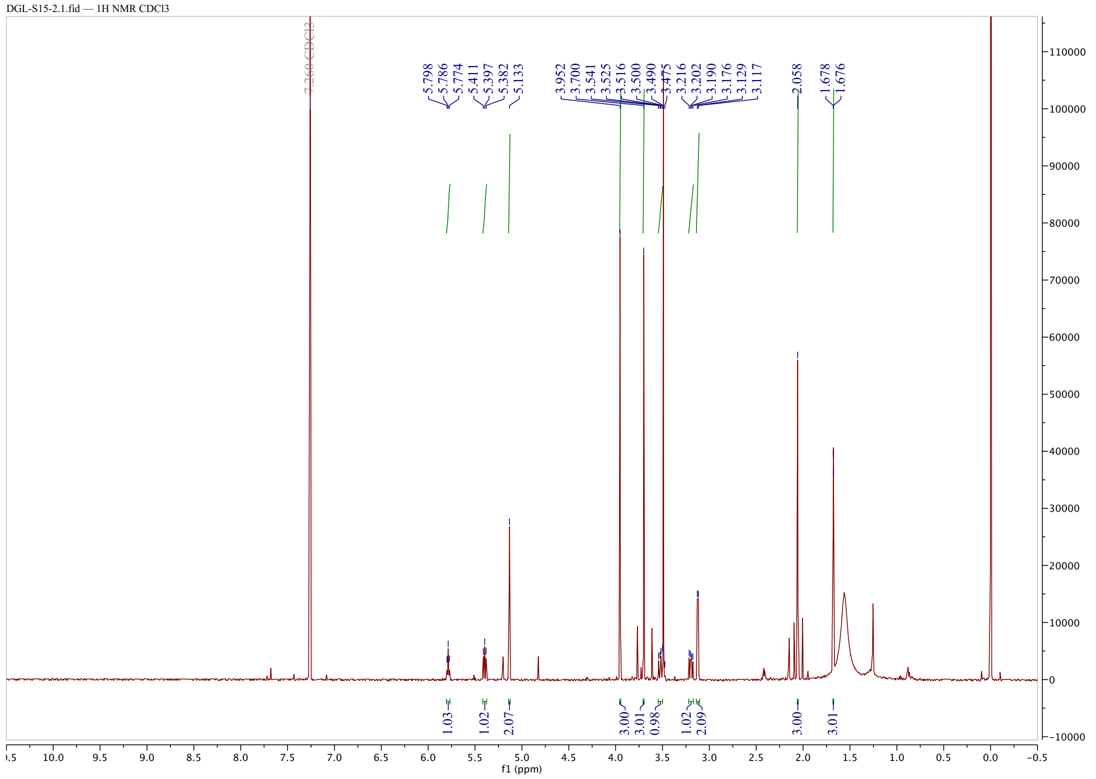

Figure S47. ^{1}H NMR spectrum of **6** ($CDCl_3$, 600 MHz).

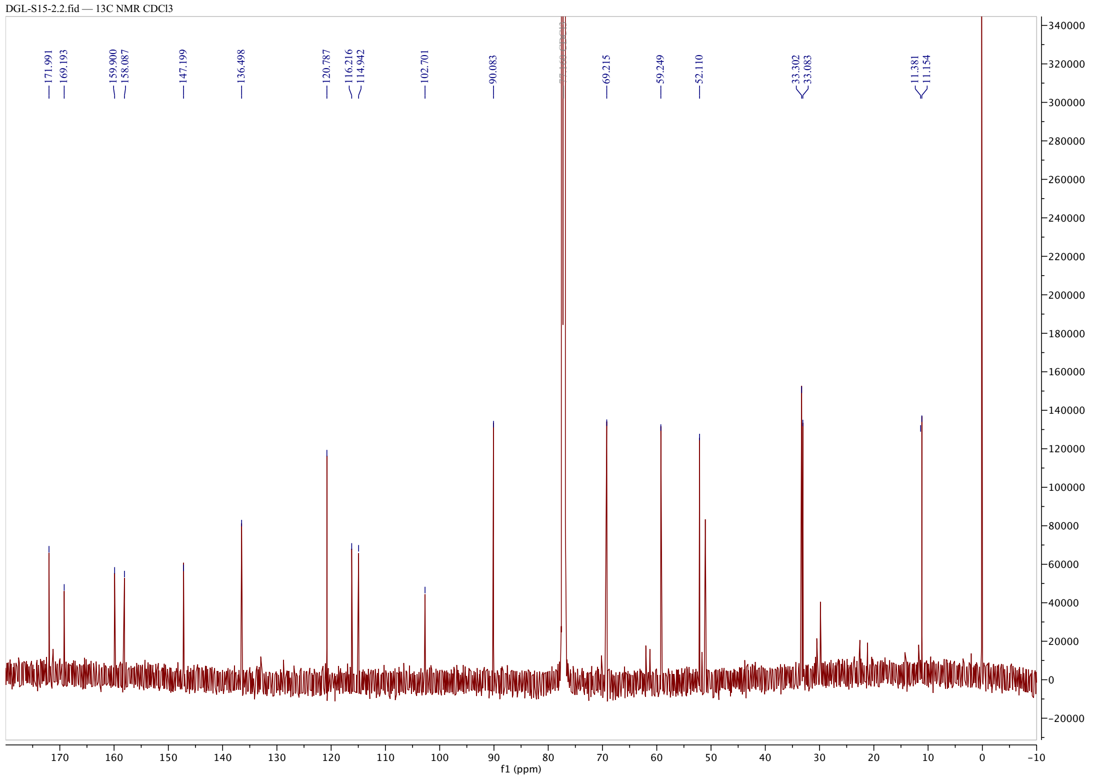

Figure S48. ^{13}C NMR spectrum of **6** ($CDCl_3$, 151 MHz).

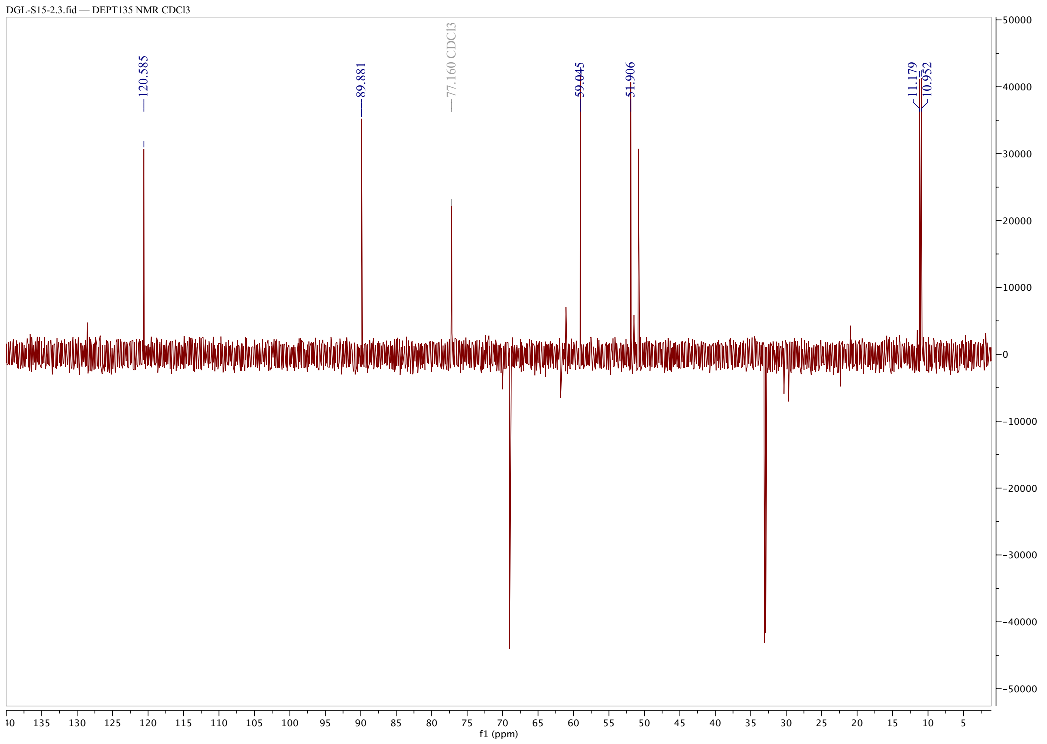

Figure S49. DEPT spectrum of **6** ($CDCl_3$).

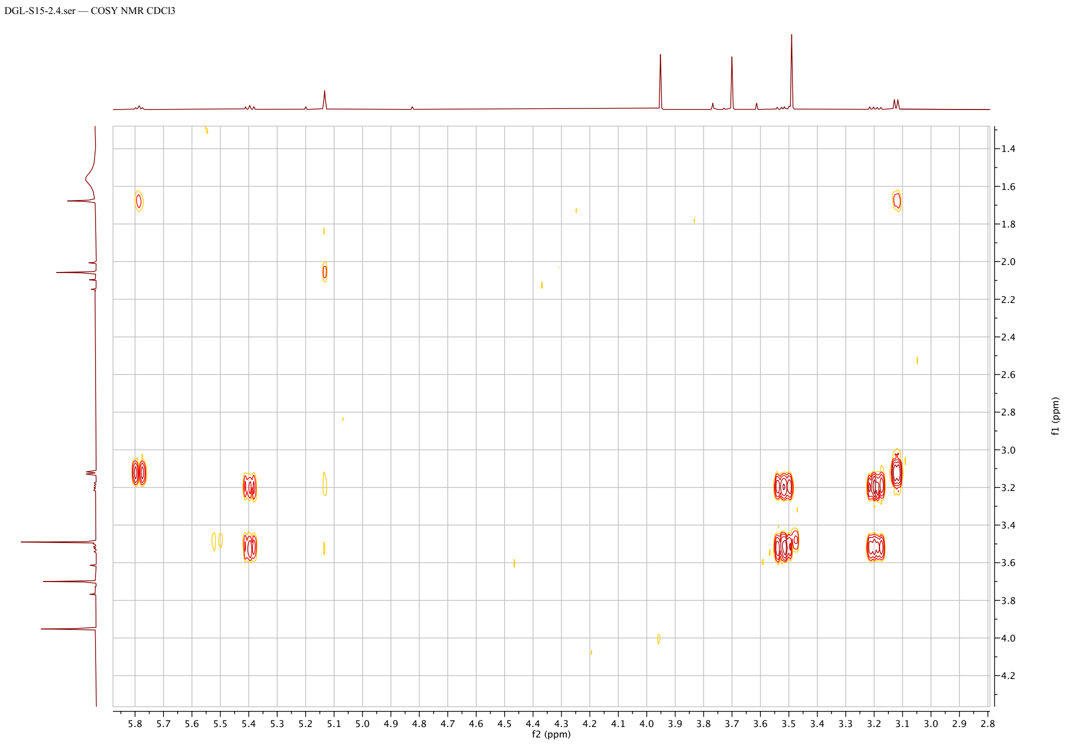

Figure S50. COSY spectrum of **6** ($CDCl_3$).

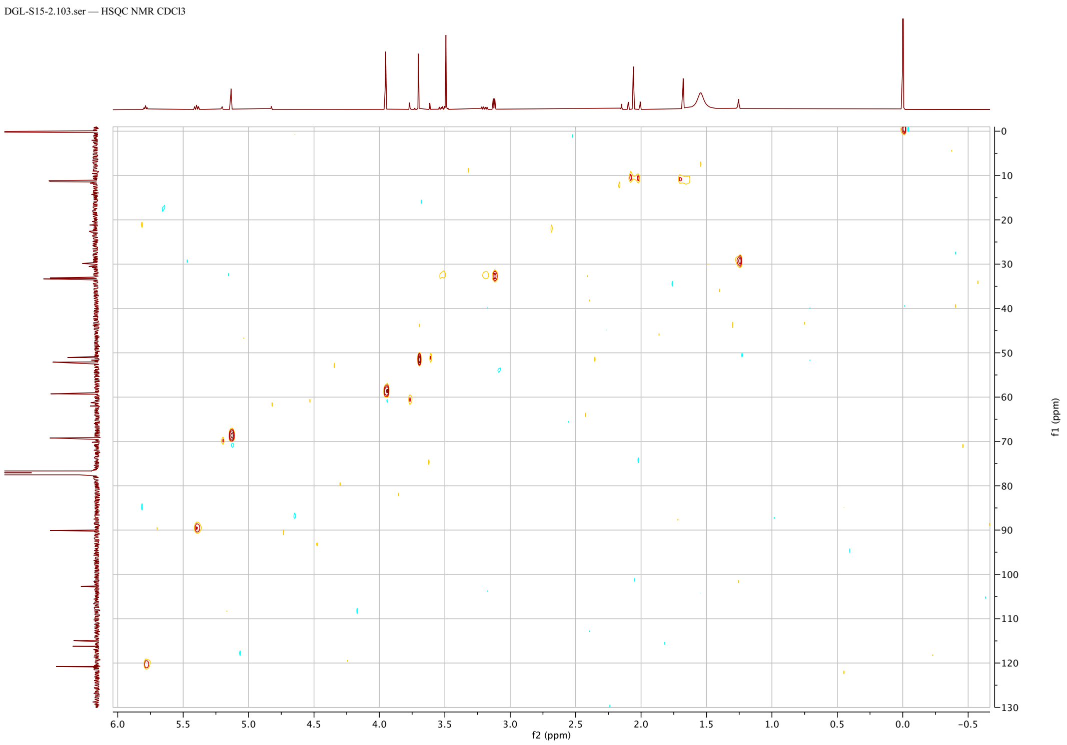

Figure S51. HSQC spectrum of **6** (CDCl₃).

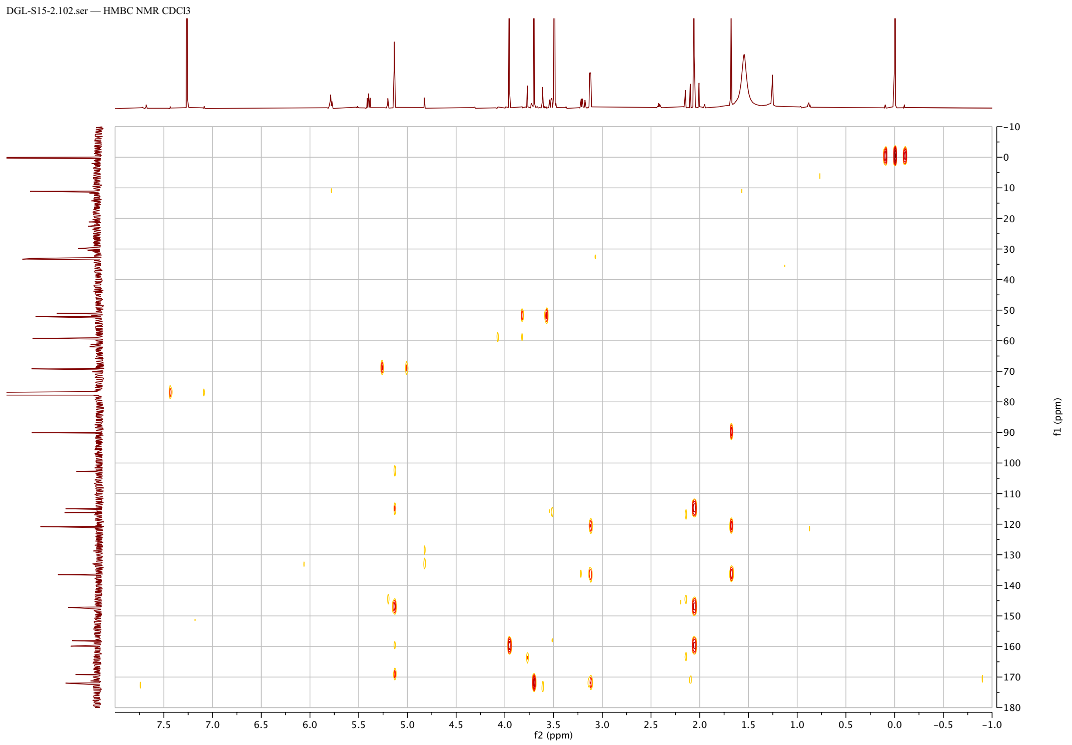

Figure S52. HMBC spectrum of **6** (CDCl₃).

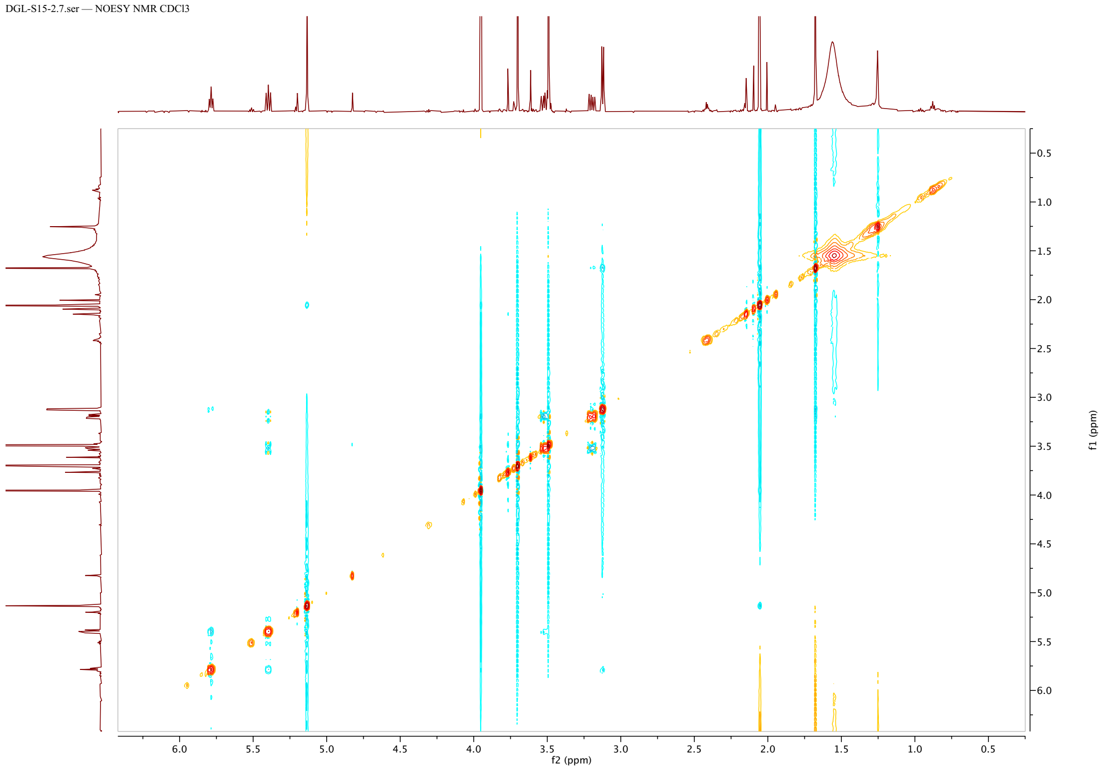

Figure S53. NOESY spectrum of **6** ($CDCl_3$).

Analysis Report

Agilent | Trusted Answers

Sample Information

**Name**	DGL-S15-2-POS-01	**Data File Path**	D:\Projects\2023\Data\RCDB\DGL\20250416\DGL-S15-2-POS-01.d
**Inj. Vol. (ul)**	0.5	**Method Path (Acq)**	D:\Projects\2023\Methods\General Positive organic analysis method-3 .m
**Position**	P1-A1	**Acq. Time (Local)**	4/16/2025 3:38:07 PM (UTC+08:00)
**MS Type**	QTOF	**Ion Polarity**	Positive
**Instrument**	G6545B	**Version (Acq SW)**	6200 series TOF/6500 series Q-TOF (11.0.221.1)
**Operator**	SYSTEM (SYSTEM)		

Sample Spectra

+ Scan (rt: 9.473 min)

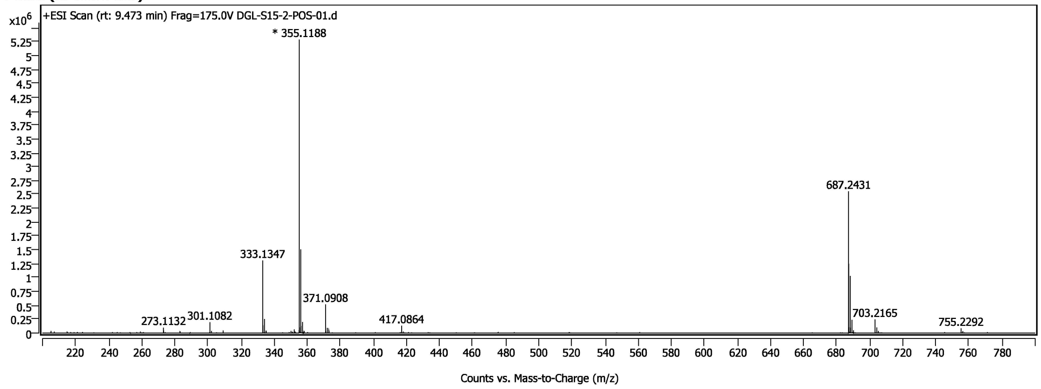

Spectrum Identification Table

Formula	m/z	Mass	Species	Score	Diff (ppm)	Diff (mDa)
C18 H20 O6	333.1347	332.1274	(M+H)+	93.68	4.26	1.4
C12 H16 N10 S	333.1347	332.1278	(M+H)+	91.80	-0.50	-0.2
C13 H22 N3 O5 S	333.1347	332.1277	(M+H)+	90.96	-1.03	-0.3
C21 H18 N O3	333.1347	332.1274	(M+H)+	90.90	-3.78	-1.3

MassHunter Qual 10.0
(End of Report)

Figure S54. HRESIMS spectrum of **6**.

RCDB

样品ID	日期和时间	积分时间 (s)	扫描速度	数据间隔	起始波长	结束波长	带宽	型号#
S15-2-16	2025/4/15 14:30:46	0.05	1200.00	1.00	200.00	400.00	1.0 nm	Evo350

峰：:

nm	Abs
222.197	1.310
253.969	0.426
307.537	0.251

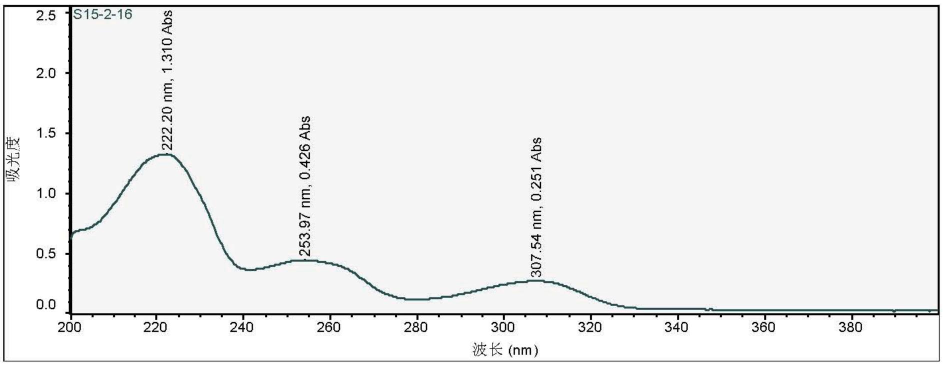

Figure S55. UV spectrum of **6**.

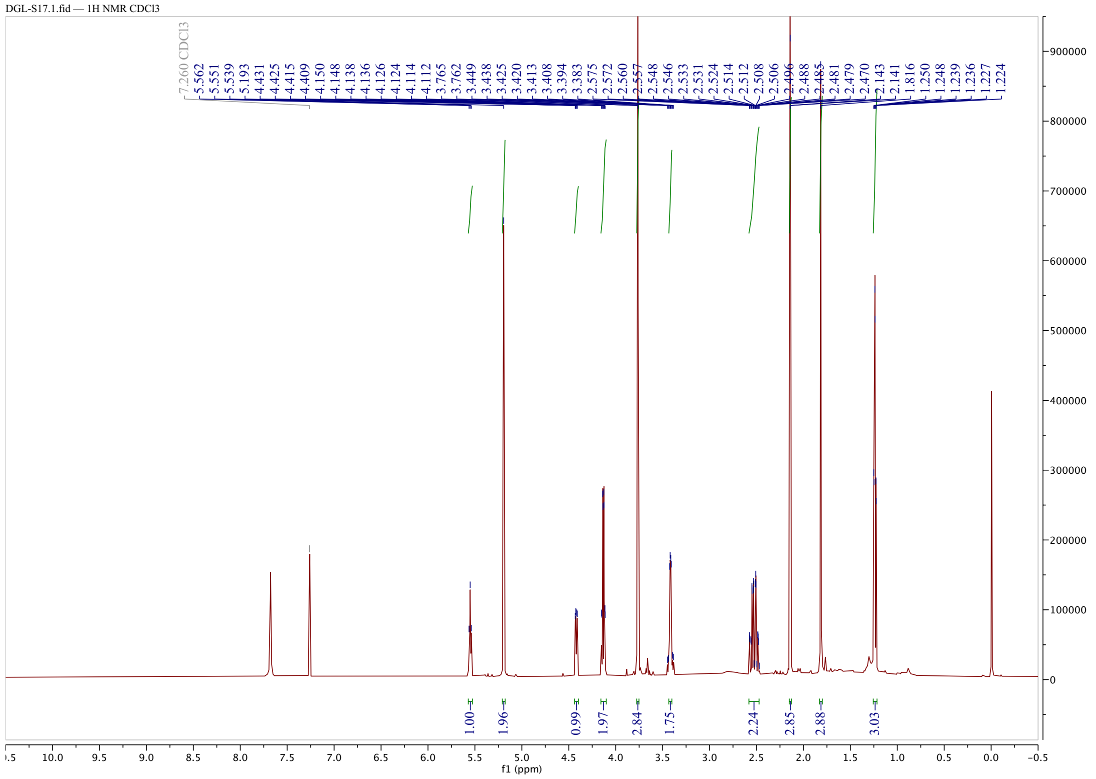

Figure S56. ^{1}H NMR spectrum of **7** ($CDCl_3$, 600 MHz).

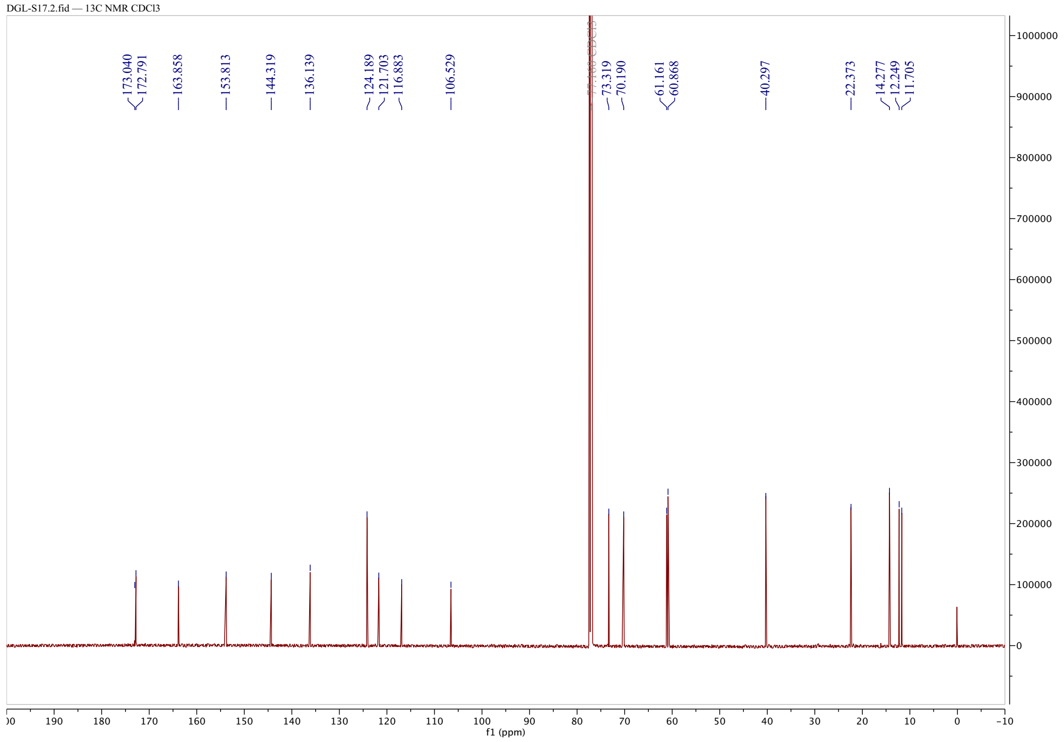

Figure S57. ^{13}C NMR spectrum of **7** ($CDCl_3$, 151 MHz).

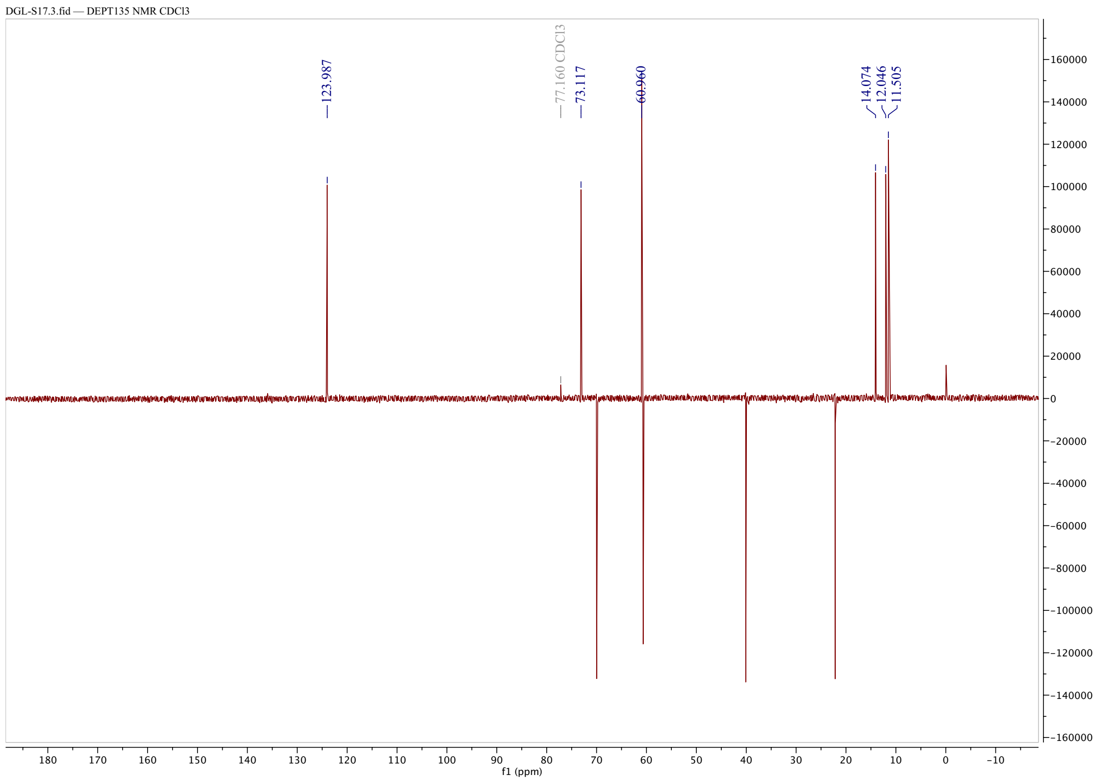

Figure S58. DEPT spectrum of **7** ($CDCl_3$).

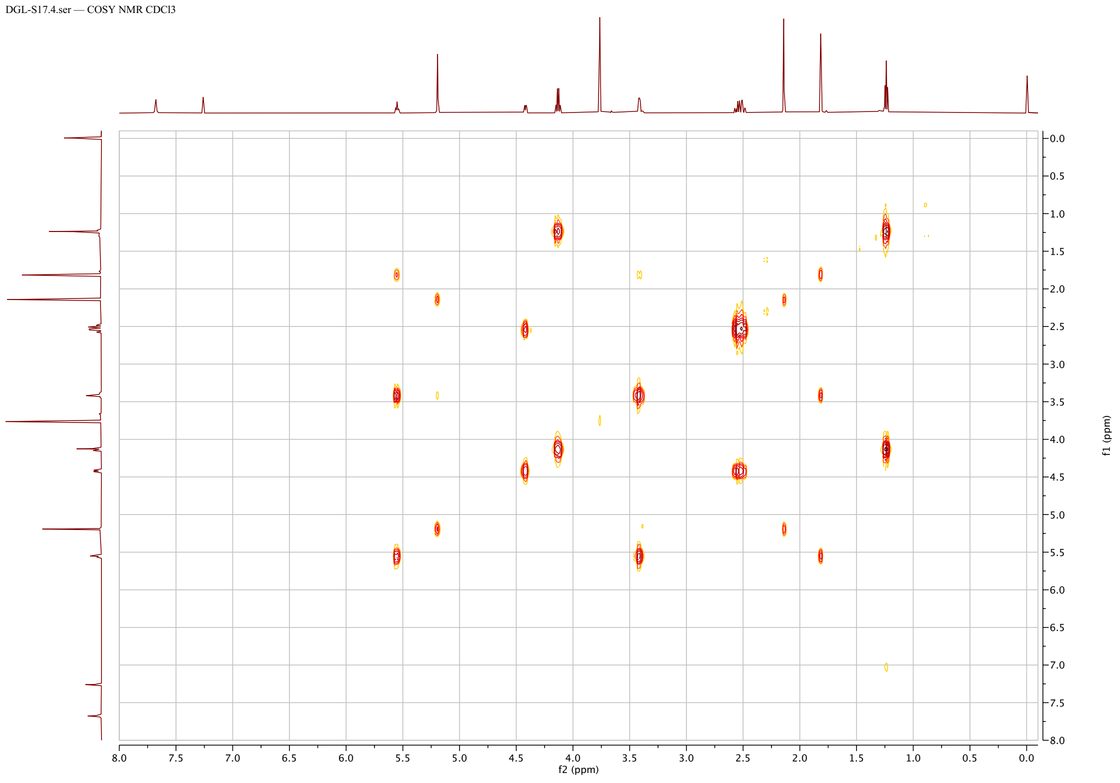

Figure S59. COSY spectrum of **7** ($CDCl_3$).

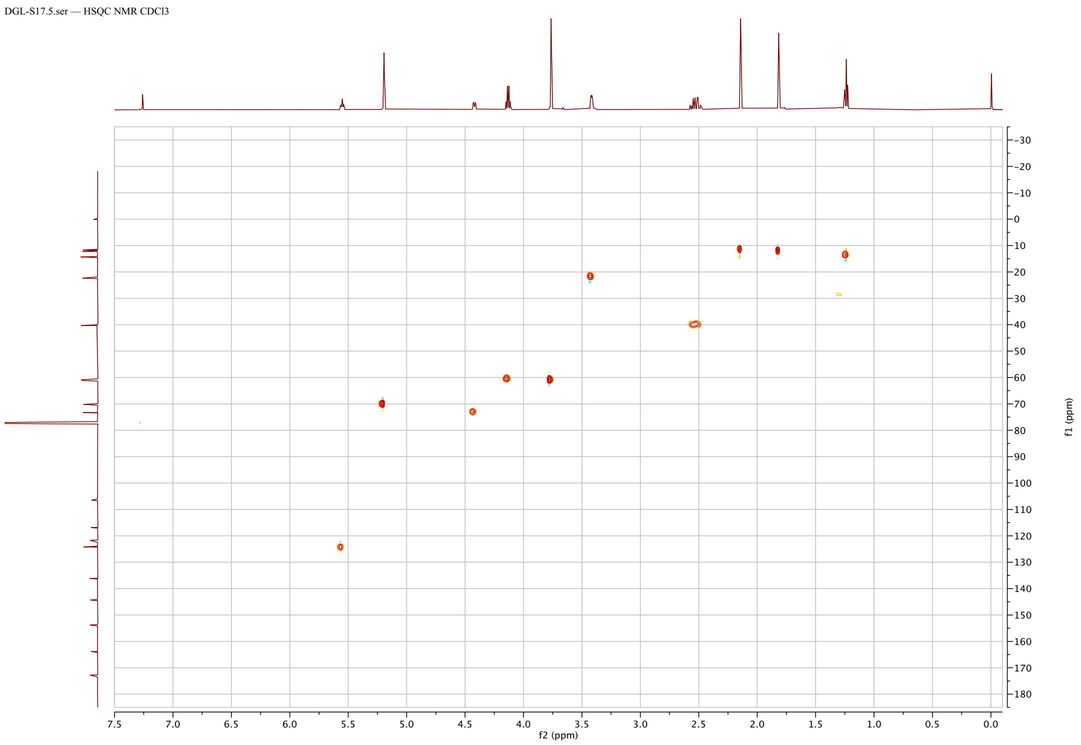

Figure S60. HSQC spectrum of **7** (CDCl3).

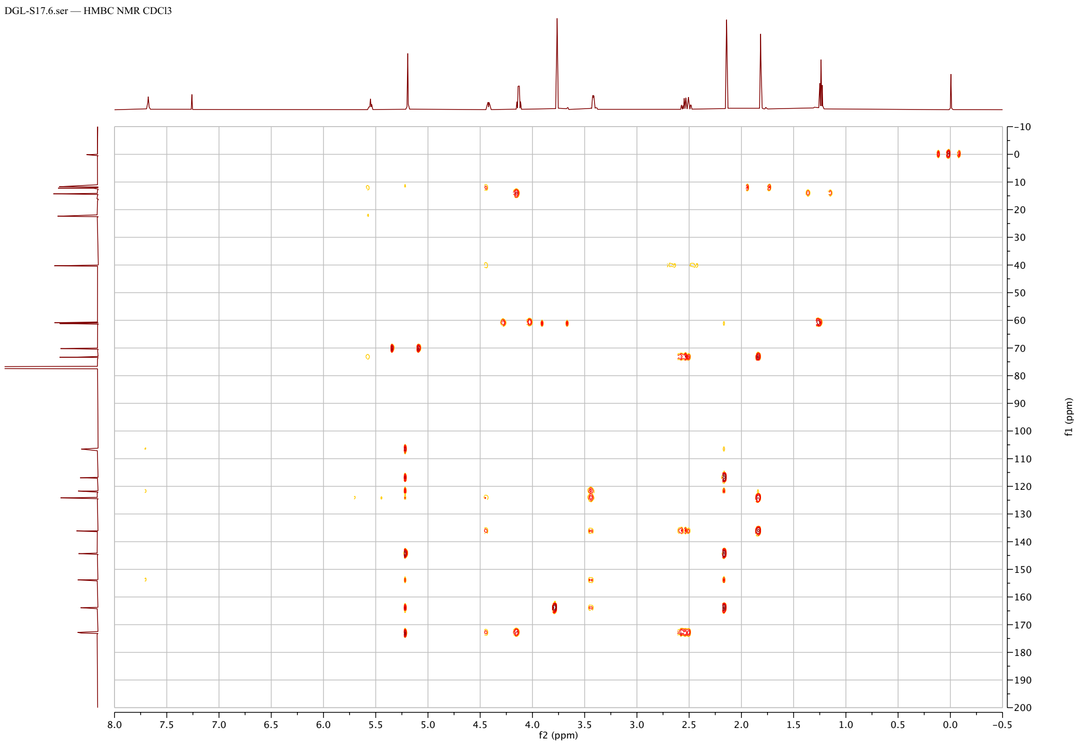

Figure S61. HMBC spectrum of **7** (CDCl3).

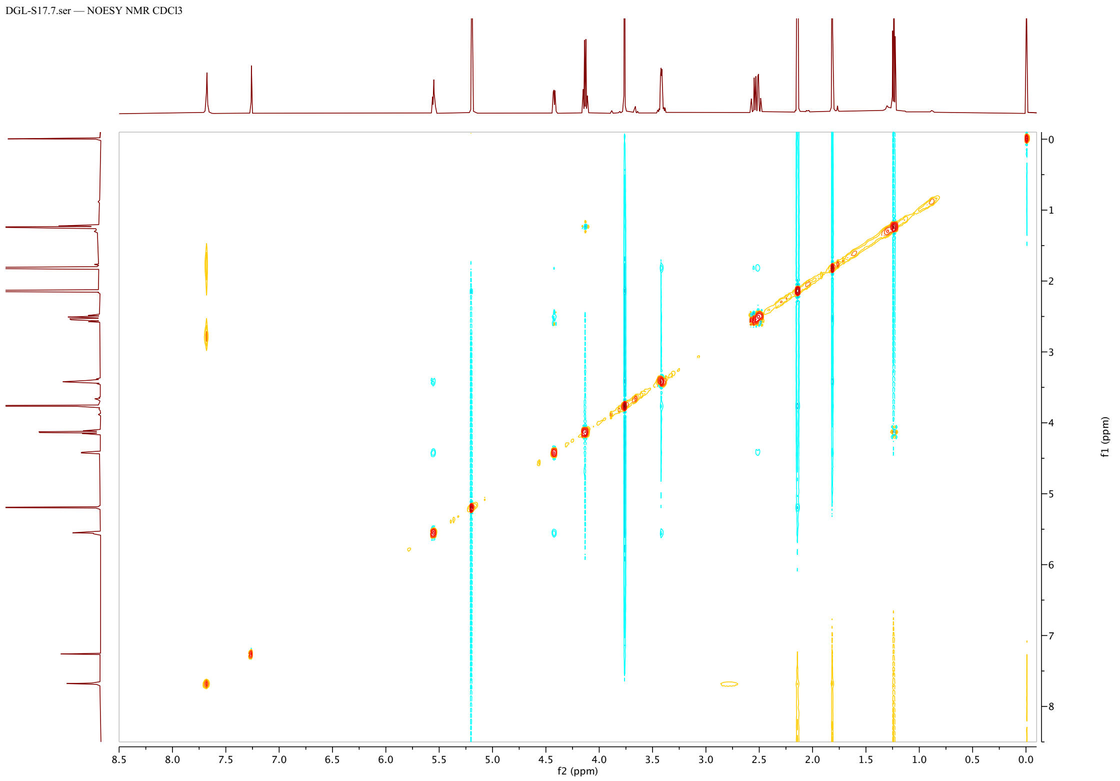

Figure S62. NOESY spectrum of **7** ($CDCl_3$).

Analysis Report

Sample Information

**Name**	DGL-S17-POS-02	**Data File Path**	D:\Projects\2023\Data\RCDB\DGL\20241129\DGL-S17-POS-02.d
**Inj. Vol. (ul)**	1	**Method Path (Acq)**	D:\Projects\2023\Methods\General positive organic analysis method-1 .m
**Position**	P1-A1	**Acq. Time (Local)**	11/29/2024 1:00:20 PM (UTC+08:00)
**MS Type**	QTOF	**Ion Polarity**	Positive
**Instrument**	G6545B	**Version (Acq SW)**	6200 series TOF/6500 series Q-TOF (11.0.221.1)
**Operator**	SYSTEM (SYSTEM)		

Sample Spectra

+ Scan (rt: 9.964 min)

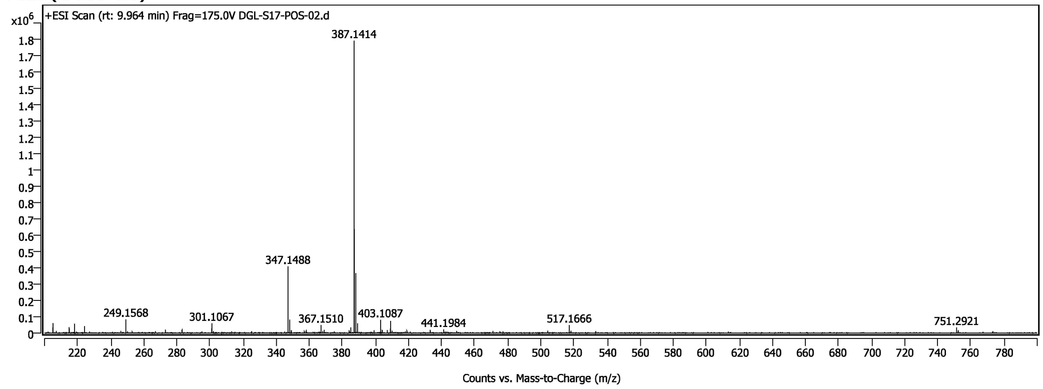

Spectrum Peaks

m/z	z	m/z (Calc)	Ion Species	Formula	Diff (ppm)	Diff (mDa)
387.1414	1	387.1414	(M+Na)+	C19 H24 O7	-0.09	0.0
388.1444	1	388.1448	(M+Na)+	C19 H24 O7	-1.02	-0.4
389.1462	1	389.1472	(M+Na)+	C19 H24 O7	-2.54	-1.0

Spectrum Identification Table

Formula	m/z	Mass	Species	Score	Diff (ppm)	Diff (mDa)
C19 H24 O7	387.1414	364.1521	(M+Na)+	99.62	-0.35	-0.1
C18 H18 N7 O2	387.1414	364.1522	(M+Na)+	98.66	0.06	0.0
C17 H22 N3 O6	387.1414	364.1521	(M+Na)+	95.63	3.53	1.3
C16 H16 N10 O	387.1414	364.1523	(M+Na)+	93.35	3.96	1.4
C20 H20 N4 O3	387.1414	364.1521	(M+Na)+	92.75	-3.83	-1.4

MassHunter Qual 10.0
(End of Report)

Figure S63. HRESIMS spectrum of **7**.

RCDB

#	样品ID	用户名	日期和时间	型号#	起始波长	结束波长
13	S17 20ug/ml	DELL	2025/2/20 15:53:24	Evo350	200.00	400.00

峰：：

nm	Abs
215.392	1.765
249.797	0.390
304.291	0.205

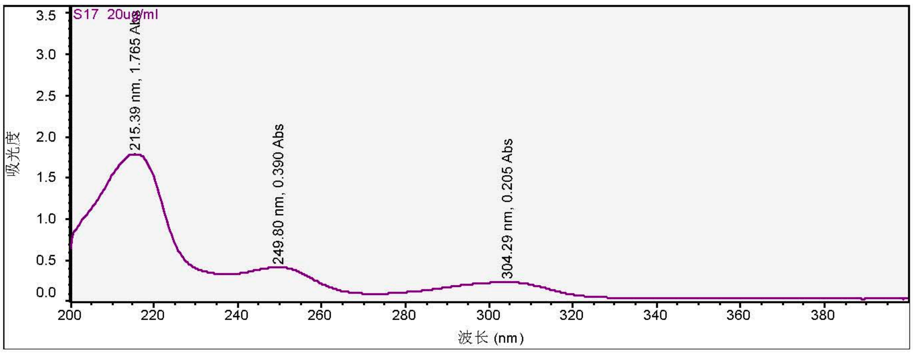

INSIGHT Software 第 1 页，共 1 页

Figure S64. UV spectrum of **7**.

Analysis Report

Sample Information

**Name**	DGL-S17-R1-POS-001	**Data File Path**	D:\Projects\2023\Data\RCDB\DGL\20250528\DGL-S17-R1-POS-001.d
**Inj. Vol. (ul)**	1	**Method Path (Acq)**	D:\Projects\2023\Methods\General Positive organic analysis method-3 .m
**Position**	P1-A1	**Acq. Time (Local)**	5/28/2025 1:13:08 PM (UTC+08:00)
**MS Type**	QTOF	**Ion Polarity**	Positive
**Instrument**	G6545B	**Version (Acq SW)**	6200 series TOF/6500 series Q-TOF (11.0.221.1)
**Operator**	SYSTEM (SYSTEM)		

Sample Spectra

+ Scan (rt: 20.191 min)

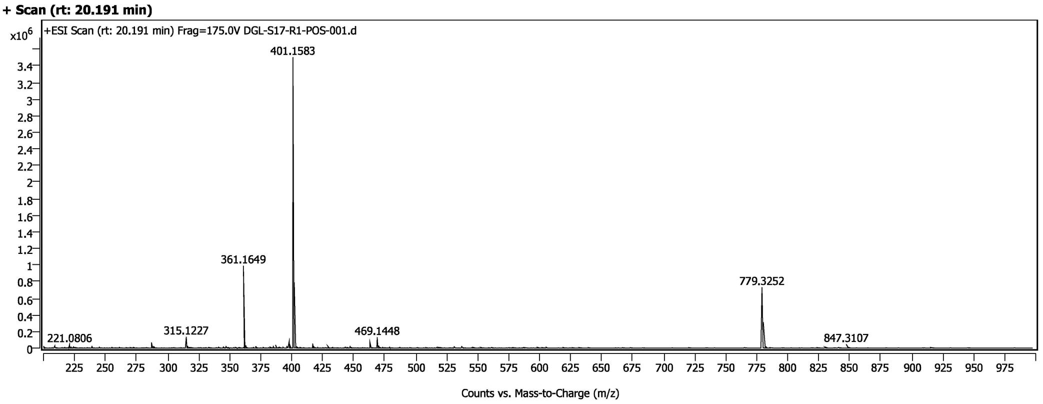

Spectrum Identification Table

Formula	m/z	Mass	Species	Score	Diff (ppm)	Diff (mDa)
C20 H26 O7	401.1583	378.1689	(M+Na)+	96.27	2.80	1.1
C19 H20 N7 O2	401.1583	378.1691	(M+Na)+	95.91	3.21	1.2
C14 H22 N10 O S	401.1583	378.1694	(M+Na)+	91.70	-1.24	-0.5
C23 H24 N O4	401.1583	378.1689	(M+Na)+	91.42	-4.26	-1.6

MassHunter Qual 10.0
(End of Report)

Figure S65. HRESIMS spectrum of **7R**.

Analysis Report

Sample Information

**Name**	DGL-S17-R-POS-001	**Data File Path**	D:\Projects\2023\Data\RCDB\DGL\20250606\DGL-S17-R-POS-001.d
**Inj. Vol. (ul)**	1	**Method Path (Acq)**	D:\Projects\2023\Methods\General Positive organic analysis method-3 .m
**Position**	P1-A1	**Acq. Time (Local)**	6/6/2025 10:50:48 AM (UTC+08:00)
**MS Type**	QTOF	**Ion Polarity**	Positive
**Instrument**	G6545B	**Version (Acq SW)**	6200 series TOF/6500 series Q-TOF (11.0.221.1)
**Operator**	SYSTEM (SYSTEM)		

Sample Spectra

+ Scan (rt: 28.201 min)

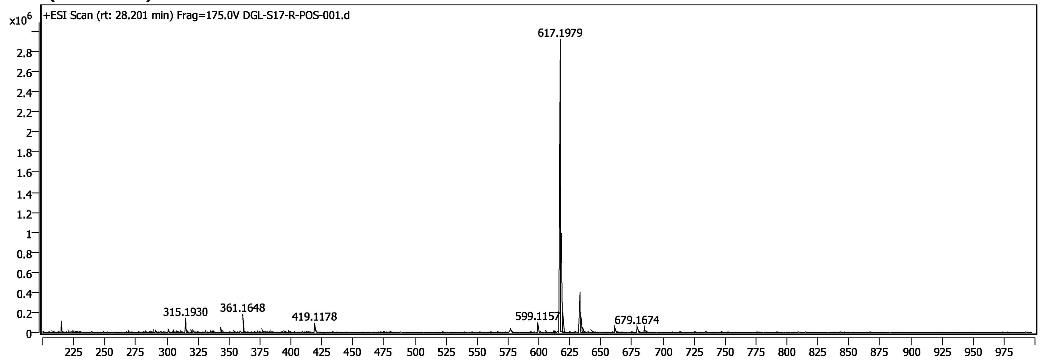

Spectrum Peaks

m/z	z	m/z (Calc)	Ion Species	Formula	Diff (ppm)	Diff (mDa)
617.1979	1	617.1969	(M+Na)+	C30 H33 F3 O9	1.58	1.0
618.2006	1	618.2003	(M+Na)+	C30 H33 F3 O9	0.56	0.3
619.2032	1	619.2030	(M+Na)+	C30 H33 F3 O9	0.28	0.2
620.2056	1	620.2057	(M+Na)+	C30 H33 F3 O9	-0.28	-0.2

Spectrum Identification Table

Formula	m/z	Mass	Species	Score	Diff (ppm)	Diff (mDa)
C30 H33 F3 O9	617.1979	594.2084	(M+Na)+	98.63	1.29	0.8
C27 H34 F4 O10	617.1979	594.2084	(M+Na)+	97.44	-0.63	-0.4
C32 H34 O11	617.1979	594.2084	(M+Na)+	94.80	-2.83	-1.7
C33 H32 F2 O8	617.1979	594.2084	(M+Na)+	93.35	3.20	1.9
C33 H35 F O7 S	617.1979	594.2087	(M+Na)+	92.91	-0.13	-0.1

MassHunter Qual 10.0
(End of Report)

MassHunter Qualitative Analysis   Page 1 of 1   Generated at 3:54 PM on 9/1/2025

Figure S66. HRESIMS spectrum of **7Ra**.

Analysis Report

Sample Information

**Name**	DGL-S17-S-POS-001	**Data File Path**	D:\Projects\2023\Data\RCDB\DGL\20250606\DGL-S17-S-POS-001.d
**Inj. Vol. (ul)**	1	**Method Path (Acq)**	D:\Projects\2023\Methods\General Positive organic analysis method-3 .m
**Position**	P1-A2	**Acq. Time (Local)**	6/6/2025 11:41:31 AM (UTC+08:00)
**MS Type**	QTOF	**Ion Polarity**	Positive
**Instrument**	G6545B	**Version (Acq SW)**	6200 series TOF/6500 series Q-TOF (11.0.221.1)
**Operator**	SYSTEM (SYSTEM)		

Sample Spectra

+ Scan (rt: 28.554 min)

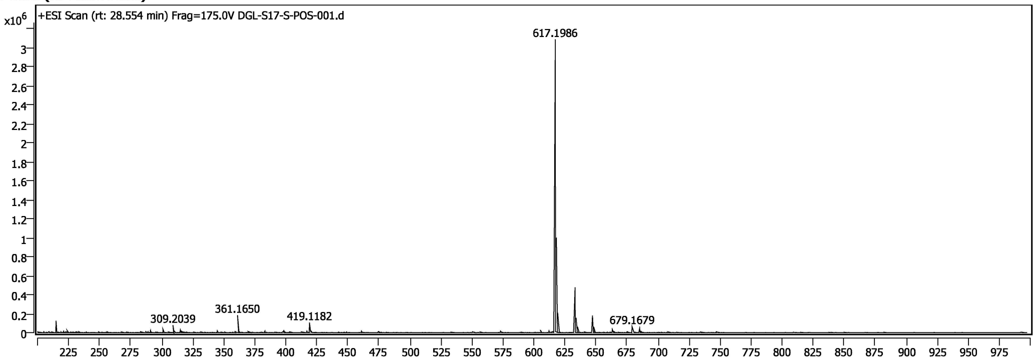

Spectrum Identification Table

Formula	m/z	Mass	Species	Score	Diff (ppm)	Diff (mDa)
C30 H33 F3 O9	617.1986	594.2092	(M+Na)+	96.11	2.53	1.5
C24 H35 F5 O11	617.1986	594.2092	(M+Na)+	94.17	-1.29	-0.8
C30 H36 F2 O8 S	617.1986	594.2094	(M+Na)+	93.20	-0.78	-0.5

MassHunter Qual 10.0
(End of Report)

MassHunter Qualitative Analysis   Page 1 of 1   Generated at 3:57 PM on 9/1/2025

Figure S67. HRESIMS spectrum of **7Rb**.

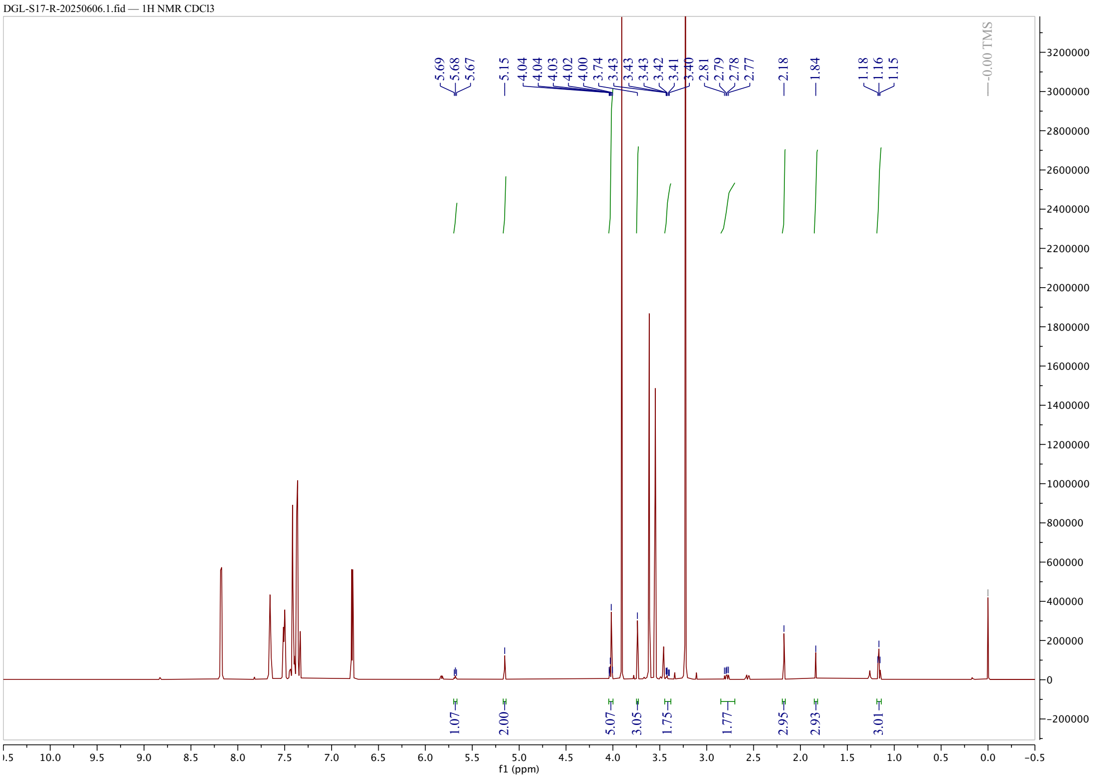

Figure S68. ^{1}H NMR spectrum of **7Ra** ($CDCl_3$, 600 MHz).

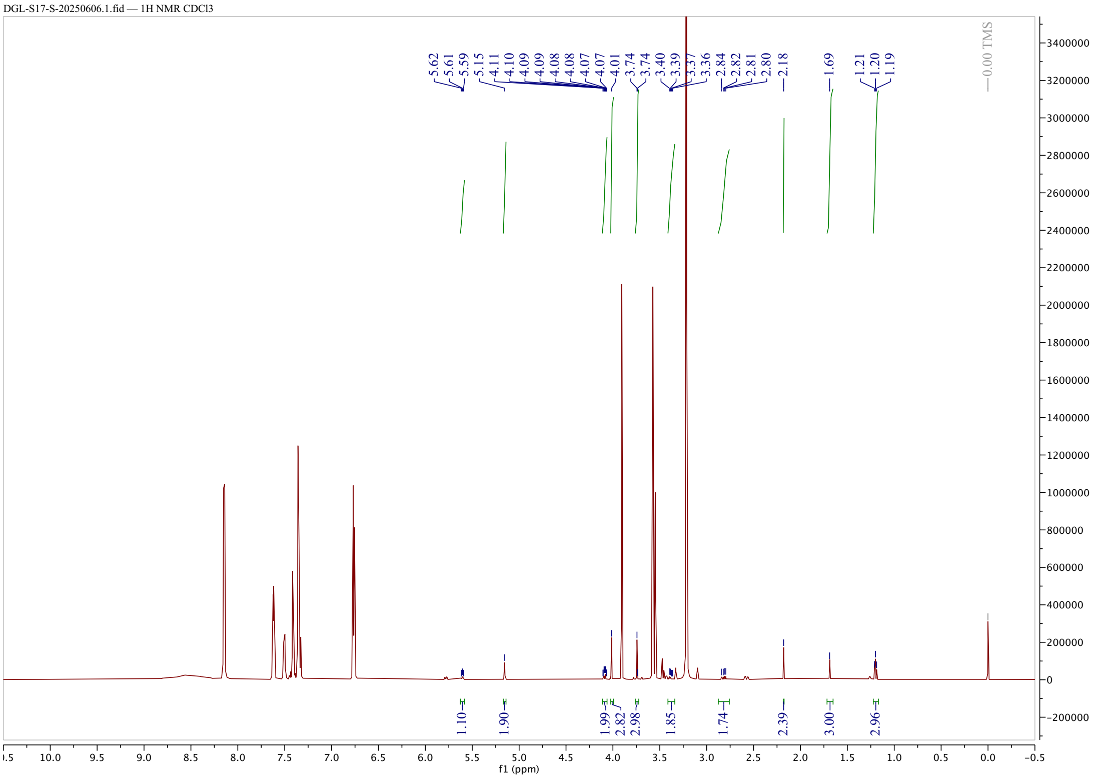

Figure S69. ^{1}H NMR spectrum of **7Rb** ($CDCl_3$, 600 MHz).

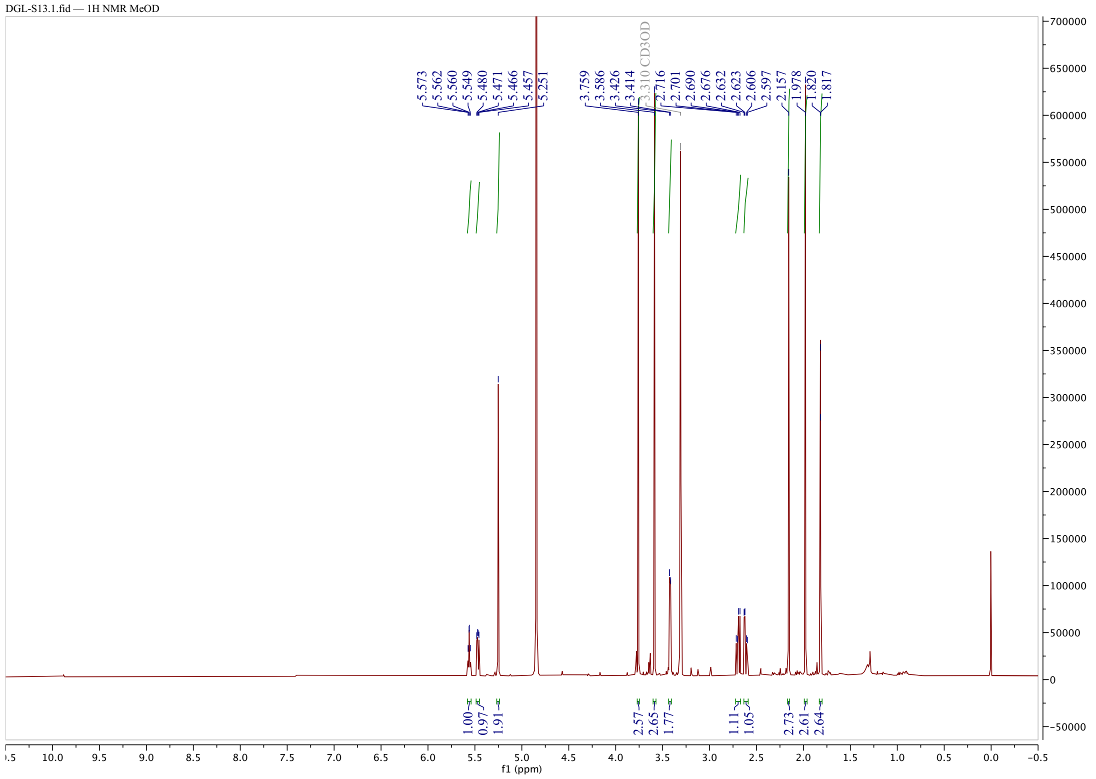

Figure S70. ^{1}H NMR spectrum of **8** (CD_3OD, 600 MHz).

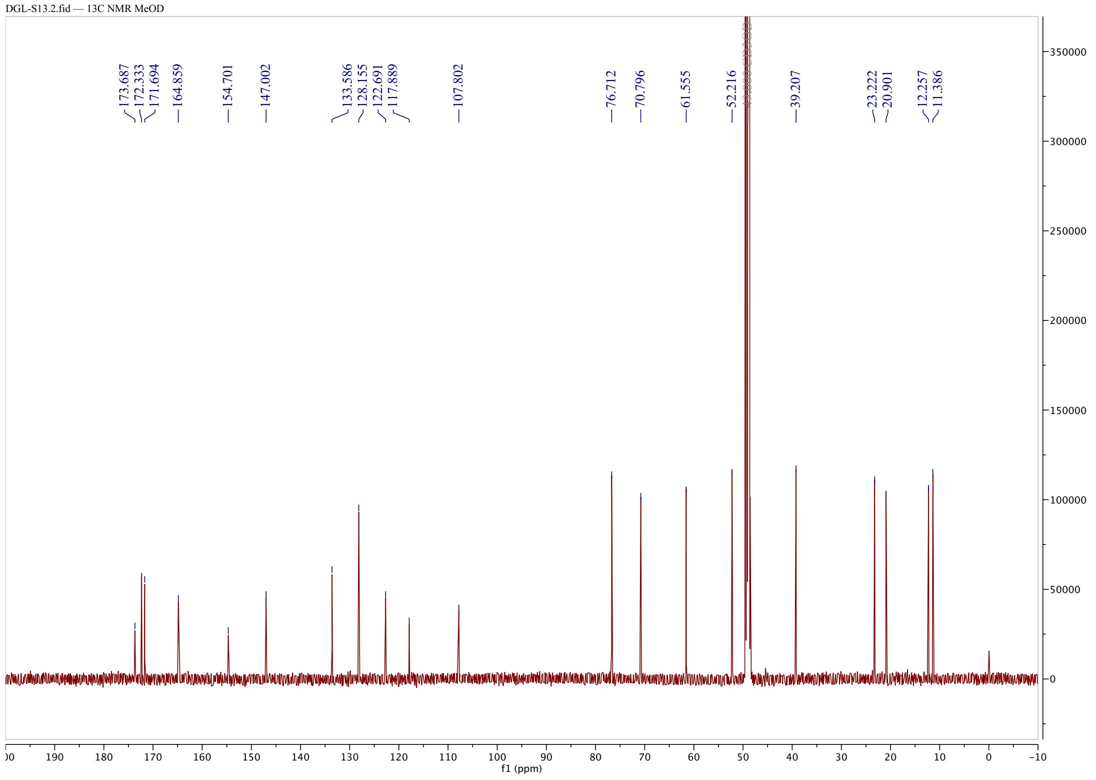

Figure S71. ^{13}C NMR spectrum of **8** (CD_3OD, 151 MHz).

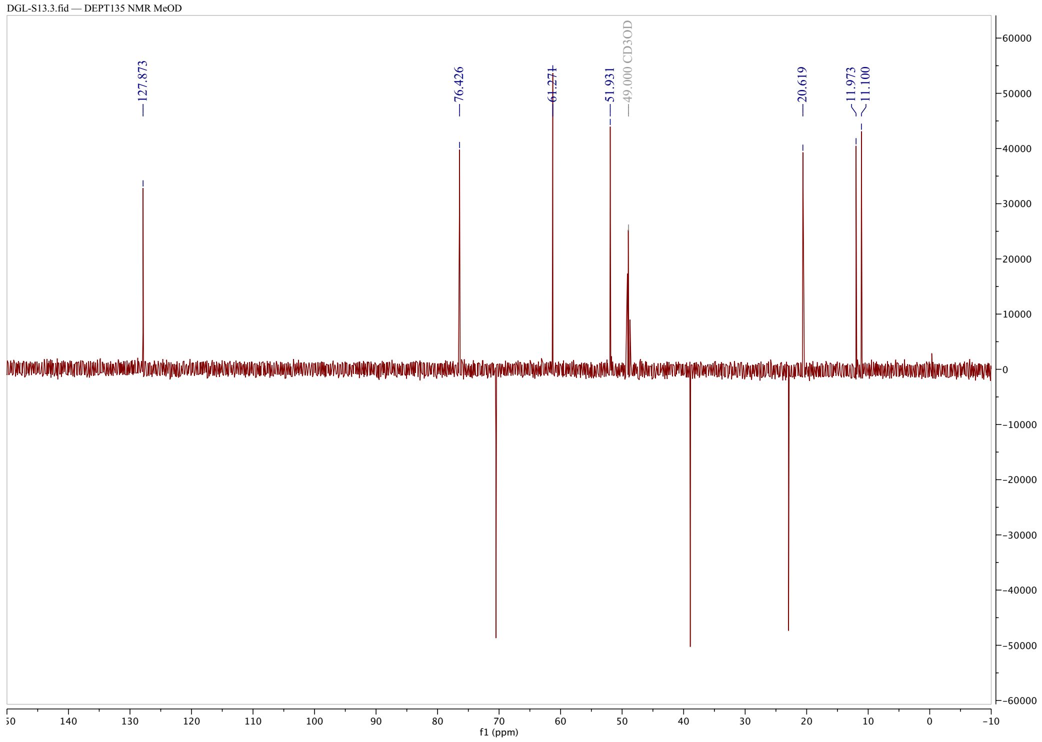

Figure S72. DEPT spectrum of **8** (CD_3OD).

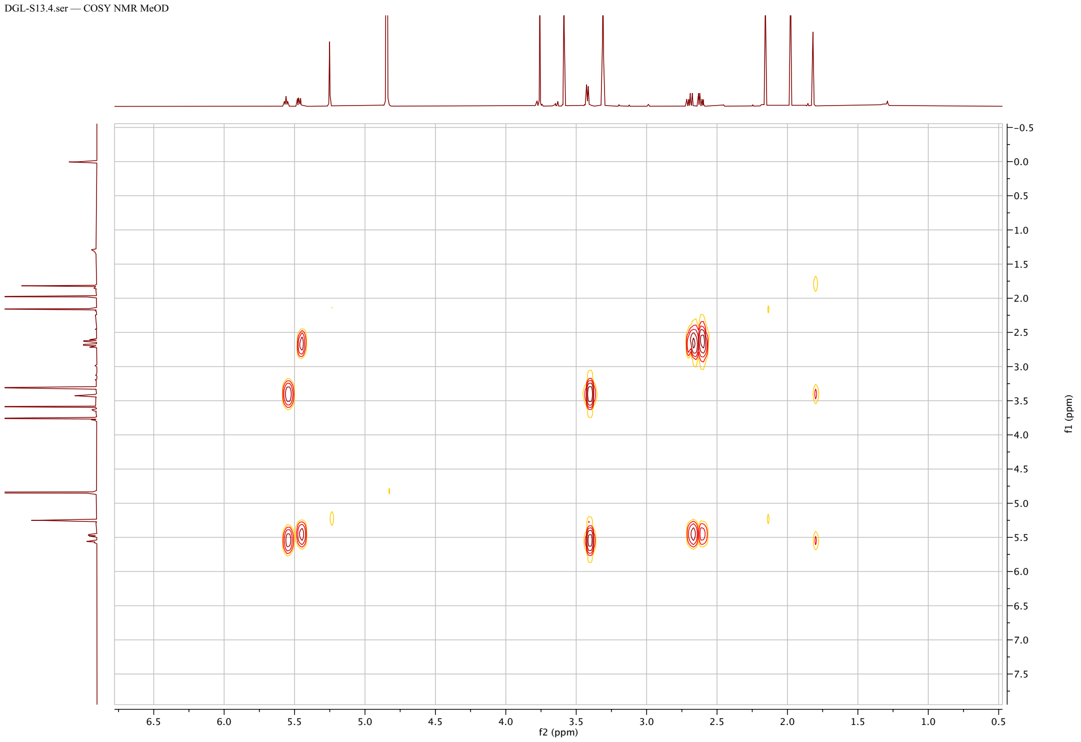

Figure S73. COSY spectrum of **8** (CD_3OD).

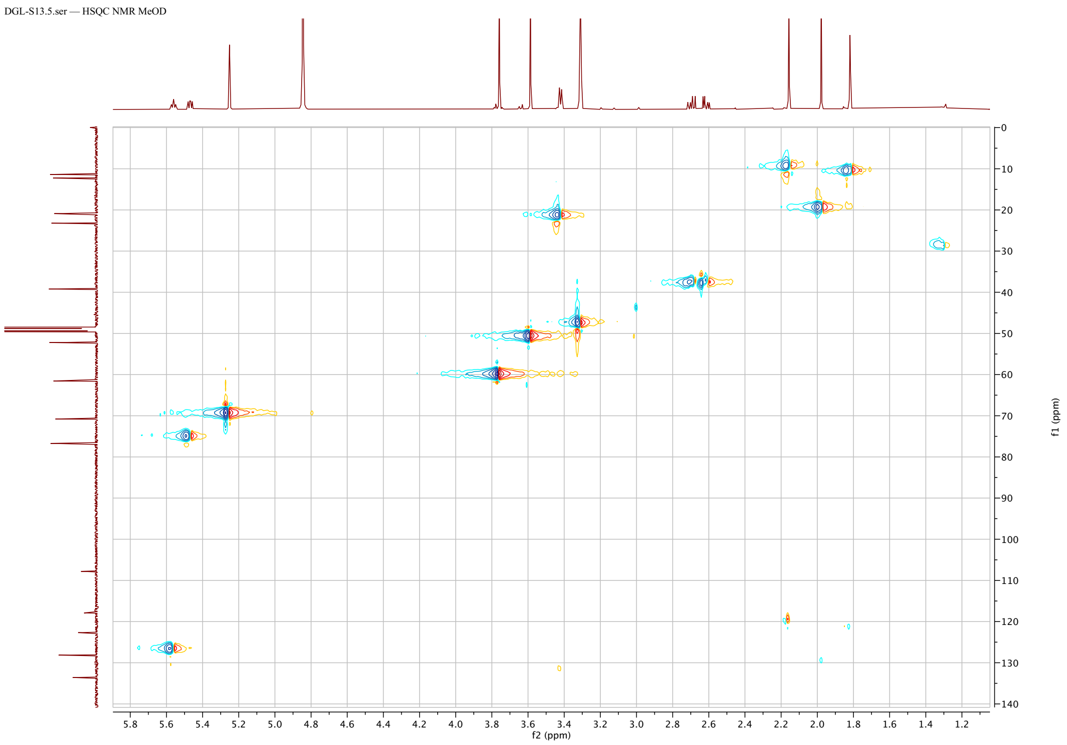

Figure S74. HSQC spectrum of **8** (CD_3OD).

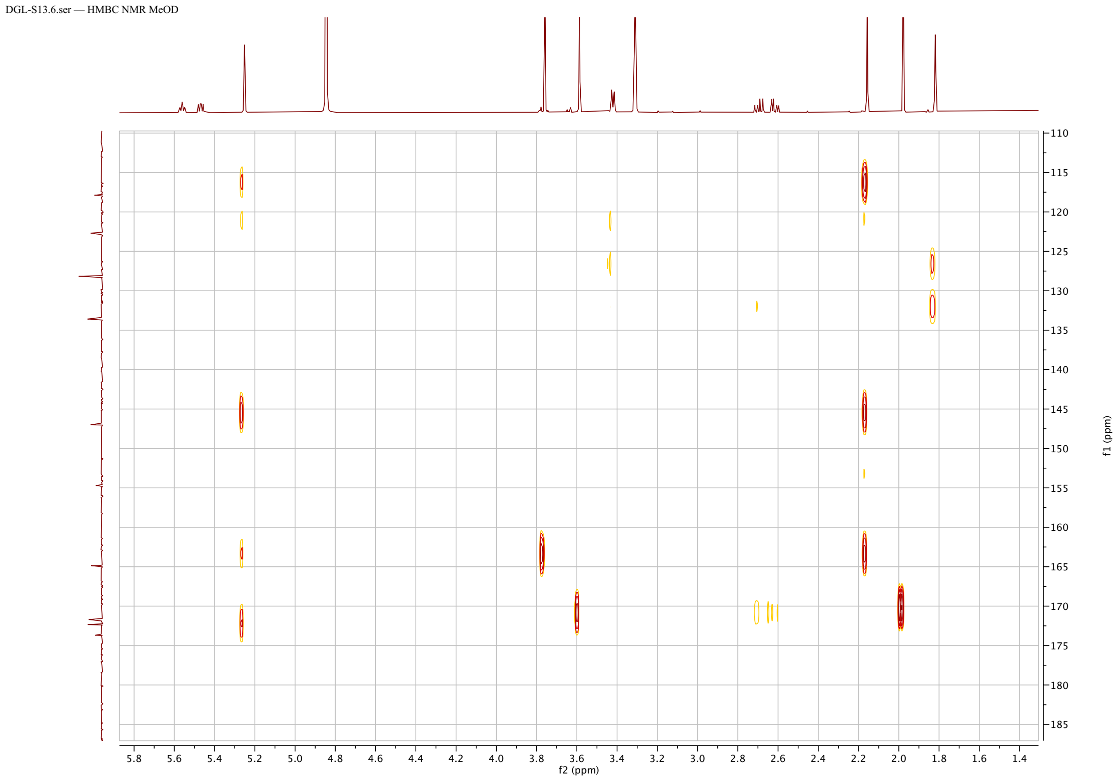

Figure S75. HMBC spectrum of **8** (CD_3OD).

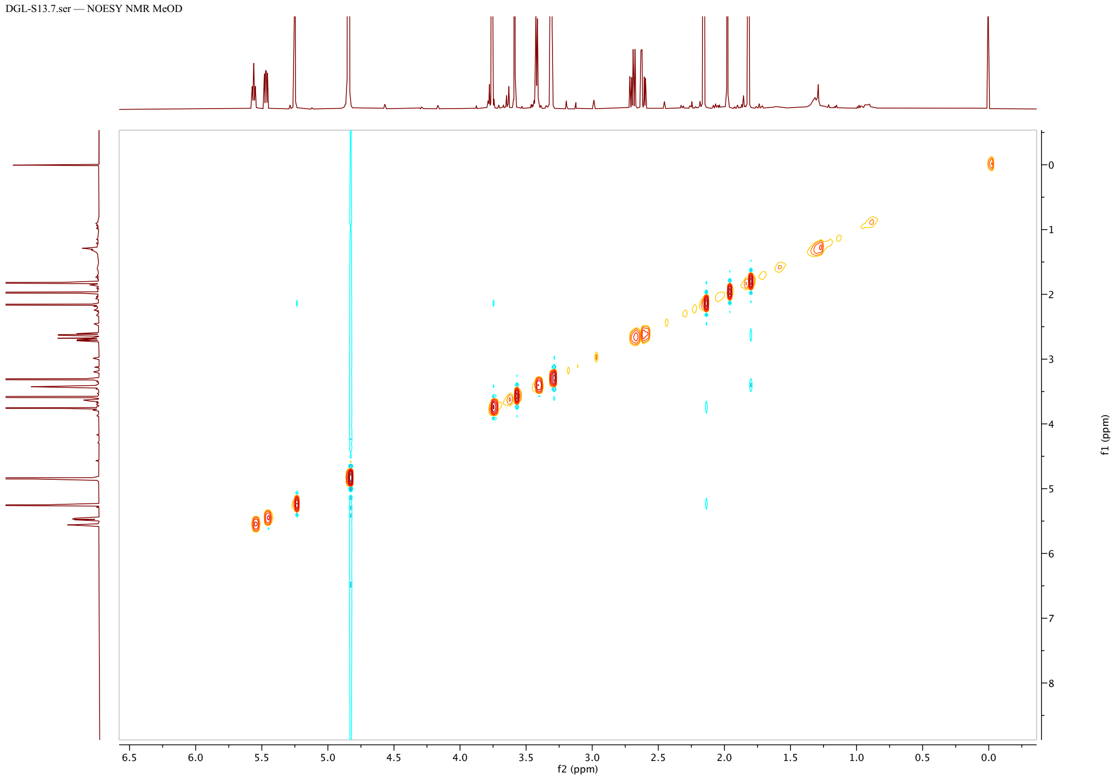

Figure S76. NOESY spectrum of **8** (CD_3OD).

Analysis Report

Agilent | Trusted Answers

Sample Information

**Name**	DGL-S13-POS-02	**Data File Path**	D:\Projects\2023\Data\RCDB\DGL\20241121\DGL-S13-POS-02.d
**Inj. Vol. (ul)**	10	**Method Path (Acq)**	D:\Projects\2023\Methods\General positive organic analysis method-1 .m
**Position**	P1-A1	**Acq. Time (Local)**	11/22/2024 1:24:28 PM (UTC+08:00)
**MS Type**	QTOF	**Ion Polarity**	Positive
**Instrument**	G6545B	**Version (Acq SW)**	6200 series TOF/6500 series Q-TOF (11.0.221.1)
**Operator**	SYSTEM (SYSTEM)		

Sample Spectra

+ Scan (rt: 13.092 min)

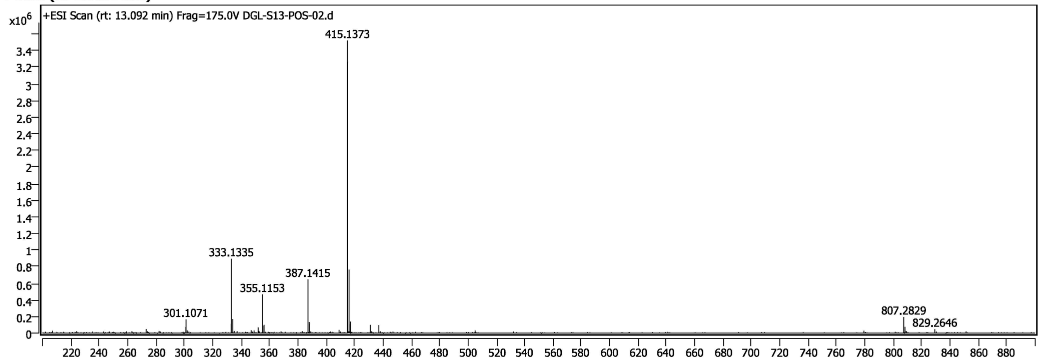

Spectrum Peaks

m/z	z	m/z (Calc)	Ion Species	Formula	Diff (ppm)	Diff (mDa)
415.1373	1	415.1363	(M+Na)+	C20 H24 O8	2.41	1.0
416.1399	1	416.1397	(M+Na)+	C20 H24 O8	0.49	0.2
417.0861	1	417.0866	(M+Na)+	C19 H16 N5 O3 S	-1.18	-0.5
417.1422	1	417.1421	(M+Na)+	C20 H24 O8	0.24	0.1

Spectrum Identification Table

Formula	m/z	Mass	Species	Score	Diff (ppm)	Diff (mDa)
C20 H24 O8	415.1373	392.1480	(M+Na)+	97.46	2.13	0.8
C21 H20 N4 O4	415.1373	392.1480	(M+Na)+	97.34	-1.10	-0.4
C19 H18 N7 O3	415.1373	392.1481	(M+Na)+	96.54	2.50	1.0
C19 H16 N5 O3 S	417.0861	394.0969	(M+Na)+	95.39	-1.19	-0.5
C17 H14 N8 O2 S	417.0861	394.0970	(M+Na)+	92.53	2.46	1.0

MassHunter Qual 10.0
(End of Report)

MassHunter Qualitative Analysis | Page 1 of 1 | Generated at 4:21 PM on 12/2/2024

Figure S77. HRESIMS spectrum of **8**.

RCDB

#	样品ID	用户名	日期和时间	型号#	起始波长	结束波长
12	S13 20ug/ml	DELL	2025/2/20 15:43:40	Evo350	200.00	400.00

峰：：

nm	Abs
215.612	1.891
249.731	0.399
304.079	0.214

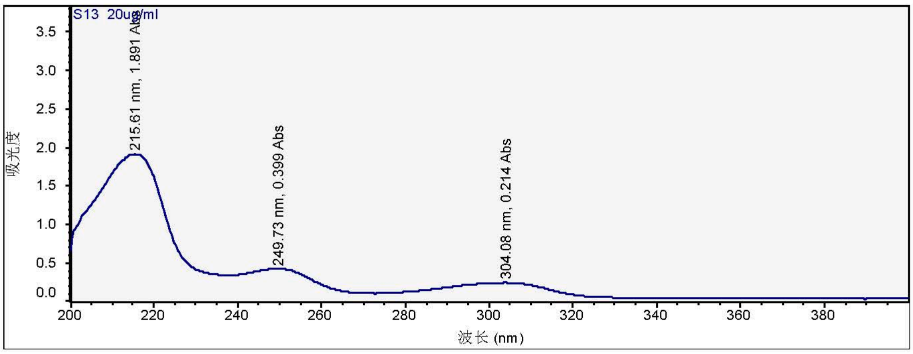

INSIGHT Software 第 1 页，共 1 页

Figure S78. UV spectrum of **8**.

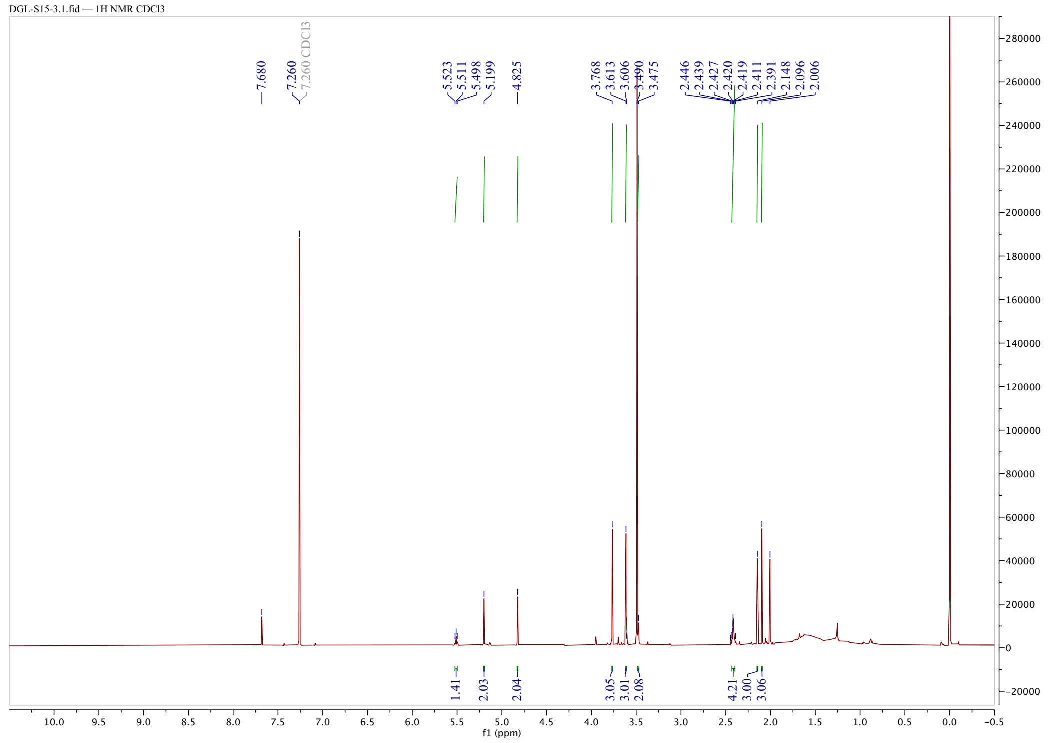

Figure S79. ^{1}H NMR spectrum of **9** ($CDCl_3$, 600 MHz).

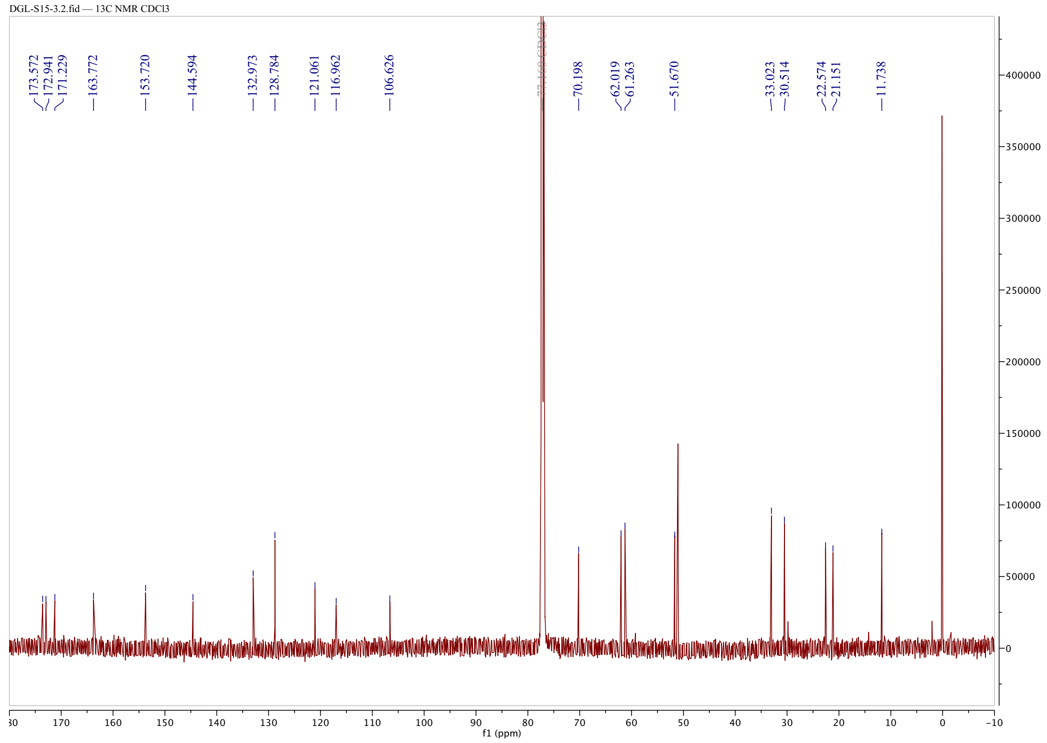

Figure S80. ^{13}C NMR spectrum of **9** ($CDCl_3$, 151 MHz).

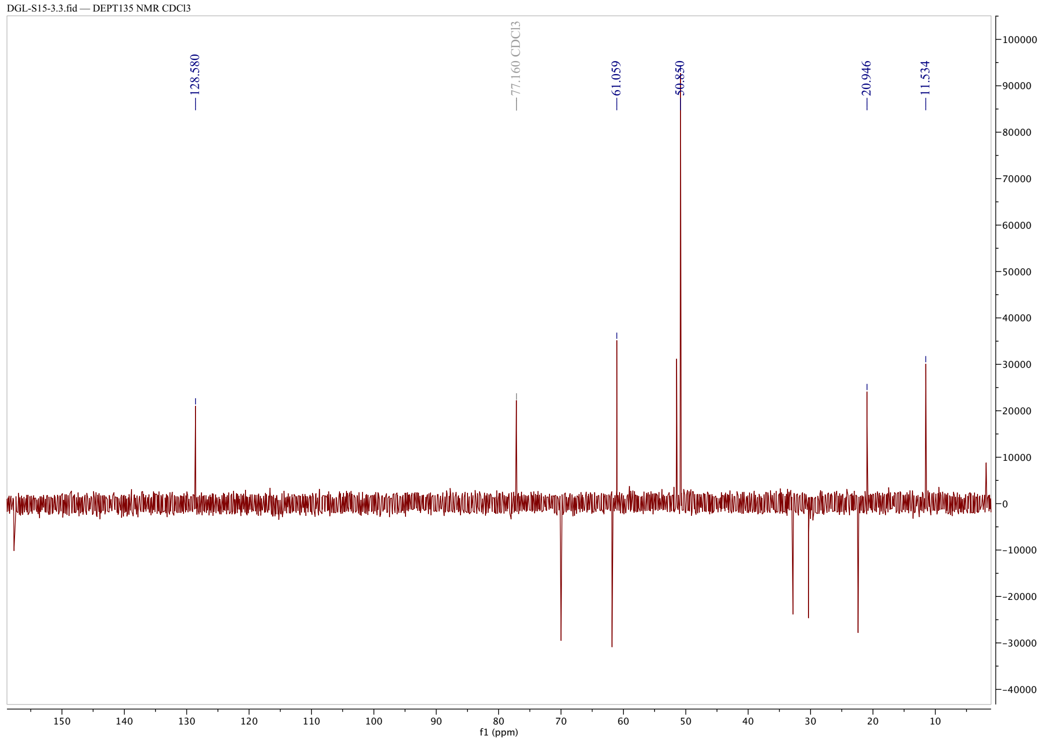

Figure S81. DEPT spectrum of **9** ($CDCl_3$).

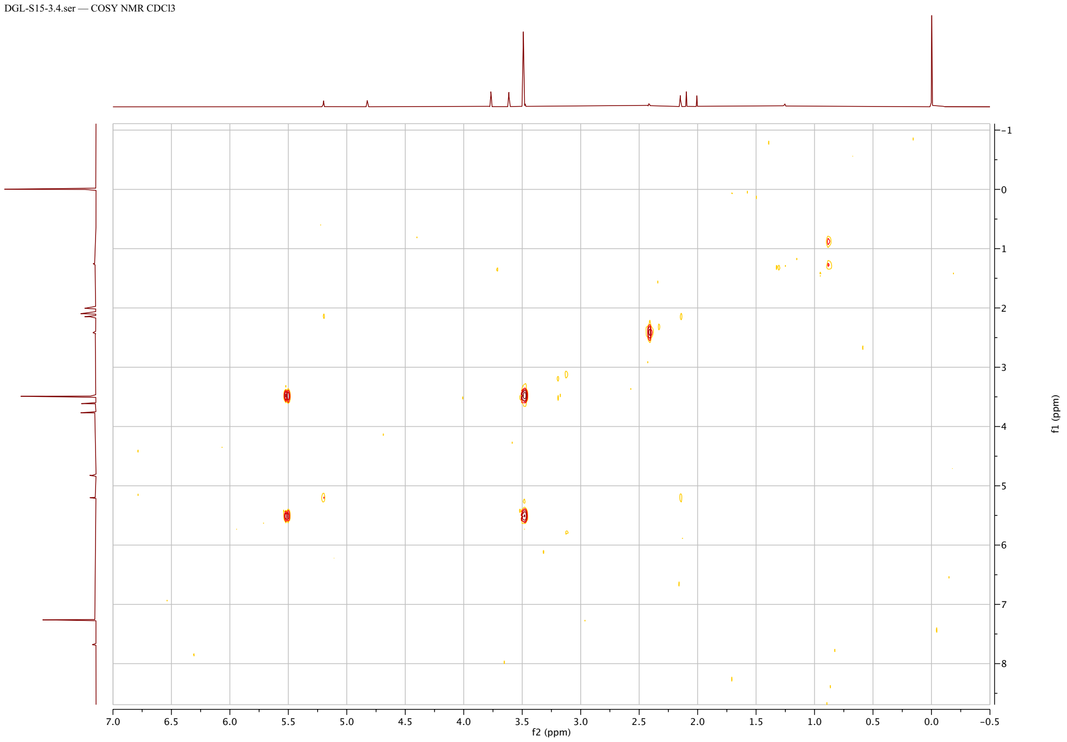

Figure S82. COSY spectrum of **9** ($CDCl_3$).

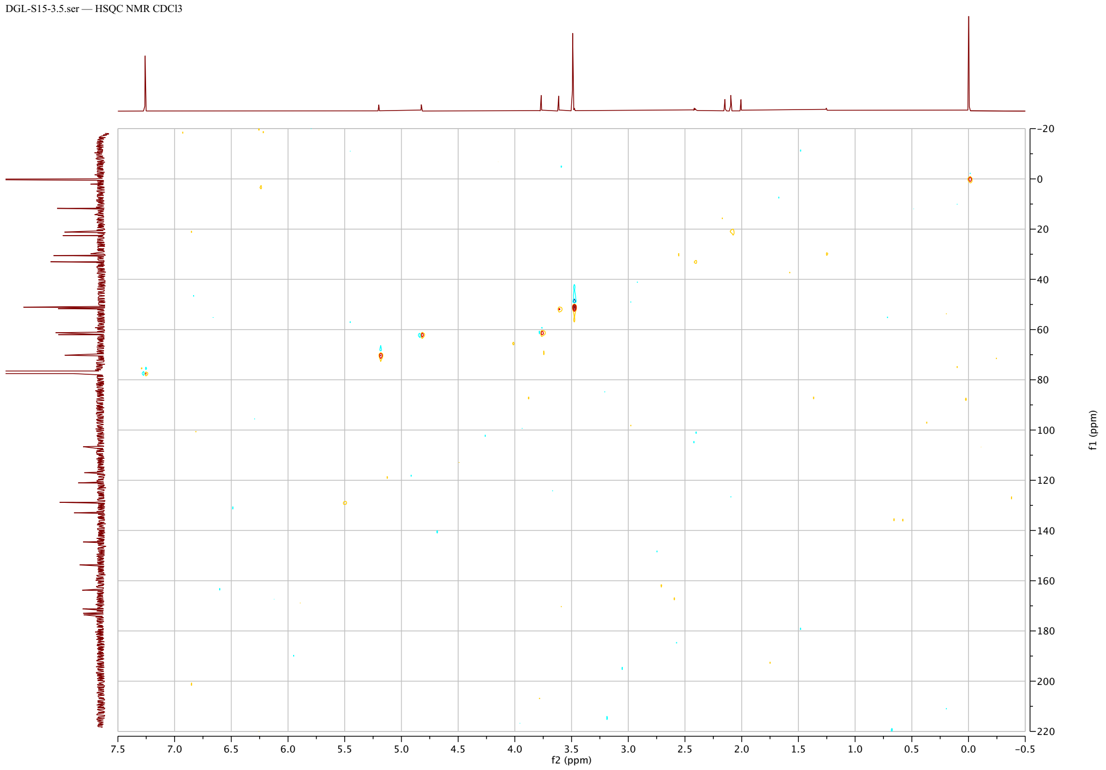

Figure S83. HSQC spectrum of **9** ($CDCl_3$).

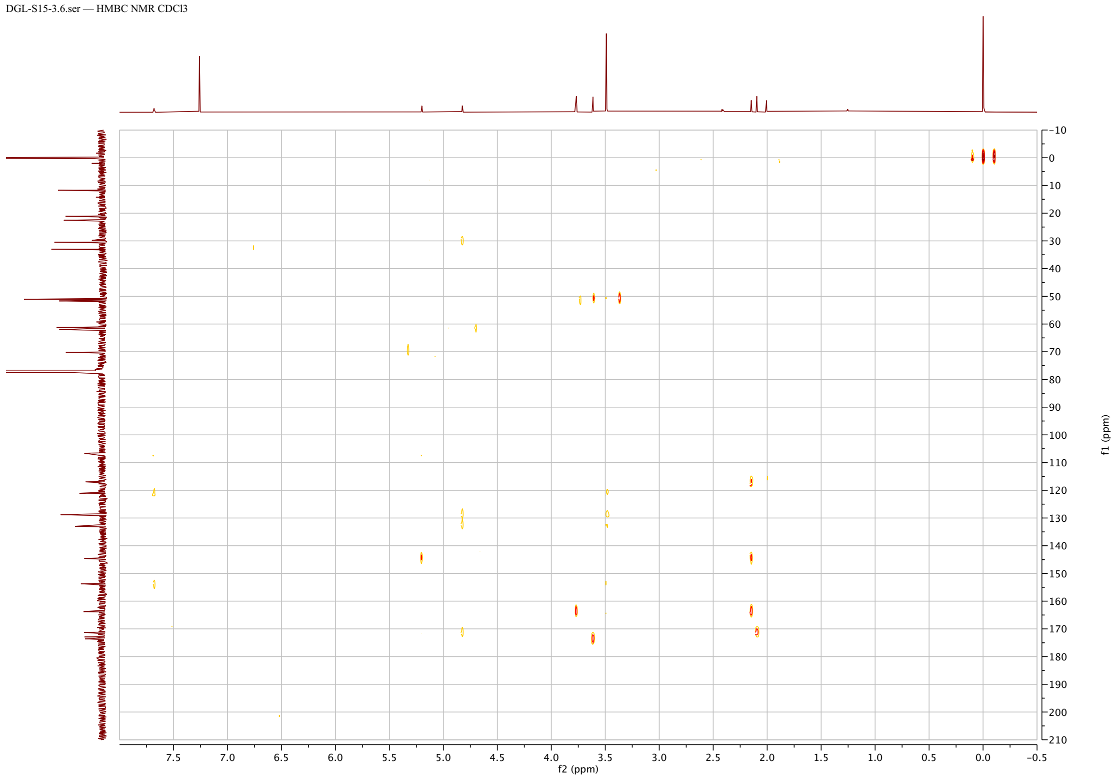

Figure S84. HMBC spectrum of **9** ($CDCl_3$).

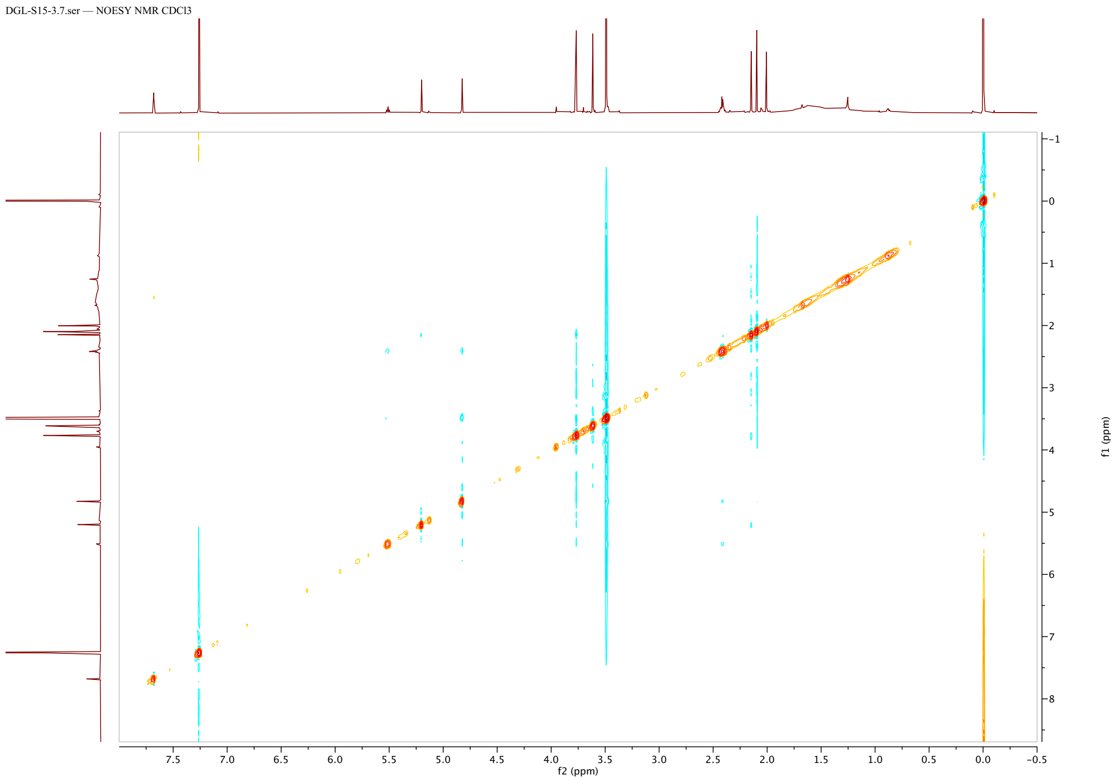

Figure S85. NOESY spectrum of **9** ($CDCl_3$).

Analysis Report

Sample Information

**Name**	DGL-S15-3-POS-02	**Data File Path**	D:\Projects\2023\Data\RCDB\DGL\20250416\DGL-S15-3-POS-02.d
**Inj. Vol. (ul)**	0.5	**Method Path (Acq)**	D:\Projects\2023\Methods\General Positive organic analysis method-3 .m
**Position**	P1-A1	**Acq. Time (Local)**	4/17/2025 12:27:01 PM (UTC+08:00)
**MS Type**	QTOF	**Ion Polarity**	Positive
**Instrument**	G6545B	**Version (Acq SW)**	6200 series TOF/6500 series Q-TOF (11.0.221.1)
**Operator**	SYSTEM (SYSTEM)		

Sample Spectra

+ Scan (rt: 12.238 min)

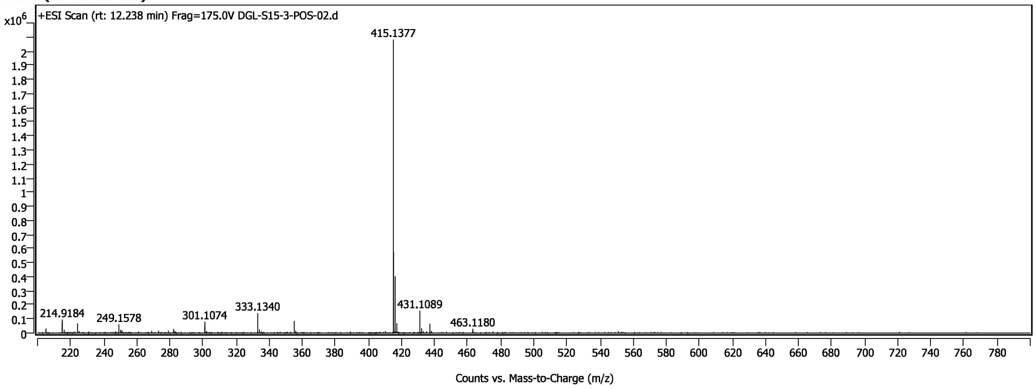

Spectrum Identification Table

Formula	m/z	Mass	Species	Score	Diff (ppm)	Diff (mDa)
C20 H24 O8	415.1377	392.1485	(M+Na)+	93.78	3.54	1.4
C14 H20 N10 O2 S	415.1377	392.1489	(M+Na)+	93.00	-0.51	-0.2
C15 H26 N3 O7 S	415.1377	392.1488	(M+Na)+	92.45	-0.93	-0.4
C19 H18 N7 O3	415.1377	392.1486	(M+Na)+	90.62	3.89	1.5

MassHunter Qual 10.0
(End of Report)

Figure S86. HRESIMS spectrum of **9**.

RCDB

样品ID	日期和时间	积分时间 (s)	扫描速度	数据间隔	起始波长	结束波长	带宽	型号#
S15-3-16	2025/4/15 14:35:06	0.05	1200.00	1.00	200.00	400.00	1.0 nm	Evo350

峰：:

nm	Abs
216.385	1.440
249.598	0.333
304.660	0.163

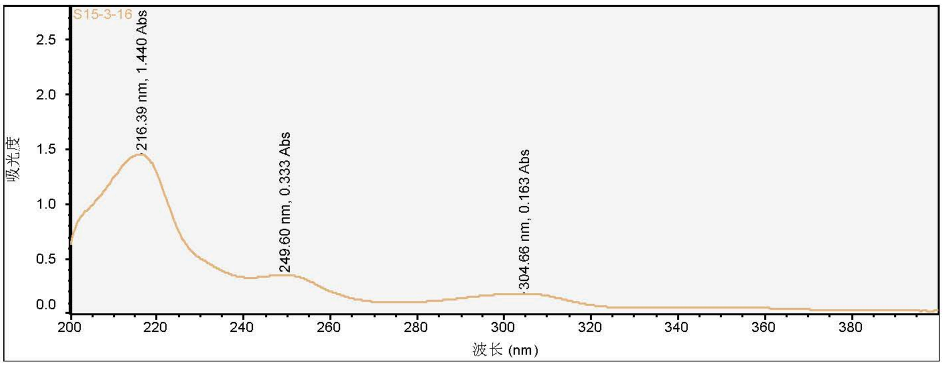

INSIGHT Software 第 1 页，共 1 页

Figure S87. UV spectrum of **9**.

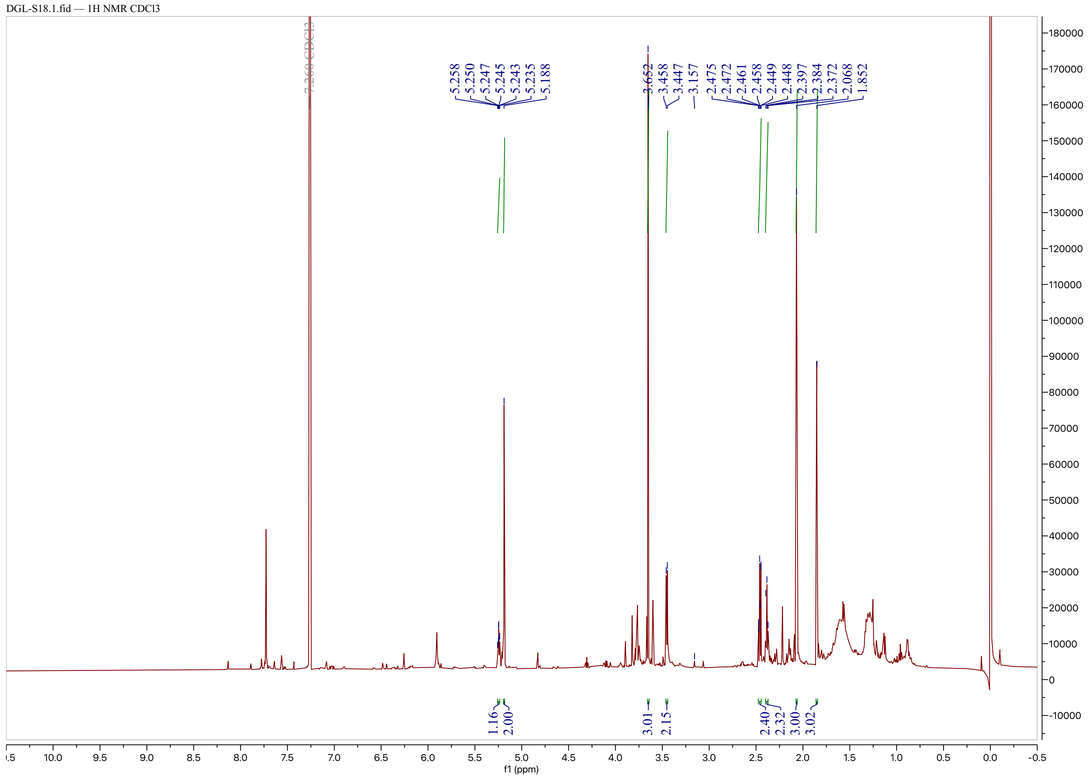

Figure S88. ^{1}H NMR spectrum of **10** ($CDCl_3$, 600 MHz).

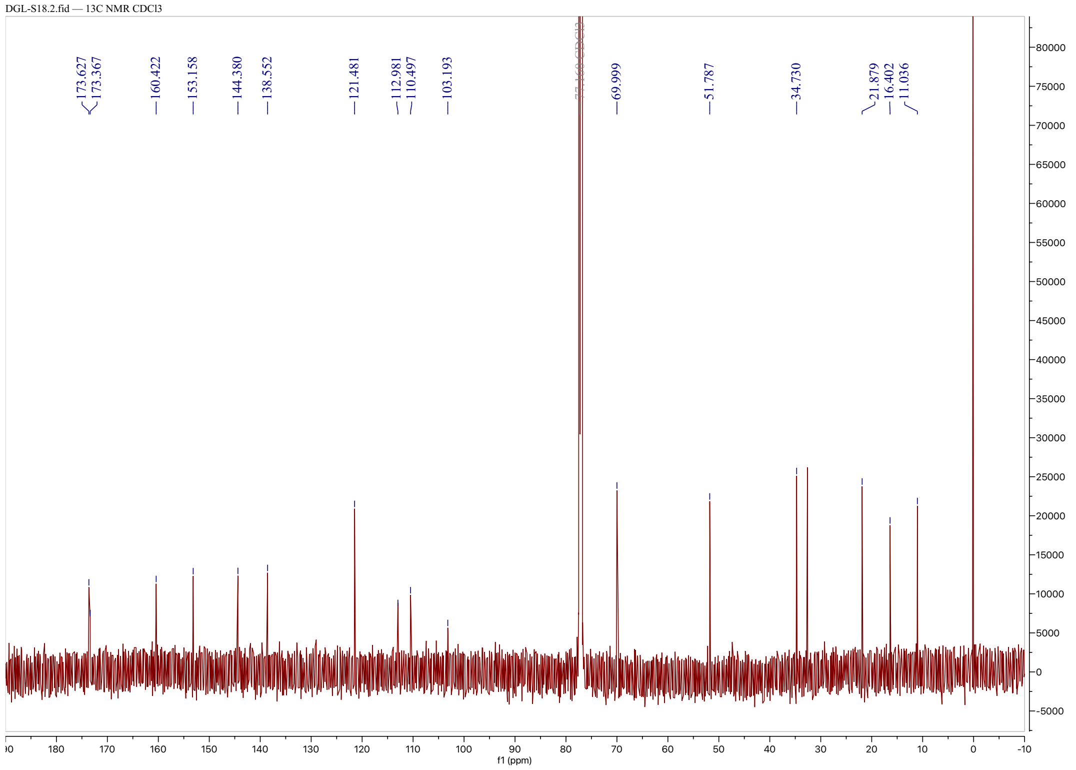

Figure S89. ^{13}C NMR spectrum of **10** ($CDCl_3$, 151 MHz).

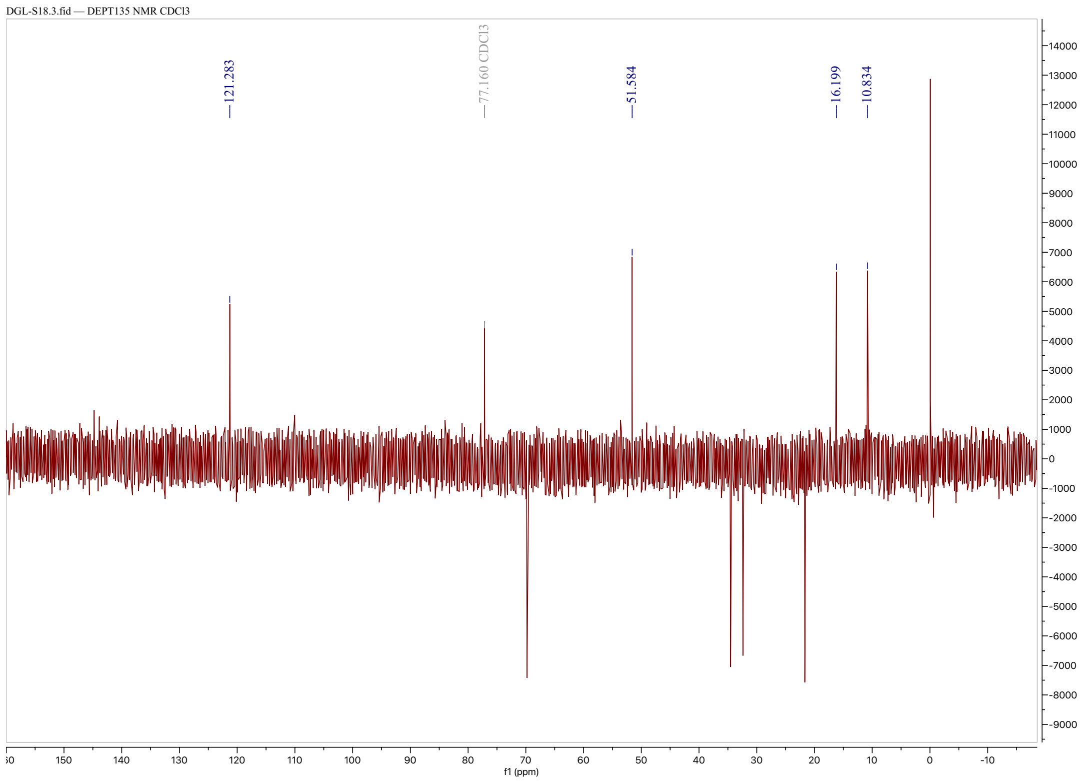

Figure S90. DEPT spectrum of **10** ($CDCl_3$).

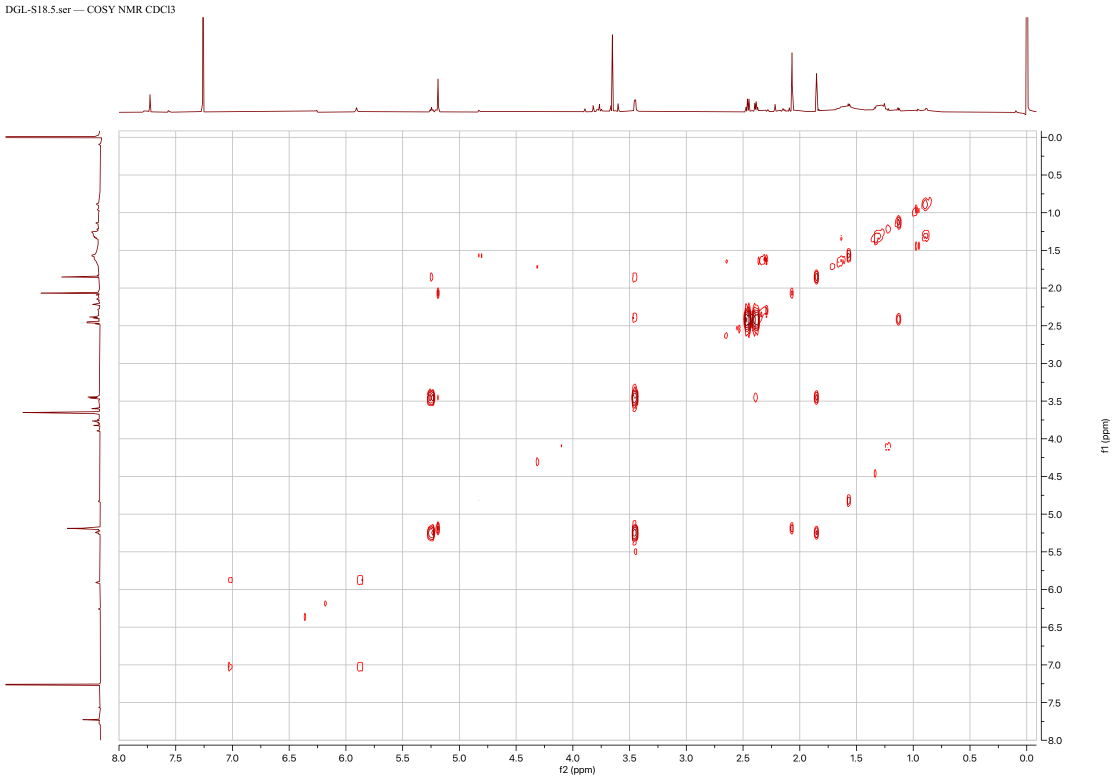

Figure S91. COSY spectrum of **10** ($CDCl_3$).

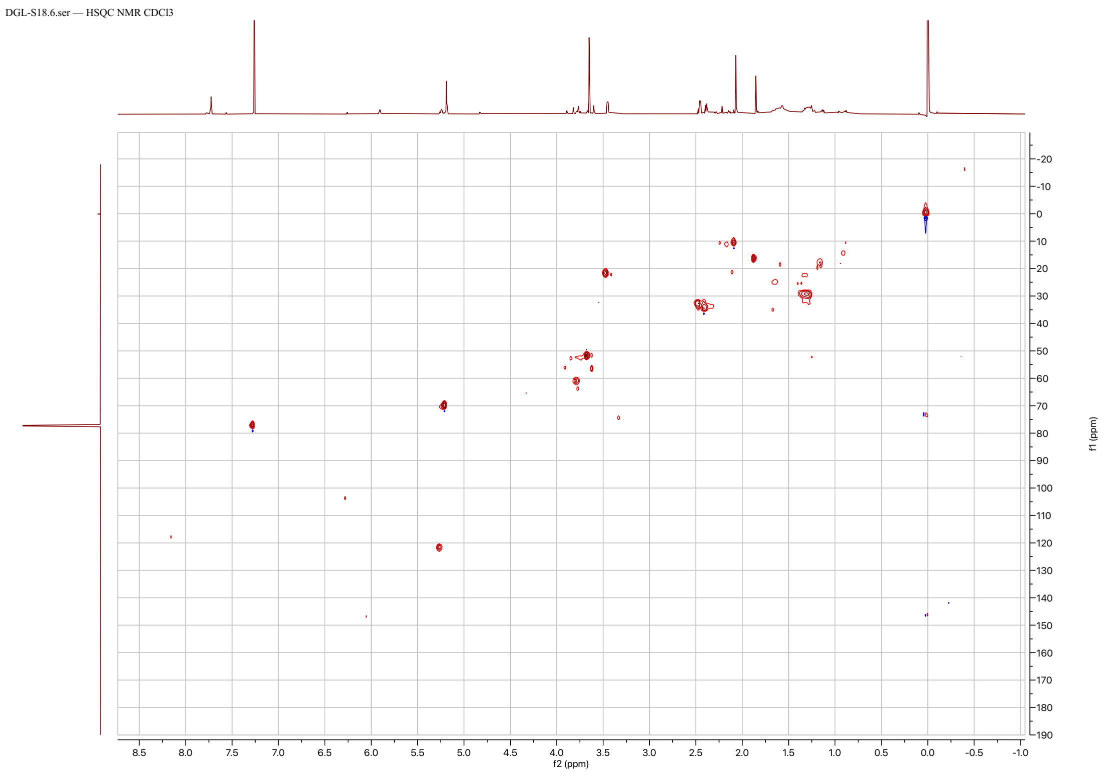

Figure S92. HSQC spectrum of **10** ($CDCl_3$).

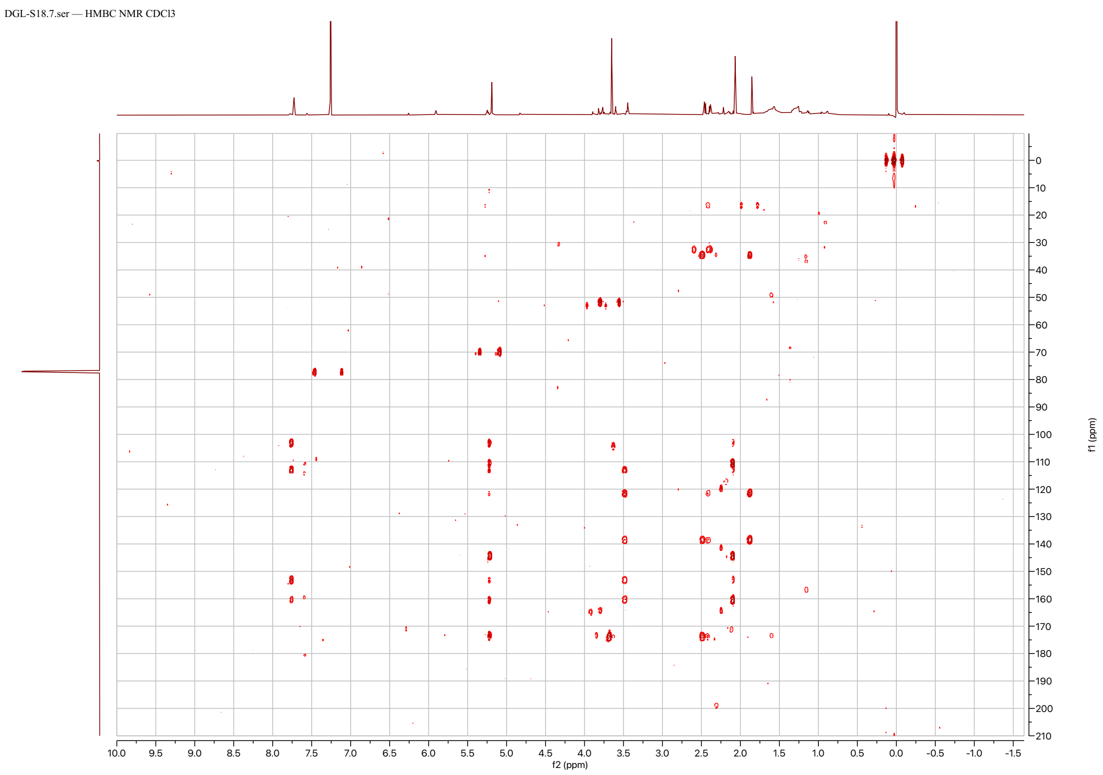

Figure S93. HMBC spectrum of **10** ($CDCl_3$).

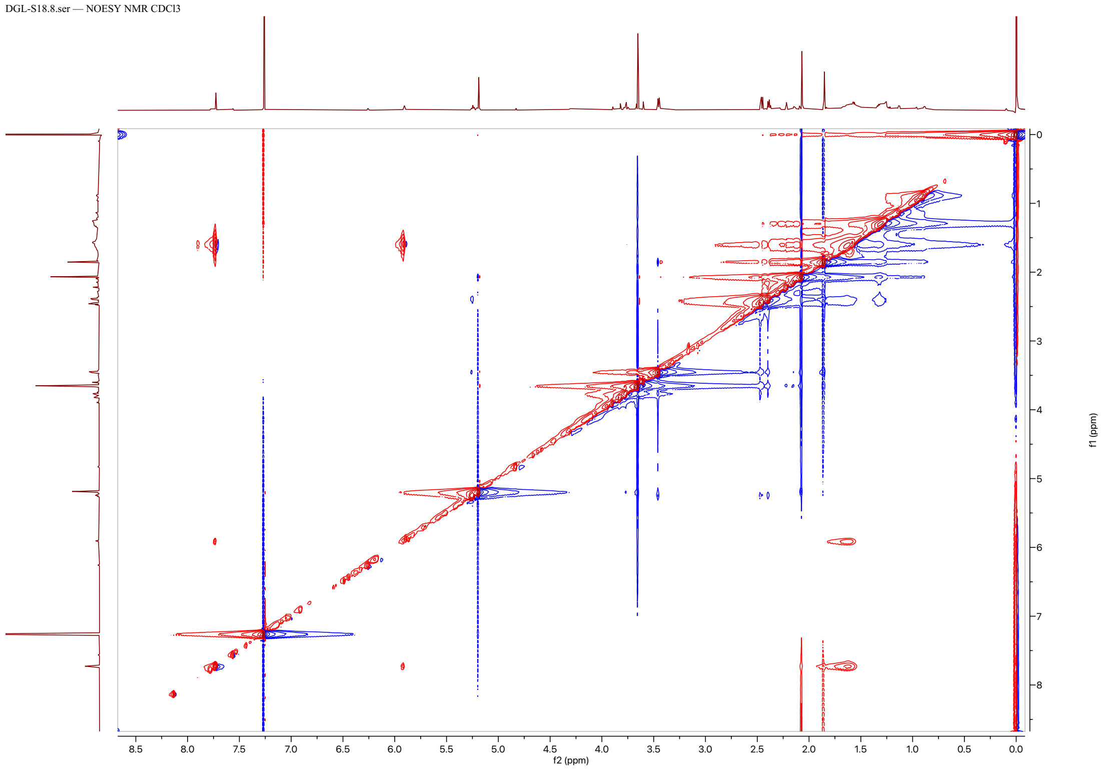

Figure S94. NOESY spectrum of **10** ($CDCl_3$).

Analysis Report

Agilent | Trusted Answers

Sample Information

**Name**	DGL-S18-POS-02	**Data File Path**	D:\Projects\2023\Data\RCDB\DGL\20241129\DGL-S18-NEG-01.d
**Inj. Vol. (ul)**	1	**Method Path (Acq)**	D:\Projects\2023\Methods\General negative organic analysis method-1 .m
**Position**	P1-A2	**Acq. Time (Local)**	11/29/2024 2:13:51 PM (UTC+08:00)
**MS Type**	QTOF	**Ion Polarity**	Negative
**Instrument**	G6545B	**Version (Acq SW)**	6200 series TOF/6500 series Q-TOF (11.0.221.1)
**Operator**	SYSTEM (SYSTEM)		

Sample Spectra

- Scan (rt: 9.774 min)

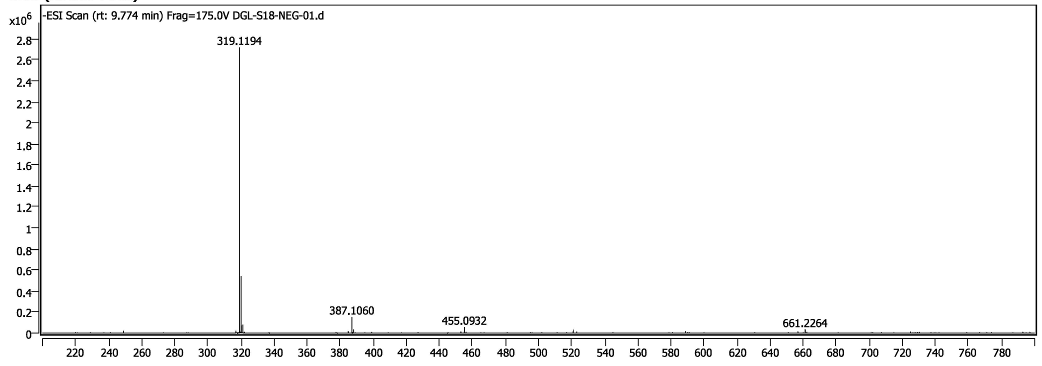

Spectrum Peaks

m/z	z	m/z (Calc)	Ion Species	Formula	Diff (ppm)	Diff (mDa)
319.1194	1	319.1187	(M-H)-	C17 H20 O6	2.27	0.7
320.1226	1	320.1221	(M-H)-	C17 H20 O6	1.60	0.5
321.1247	1	321.1244	(M-H)-	C17 H20 O6	0.78	0.3

Spectrum Identification Table

Formula	m/z	Mass	Species	Score	Diff (ppm)	Diff (mDa)
C17 H20 O6	319.1194	320.1267	(M-H)-	98.33	2.12	0.7
C18 H16 N4 O2	319.1194	320.1267	(M-H)-	97.98	-1.82	-0.6
C16 H14 N7 O	319.1194	320.1268	(M-H)-	96.85	2.61	0.8
C11 H16 N10 S	319.1194	320.1271	(M-H)-	87.40	-2.71	-0.9
C20 H18 N O3	319.1194	320.1267	(M-H)-	86.66	-6.22	-2.0

MassHunter Qual 10.0
(End of Report)

Figure S95. HRESIMS spectrum of **10**.

RCDB

样品ID	日期和时间	积分时间 (s)	扫描速度	数据间隔	起始波长	结束波长	带宽	型号#
样品S18-30	2025/2/25 11:27:44	0.05	1200.00	1.00	200.00	400.00	0.5 nm	Evo350

峰：:

nm	Abs
218.110	1.852
260.023	0.644

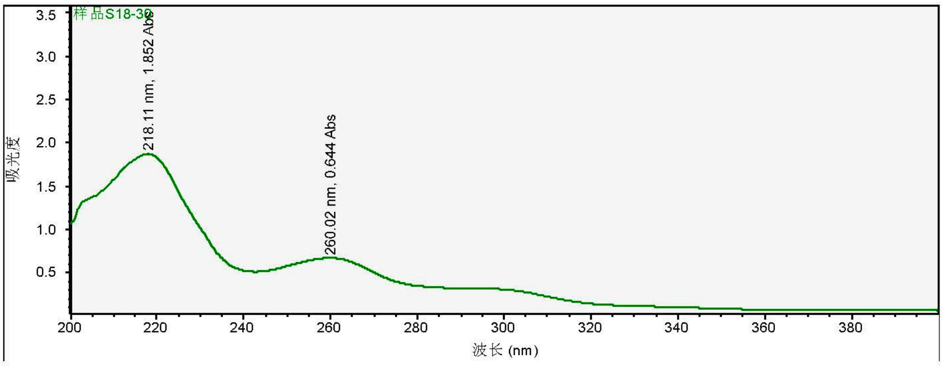

Figure S96. UV spectrum of **10**.

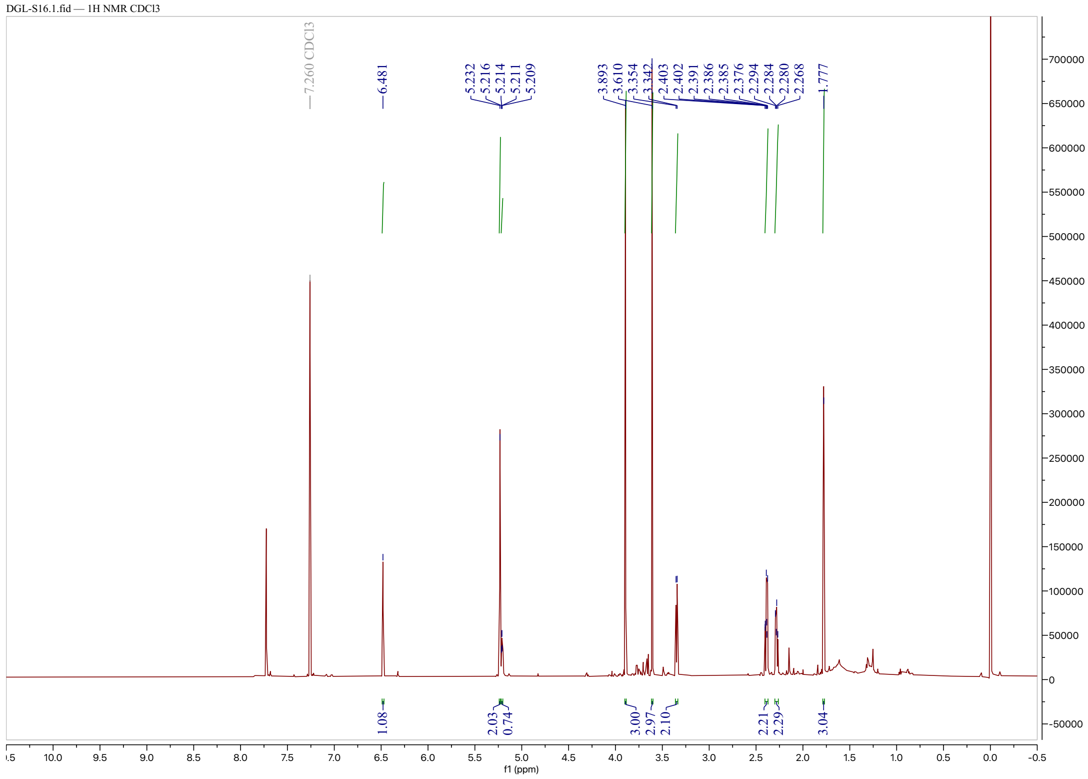

Figure S97. ^{1}H NMR spectrum of **11** ($CDCl_3$, 600 MHz).

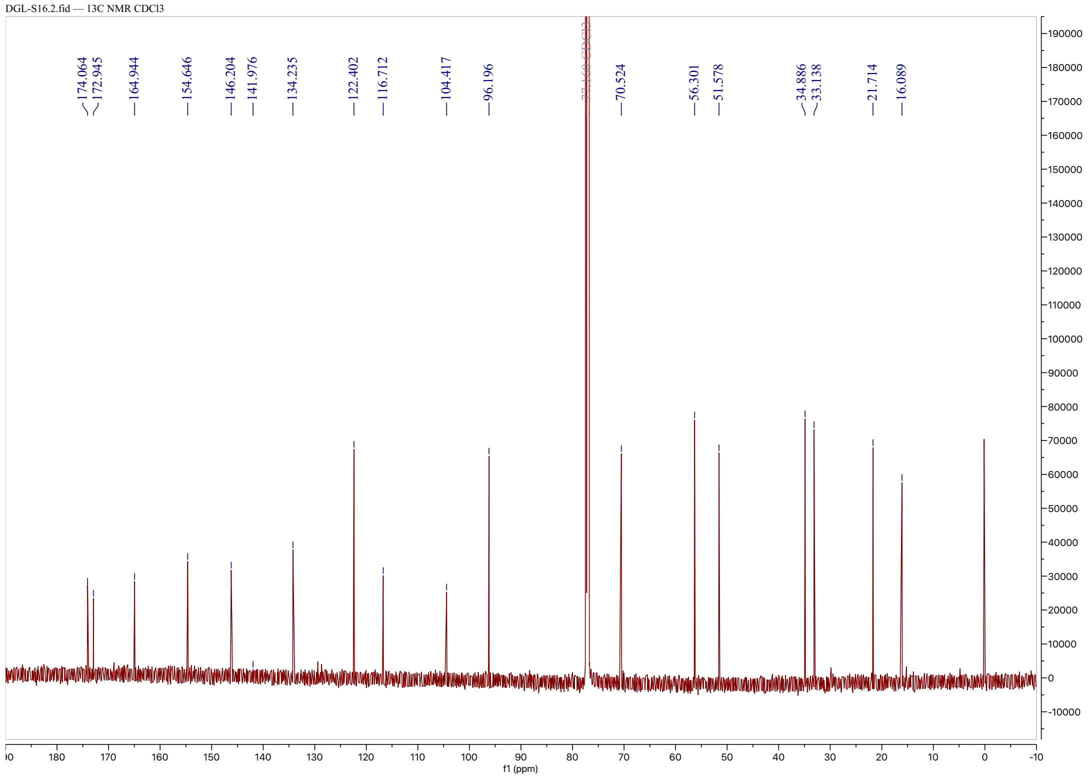

Figure S98. ^{13}C NMR spectrum of **11** ($CDCl_3$, 151 MHz).

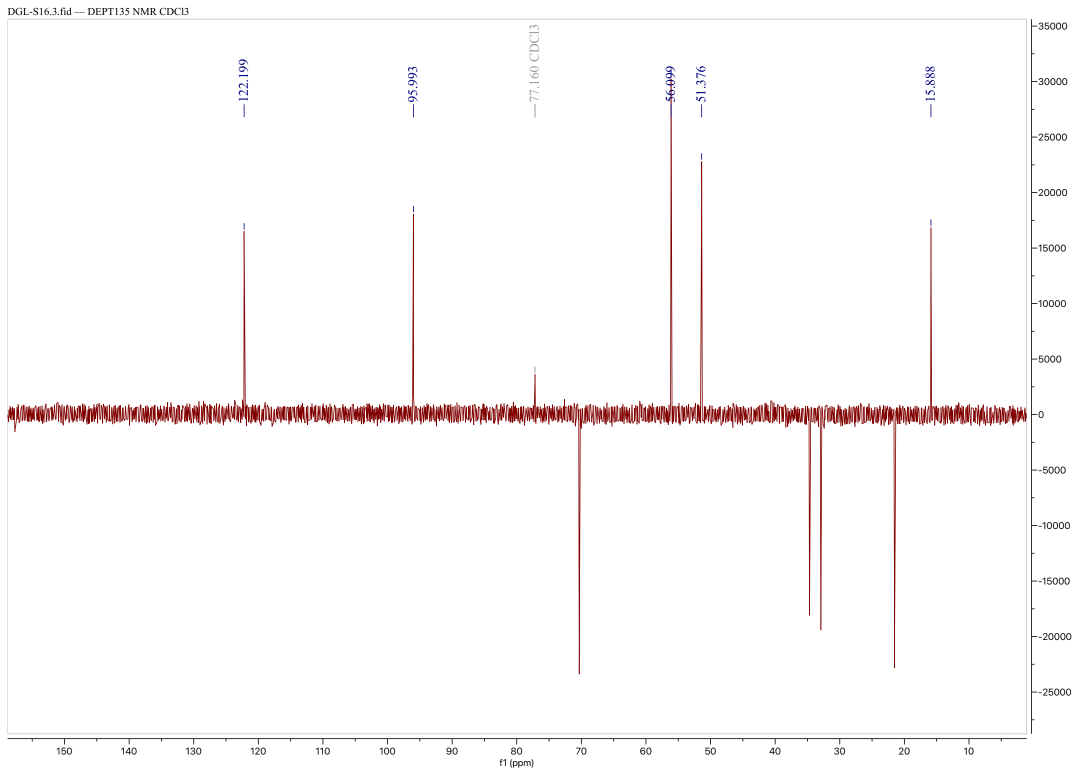

Figure S99. DEPT spectrum of **11** ($CDCl_3$).

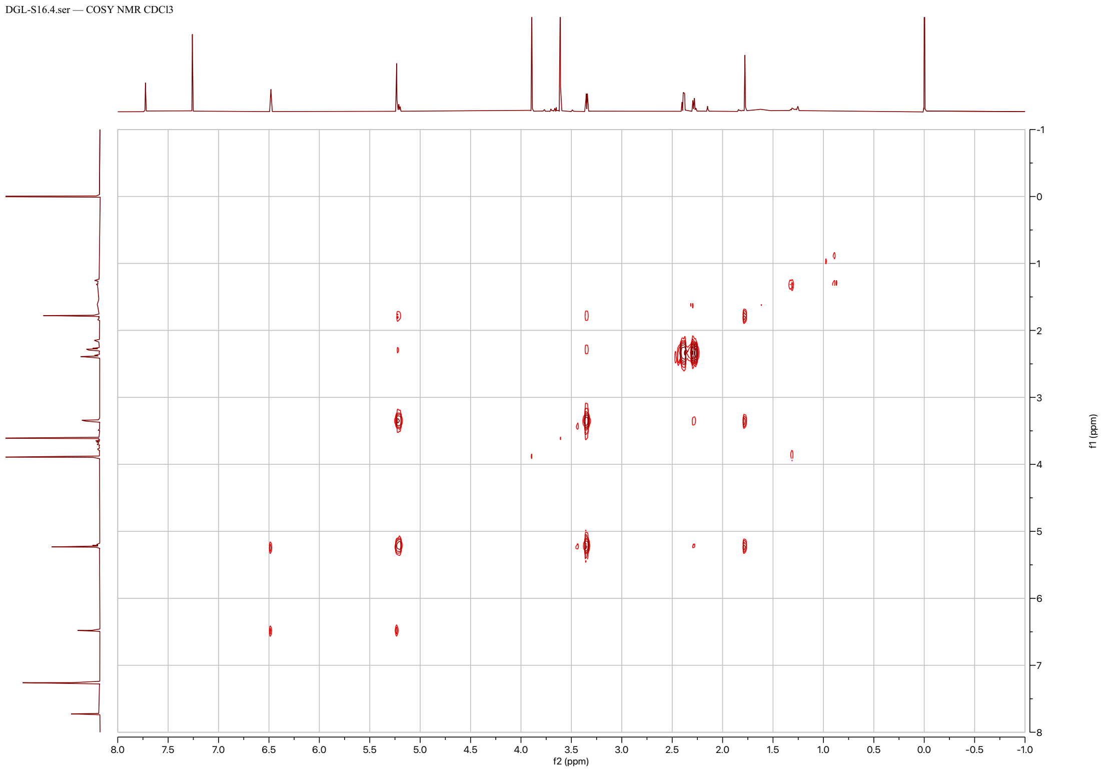

Figure S100. COSY spectrum of **11** ($CDCl_3$).

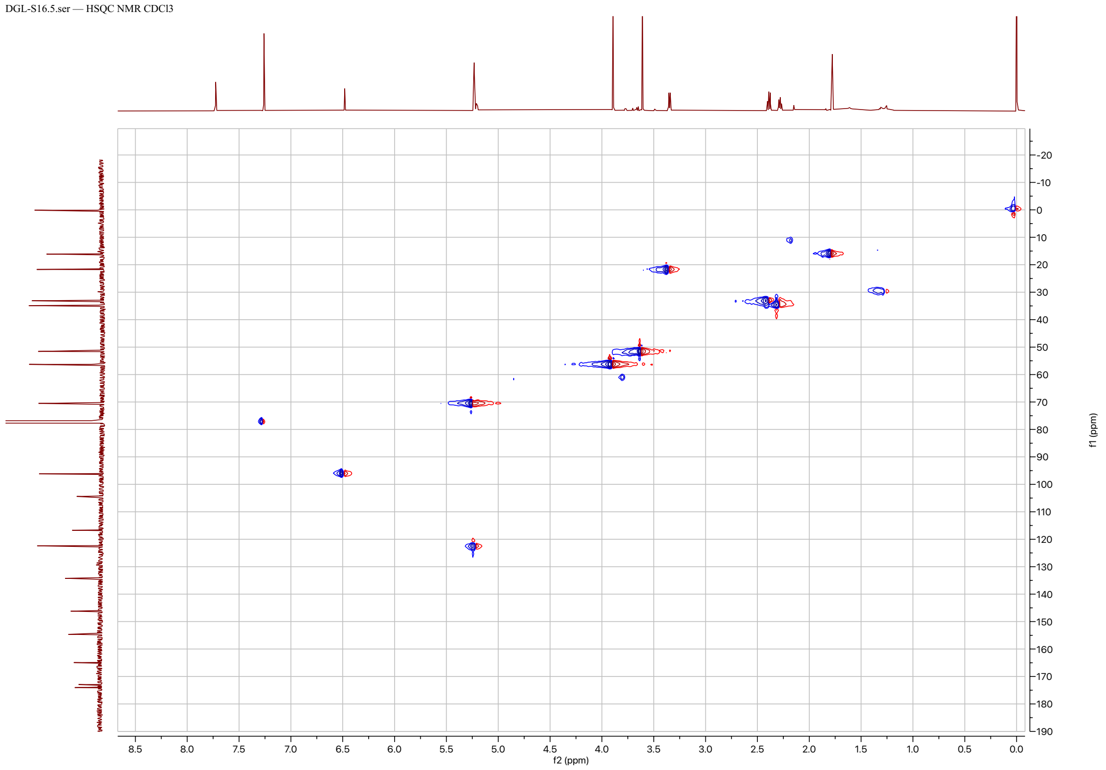

Figure S101. HSQC spectrum of **11** ($CDCl_3$).

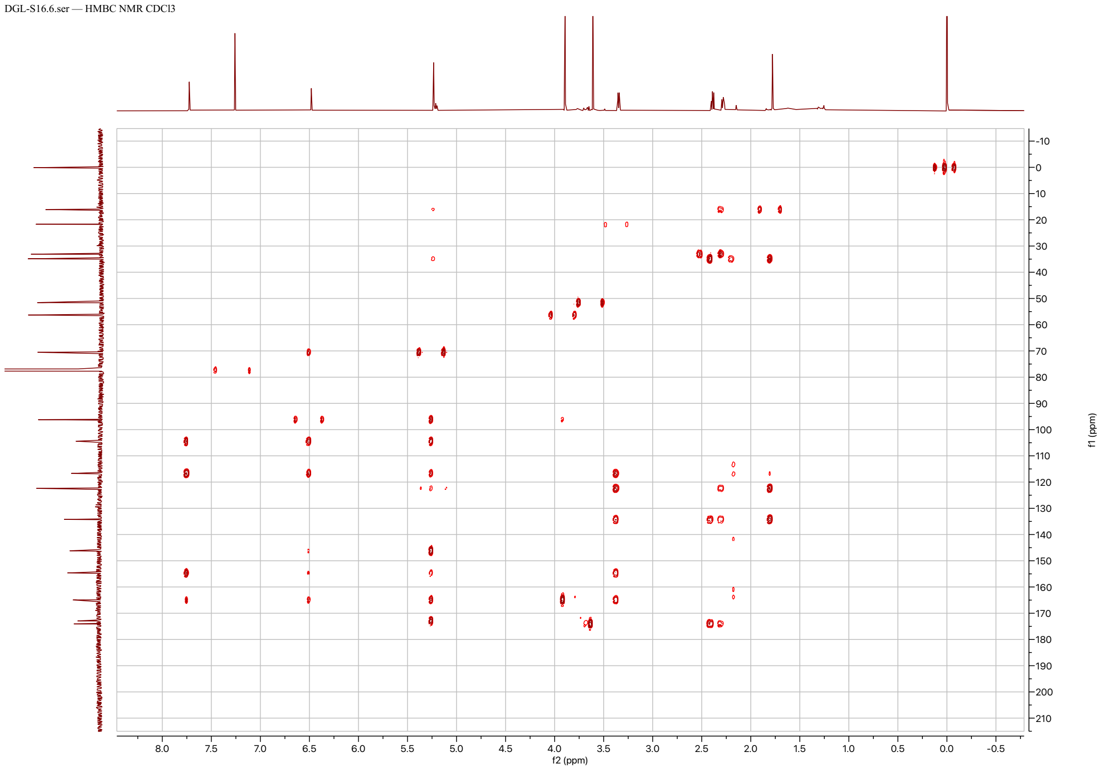

Figure S102. HMBC spectrum of **11** ($CDCl_3$).

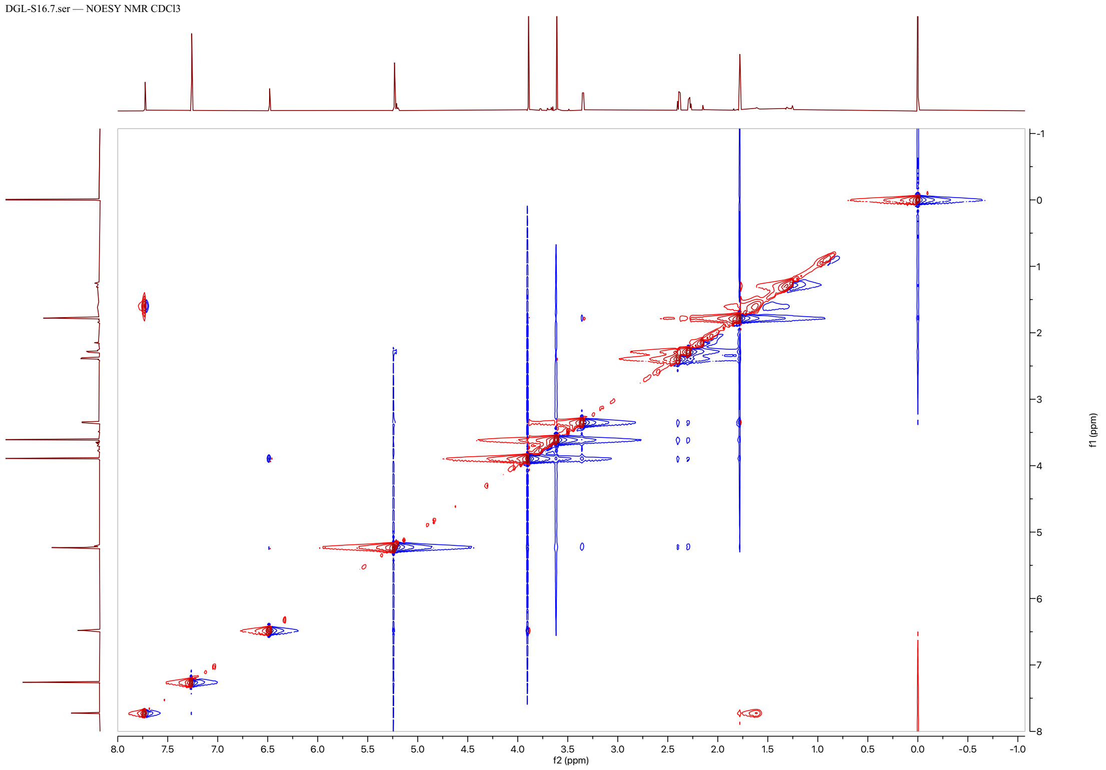

Figure S103. NOESY spectrum of **11** ($CDCl_3$).

Analysis Report

Agilent | Trusted Answers

Sample Information

**Name**	DGL-S16-NEG-02	**Data File Path**	D:\Projects\2023\Data\RCDB\DGL\20250220\DGL-S16-NEG-02.d
**Inj. Vol. (ul)**	0.5	**Method Path (Acq)**	D:\Projects\2023\Methods\General negative organic analysis method-2 .m
**Position**	P1-A1	**Acq. Time (Local)**	2/20/2025 3:47:15 PM (UTC+08:00)
**MS Type**	QTOF	**Ion Polarity**	Negative
**Instrument**	G6545B	**Version (Acq SW)**	6200 series TOF/6500 series Q-TOF (11.0.221.1)
**Operator**	SYSTEM (SYSTEM)		

Sample Spectra

- Scan (rt: 4.379 min)

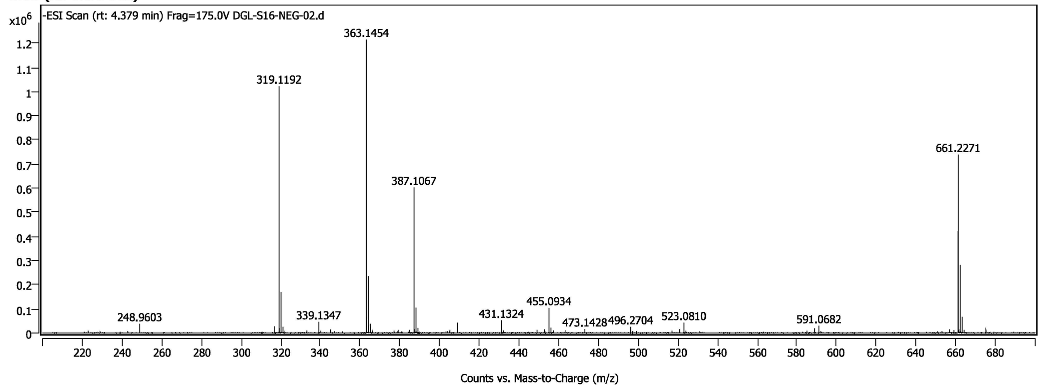

Spectrum Peaks

m/z	z	m/z (Calc)	Ion Species	Formula	Diff (ppm)	Diff (mDa)
319.1192	1	319.1187	(M-H)-	C17 H20 O6	1.57	0.5
320.1221	1	320.1221	(M-H)-	C17 H20 O6	-0.16	-0.1
321.1245	1	321.1244	(M-H)-	C17 H20 O6	0.15	0.0

Spectrum Identification Table

Formula	m/z	Mass	Species	Score	Diff (ppm)	Diff (mDa)
C17 H20 O6	319.1192	320.1264	(M-H)-	98.77	1.28	0.4
C16 H14 N7 O	319.1192	320.1265	(M-H)-	97.56	1.72	0.6
C18 H16 N4 O2	319.1192	320.1265	(M-H)-	95.05	-2.68	-0.9
C15 H18 N3 O5	319.1192	320.1265	(M-H)-	90.12	5.68	1.8
C10 H20 N6 O4 S	319.1192	320.1267	(M-H)-	89.41	0.18	0.1

MassHunter Qual 10.0
(End of Report)

MassHunter Qualitative Analysis | Page 1 of 1 | Generated at 3:58 PM on 2/20/2025

Figure S104. HRESIMS spectrum of **11**.

RCDB

样品ID	日期和时间	积分时间 (s)	扫描速度	数据间隔	起始波长	结束波长	带宽	型号#
样品S16-15	2025/2/25 13:34:50	0.05	1200.00	1.00	200.00	400.00	0.5 nm	Evo350

峰：:

nm	Abs
219.569	1.331
258.970	0.479

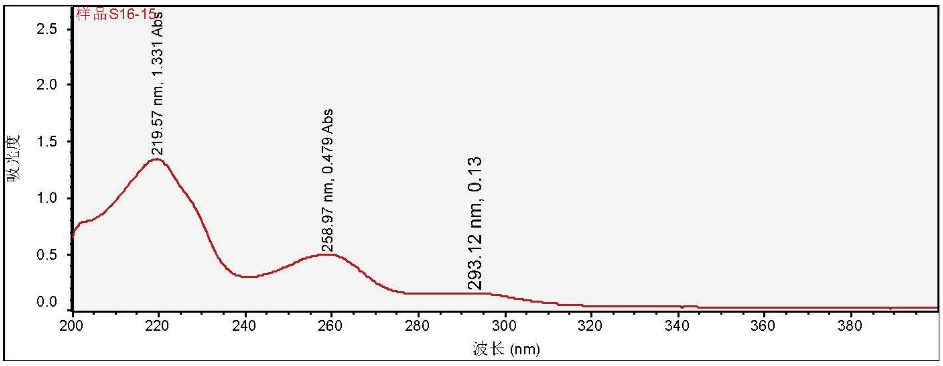

INSIGHT Software 第 1 页，共 1 页

Figure S105. UV spectrum of **11**.

The physiochemical data of compounds 10 and **11**.

Methyl -6-(4,6-dihydroxy-7-methyl-3-oxo-1,3-dihydroisobenzofuran-5-yl)-4-methylhex-4-enoate (**10**). Yellow oil; $[\alpha]_D^{25}$ = +1.0 (*c* 0.2, MeOH); UV (MeOH) λ_{max} (log ε) 218 (4.30), 260 (3.84) nm; ECD (0.20 mg/mL, MeOH) λ_{max} ($\Delta\varepsilon$) 204 (-0.10), 222 (-0.28), 236 (-0.27) nm; ^{1}H and ^{13}C NMR data, see **Table S1**; HRESIMS *m/z* 319.1192 $[M - H]^-$ (calcd for $C_{17}H_{19}O_6$, 319.1187).

Methyl (*E*)-6-(4-hydroxy-6-methoxy-3-oxo-1,3-dihydroisobenzofuran-5-yl)-4-methylhex-4-enoate (**11**). Yellow oil; $[\alpha]_D^{25}$ = -1.3 (*c* 0.2, MeOH); UV (MeOH) λ_{max} (log ε) 220 (4.45), 259 (4.01) nm; ECD (0.15 mg/mL, MeOH) λ_{max} ($\Delta\varepsilon$) 212 (0.01), 247 (-0.26) nm; ^{1}H and ^{13}C NMR data, see **Table S1**; HRESIMS *m/z* 319.1194 $[M - H]^-$ (calcd for $C_{17}H_{19}O_6$, 319.1187).
